# Early-mid Holocene Ross Sea extreme wave heights reflect sea ice retreat and ocean-atmosphere interactions

**DOI:** 10.1038/s41467-026-76311-y

**Published:** 2026-08-03

**Authors:** Shuo Wang, Ninglian Wang, Yuzhu Zhang, Carlo Baroni, Maria Cristina Salvatore, Bo Sun, Zhiheng Du, Xi Ding, Yan Zhu

**Affiliations:** 1 Shaanxi Provincial Key Laboratory of Earth Surface System and Environmental Carrying Capacity, College of Urban and Environmental Sciences, Northwest University, Xi’an, China; 2 Dipartimento di Scienze della Terra, Università di Pisa, Istituto di Geoscienze e Georisorse, Pisa, Italy; 3 Glaciology Division, Polar Research Institute of China, Shanghai, China; 4 State Key Laboratory of Cryospheric Science and Frozen Soil Engineering, Northwest Institute of Eco-Environment and Resources, Chinese Academy of Sciences, Lanzhou, China; 5 Chinese Antarctic Center of Surveying and Mapping, Wuhan University, Wuhan, China

**Keywords:** Cryospheric science, Ocean sciences

## Abstract

Antarctic coastal systems preserve valuable records of postglacial environmental change and extreme storm events. On Inexpressible Island, Ross Sea, we integrated geomorphic mapping, boulder measurements, exposure dating, radiocarbon constraints, and hydrodynamic analysis to reconstruct early to mid-Holocene storm-wave activity. Here we show that between approximately 8000 and 5000 years ago, reduced sea-ice cover, expanded open-water fetch, and stronger atmosphere–ocean coupling drove repeated high-energy storms with significant wave heights of 1–3 m during typical events and up to 4–9 m during episodic extremes. These storms transported meter-scale boulders and formed raised beach ridges, the highest ridge crest situated at 29 m above sea level (asl), reflecting postglacial sea-level fluctuation, glacio-isostatic adjustment, and local wave energy variations. Holocene storminess in the Ross Sea region exceeded present-day extremes, underscoring linkages between Antarctic coastal evolution and climate–ocean–cryosphere processes, with implications for projecting future coastal change under ongoing warming.

## Abstract (web summary)

Raised boulder beaches on Inexpressible Island record extreme storm waves of 4–9 m during the early to mid-Holocene, when reduced Antarctic sea ice and stronger ocean–atmosphere coupling drove wave energy far exceeding present-day levels.

## Introduction

In a warming world, nearly half of global coastlines are increasingly exposed to wave-related hazards, including intensified erosion, accelerated sea-ice breakup, and enhanced ice-shelf instability, all of which can influence glacier discharge and sea-level rise^1,2^. In Antarctica, extensive sea-ice cover has historically limited wave generation and coastal erosion, but recent rapid warming has reduced ice extent and prolonged open-water seasons, especially during the austral summer^3^. Over the past three decades, extreme wind speeds in the Southern Ocean have increased by 8%, and extreme wave heights by 5%^4^. Yet, the absence of long-term instrumental wave records around Antarctica constrains our ability to assess both past and future coastal wave regimes under continued warming.

**Fig. 1:**
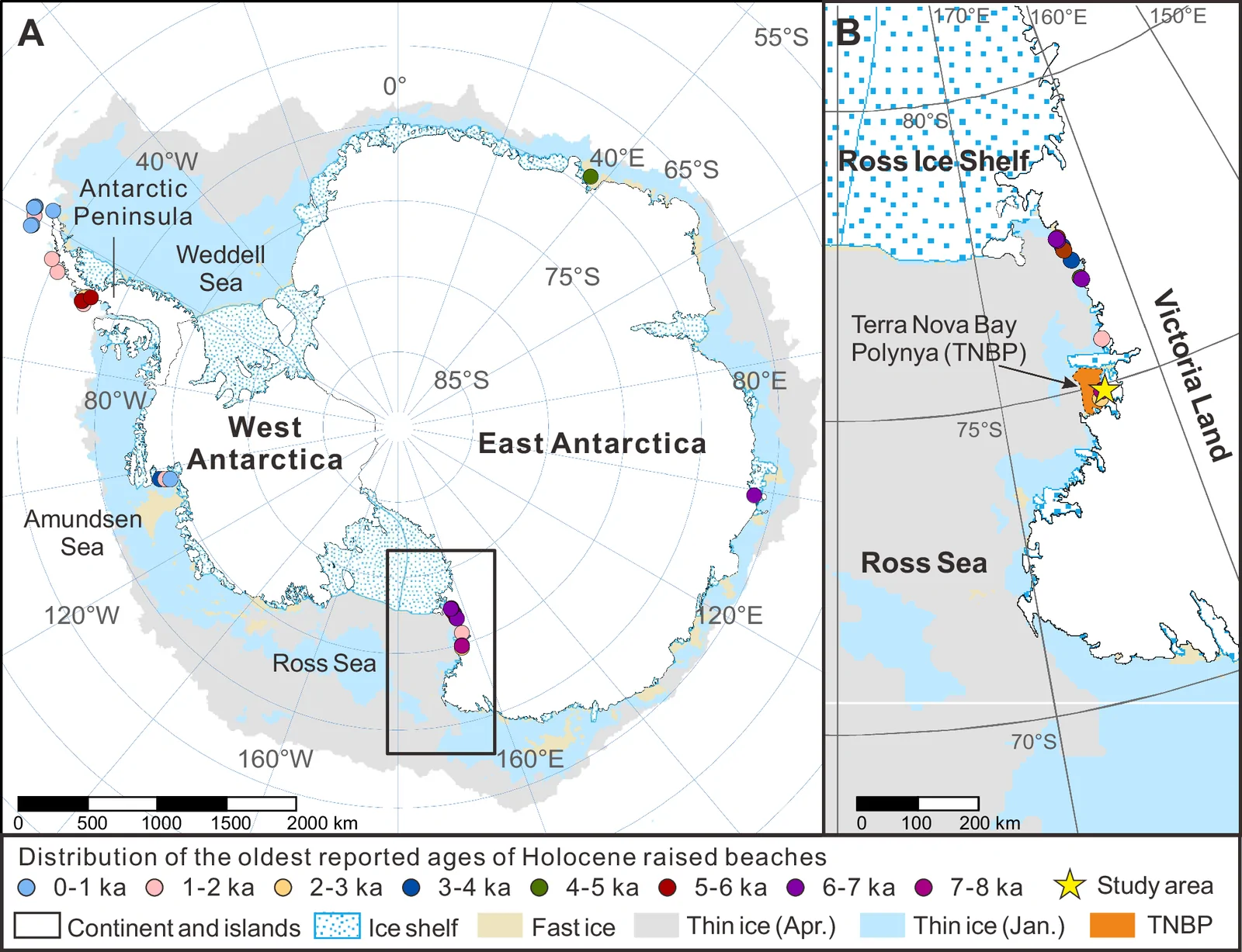
Antarctic Holocene raised-beach landforms and their environmental context. **A** Distribution of Holocene raised beaches across Antarctica, with the oldest reported ages indicated by colored circles (locations and radiocarbon ages from ref. ^6^). Antarctic coastlines and ice-shelf boundaries are from the MEaSUREs Antarctic Boundaries for IPY 2007–2009 from Satellite Radar, Version 2 (NSIDC-0709; https://nsidc.org/data/nsidc-0709/versions/2). The maps also show circum-Antarctic thin sea-ice thickness (0–0.2 m) as examples for the cold season (23 April 2020) and the warm season (23 January 2020); these layers are taken from the publicly released daily thin-ice products produced following the method of ref. ^83^. and based on AMSR-E/2 L1R brightness temperatures and NSIDC NT2 sea-ice concentration data (source: https://coas.ouc.edu.cn/pogoc/2024/1009/c31871a485693/page.htm). Antarctic fast-ice extent is from the 2020 fast-ice dataset provided at the same source. **B** Enlarged view of the study area, showing the Terra Nova Bay Polynya (TNBP) region. Colored circles indicate Holocene raised beach sites with oldest reported ages as defined in the legend of panel A. The orange shaded areas indicate the zone of ≥60% polynya occurrence frequency during the freezing period (2003–2011) as reported by ref. ^83^. The yellow star indicates the study area (Southernmost Bay, Inexpressible Island).

Geomorphological and sedimentological archives, such as raised boulder beaches, provide rare but valuable records of extreme wave events in polar coastal systems^5^. Raised boulder beaches have been identified along several Antarctic coastlines (Fig. 1), predominantly in the Ross Sea and the Antarctic Peninsula, with fewer occurrences in East and West Antarctica^6–12^. Existing evidence indicates that periods of intensified beach formation often coincided with reduced sea-ice extent and enhanced wave activity. For instance, granulometric records from Calmette Bay show increased beach development at approximately 2–1 and 6–5 ka, accompanied by more rounded clasts that reflect intensified wave reworking^6^. Similar observations from Anchorage Island and Rothera Station indicate heightened wave energy between 3.5 and 2.2 ka^9^, while records from Horseshoe Island suggest enhanced wave activity and reduced summer sea ice between 6 and 2 ka^13^. Comparable relationships between diminished sea-ice cover and increased beach formation have also been reported from other high-latitude coasts, including the Varanger Peninsula in Arctic Norway, the Kola Peninsula in northwest Russia, and Lowther Island in the Canadian Arctic^14–16^.

Antarctic raised beaches have also served as key archives for Holocene relative sea-level (RSL) reconstructions^7–10^, providing essential constraints on glacial isostatic adjustment (GIA) and ice-sheet models^12,17,18^. However, using raised beaches for RSL reconstruction is challenging because beach elevations are influenced not only by vertical land motion but also by hydrodynamic processes. Wave run-up of several meters can elevate beach deposits above still-water level^7,11^, introducing uncertainty into RSL estimates and potentially obscuring the effects of extreme events. Previous studies across Antarctica have yielded diverse RSL histories, reflecting both local variability and methodological limitations. For example, early reconstructions suggested a gradual decline in uplift rates following ice-sheet retreat^17,19^, while more recent optically stimulated luminescence (OSL) dating from Terra Nova Bay has revealed a slowdown in RSL fall to 1.8 mm a⁻¹ between 4.8 and 1.1 ka, followed by an acceleration to 14.9 mm a⁻¹ after 1.1 ka^20^. In the South Shetland Islands, mid-Holocene RSL highstands were interpreted as marine transgressions^21^. These discrepancies highlight the importance of distinguishing geomorphic and hydrodynamic influences when interpreting raised-beach elevations, and quantifying the wave energy responsible for their formation is therefore essential for understanding Antarctic coastal evolution.

Despite their significance, quantitative reconstructions of wave energy from raised boulder beaches in Antarctica remain scarce due to logistical challenges and harsh environmental conditions. Large boulders preserved on beach ridges are particularly informative because they resist transport by fair-weather waves and thus record the magnitude of extreme coastal events^22^. Globally, studies combining boulder measurements and hydrodynamic modeling have successfully reconstructed past wave heights in regions such as the Atlantic coast, Mediterranean, Caribbean, Japan, Australia, and the Gulf of Thailand^23–27^, demonstrating that accurate boulder characterization allows estimates of the flow velocities and wave heights required to mobilize large clasts^28^. In Antarctica, most studies, mainly from the Antarctic Peninsula, have relied on qualitative indicators such as sediment roundness to infer relative wave intensity^6,9,13^. Earlier studies assumed that Holocene storm-raised beaches formed 2–3 m^7,11,21^, whereas modern beaches along the Ross Sea coast can reach up to 4 m^29^, suggesting that previous estimates may have underestimated the magnitude of Antarctic extreme waves.

**Fig. 2:**
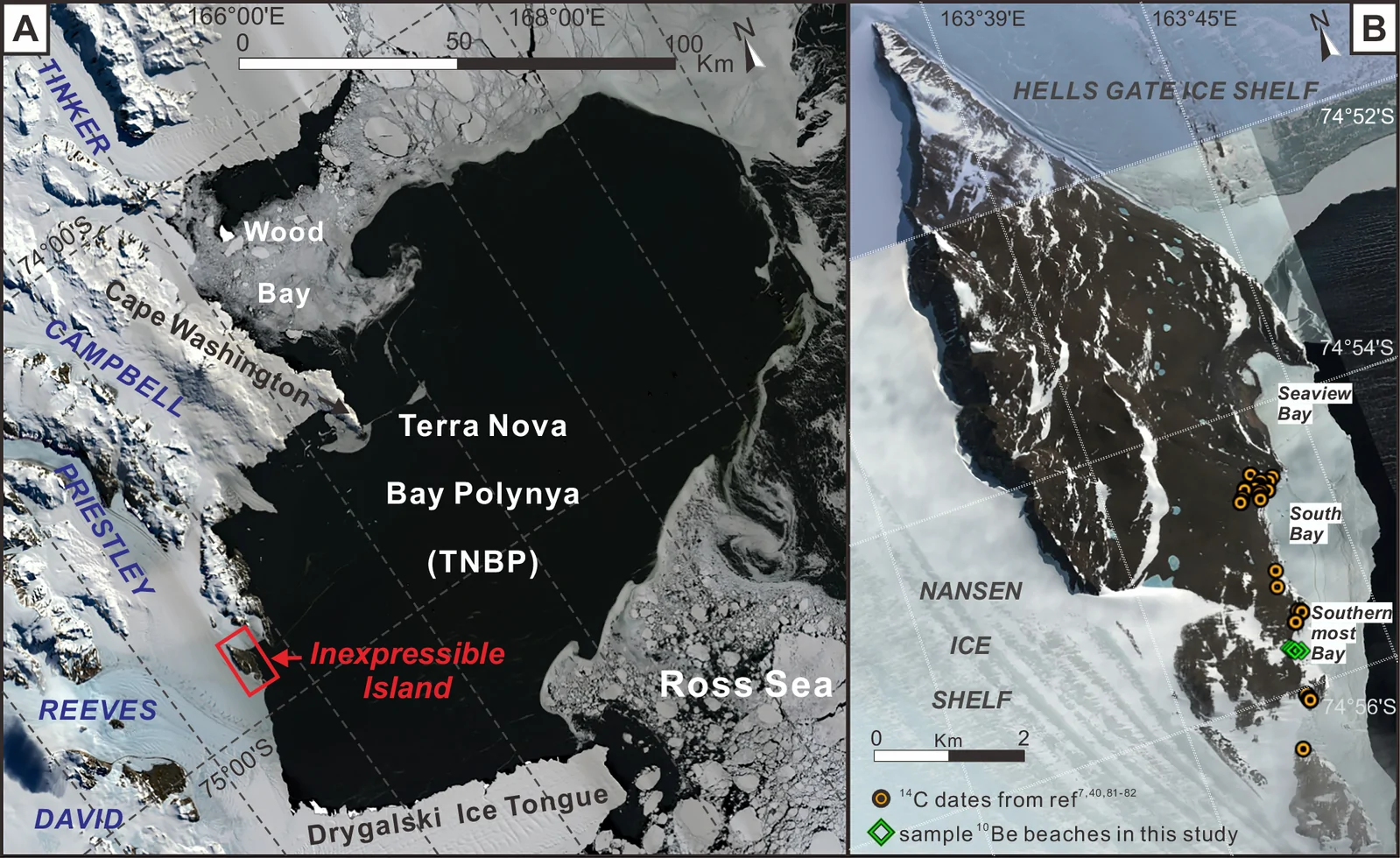
Study area and sampling locations on Inexpressible Island, Antarctica. **A** Satellite image of the Terra Nova Bay region, with nearby glaciers labeled in dark blue capital letters. The study area on Inexpressible Island is indicated by a red rectangle. Satellite imagery is from Landsat 8, 14 February 2023 (source: http://www.gscloud.cn). **B** Google Earth image (© Google Earth) showing the locations of collected samples on raised beaches across South Bay and Southernmost Bay of Inexpressible Island. Orange circles indicate previously published AMS ^14^C dating sites (Supplementary Fig. 3 and Supplementary Dataset 2), and green diamonds indicate the locations of newly collected ^10^Be exposure age samples.

To address these uncertainties, this study investigates the raised boulder beaches of Inexpressible Island, located along the western coast of Terra Nova Bay in the Ross Sea (Fig. 2). The island is directly influenced by the Terra Nova Bay Polynya (TNBP), a large and recurrent open-water area primarily maintained by strong katabatic winds from Reeves Glacier and the blocking effect of the Drygalski Ice Tongue^30,31^. The TNBP always connects to the open sea directly in summer, with its area peaking at about 5000 km^2^, while during the cold season it remains partially open, with a mean area of about 2361 km^2^ ^32,33^. These conditions enable the generation of local waves reaching 2–3 m in height even when most of the Ross Sea remains ice-covered, and allow the intrusion of Southern Ocean swells during summer when regional sea ice retreats^34,35^. Geological and paleoenvironmental evidence indicates that the TNBP has persisted for several millennia, maintaining open-water conditions that shaped coastal sediment dynamics^36–39^. Marine diatom and sediment records reveal millennial-scale variability in polynya efficiency and coastal sea-ice extent since the mid-Holocene^36^. Geomorphic and biological indicators, such as wave-formed raised beaches^8,37,38^, Adélie penguin colonies^40^, and elephant seal remains along the Victoria Land coast^39^, further suggest the long-term persistence of open-water conditions in Terra Nova Bay. Given these long-standing oceanographic and geomorphic settings, Inexpressible Island provides a well-suited natural setting for investigating the formation of raised boulder beaches and reconstructing the intensity of Holocene extreme wave events in Antarctica.

Here, we aim to quantitatively reconstruct the intensity and timing of Holocene extreme wave events that shaped the raised boulder beaches of Inexpressible Island. We focus on establishing a robust methodological framework that integrates detailed field measurements, systematic hydrodynamic analysis, and chronological constraints. Specifically, boulder dimensions (length, width, and height) are used to estimate the wave heights and flow velocities required to initiate boulder transport under various hydrodynamic conditions. Radiocarbon (AMS ^14^C) and in situ cosmogenic nuclide (^10^Be) dating are applied to constrain the formation ages of these raised beaches and the timing of past extreme wave events. By correlating reconstructed wave energy with post-glacial climate and RSL variability, this work seeks to reveal how Antarctic coastal systems have responded to Holocene environmental changes and to provide insights into the frequency and drivers of extreme wave activity.

## Results

### Raised-beach morphology

The bedrock of Inexpressible Island consists predominantly of Ordovician Granite Harbor Intrusives and Precambrian metamorphic complexes of the Wilson Terrane^7,8^. During the Last Glacial Maximum (LGM), ice inundated the island to possibly 400 m asl. Upon glacier retreat, a thin sheet of matrix-supported diamicton and erratics, known as Terra Nova Drift, was deposited, in which clasts range from angular to glacially moulded with striations^7^. Holocene raised beaches were subsequently cut into Terra Nova Drift or rest directly on bedrock, with beach sediments extending up to about 30 m asl following post-glacial isostatic uplift^8^. These beach deposits are composed mainly of granite and other intrusives, with a minor proportion of metamorphic and volcanic rocks, and their upper topographic limit is marked by a sharp and distinctive boundary where slightly stained, well-rounded pebbles and cobbles contact brown-stained, subrounded to angular, poorly sorted gravel and cobbles of the glacial drift^8^.

**Fig. 3:**
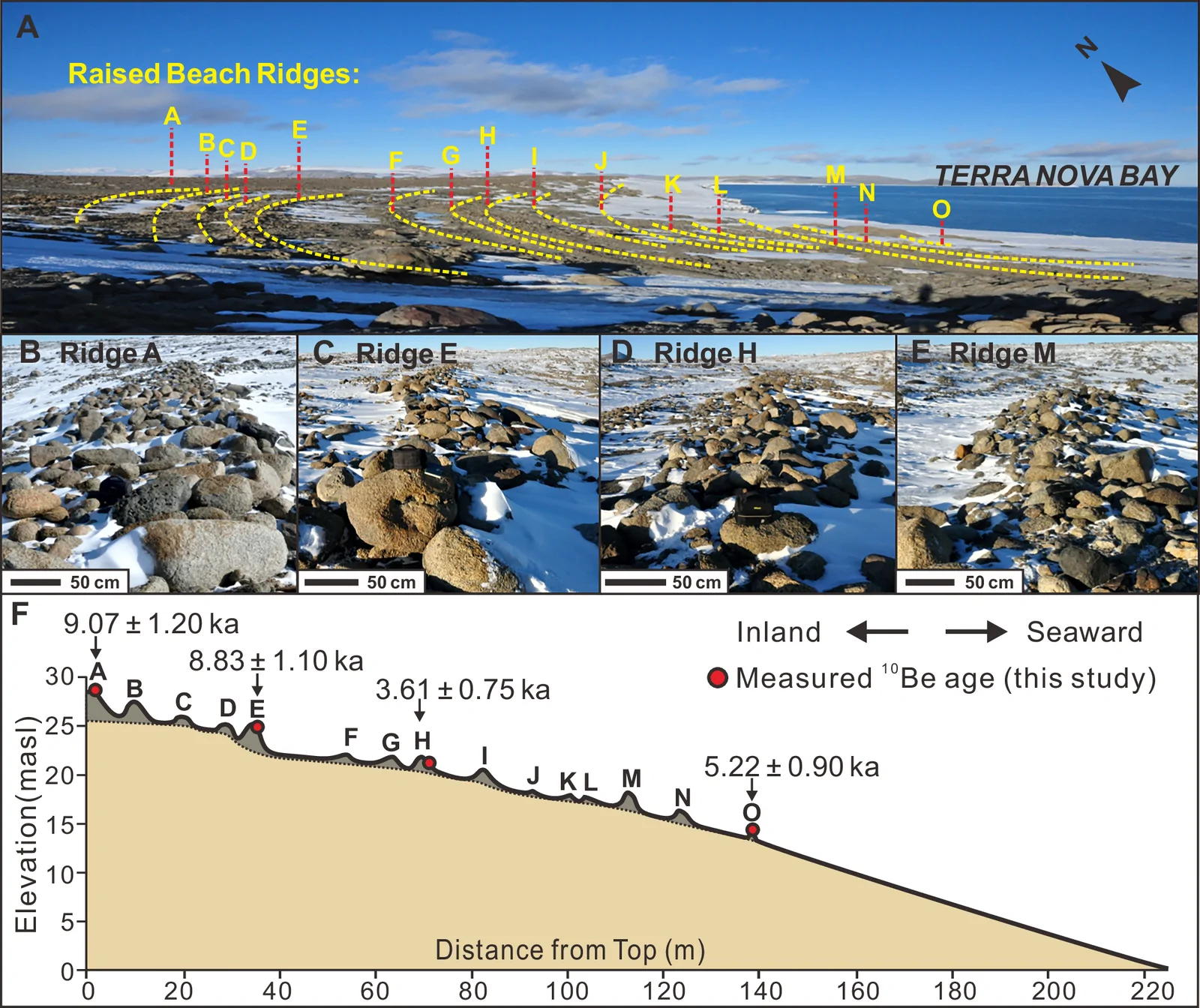
Field photographs, ^10^Be sampling sites, and topographic profile of raised beach ridges at Southernmost Bay on Inexpressible Island, Antarctica. **A** Overview photograph of the raised beach ridge sequence along Southernmost Bay, viewed from the southwest, with 15 ridges labeled A–O from inland to seaward. **B**–**E** Close-up field photographs of ridges A, E, H, and M, illustrating the well-preserved morphology and large, well-rounded boulders characteristic of these ridges. **F** Topographic profile across the beach ridge sequence (A–O), measured along a shore-perpendicular transect, with elevation (m asl) on the vertical axis and distance from the top of the ridge sequence (m) on the horizontal axis. Filled red circles mark the four ^10^Be sampling sites; annotated values are the measured ^10^Be exposure ages with 1*σ* external uncertainties. Precise GPS coordinates of all samples are provided in Supplementary Fig. 3 and Supplementary Table 3.

Within half a kilometer of Southernmost Bay coastline on Inexpressible Island, more than 15 shore-parallel beach ridges have been identified (Fig. 3). These ridges occur along a steep slope of about 10°, are spaced a few meters apart, and are situated at elevations between 14 and 29 m asl (see Supplementary Table 1). Four well-defined ridges (A, E, H, M) have reliefs of 2–3 m and extend approximately 150 m in length. Smaller, less-developed beaches (e.g., F and G), with reliefs of 1–2 m, lie between these prominent ridges, while three poorly developed ridges (J, K, L) in the middle exhibit reliefs of only 0.5 m. The arcuate, continuous ridges, aligned parallel to the coastline, are characteristic of wave-dominated morphology. Features such as spits and tombolos found at several locations on the Victoria Land coast further indicate the dominance of wave-driven deposition^37,41^. While ice push can also produce beach ridges^41^, such features tend to be sharp and irregular, with poorly sorted, angular clasts; in contrast, the ridges on Inexpressible Island are broader, smoother, more continuous, and contain well-sorted, spherical boulders, consistent with wave-formed morphology.

A total of 690 boulders from 13 raised beach ridges were measured manually using a measuring tape during fieldwork (two ridges were not surveyed due to snow cover). For each boulder, the long, intermediate, and short axes were recorded, corresponding to length, width, and height, respectively. Boulders were systematically sampled along each ridge (minimum 50 per ridge), with large boulders deliberately included to constrain maximum hydrodynamic forcing. On well-developed beach ridges with prominent relief, the average long-axis boulder size ranged from 48.2 to 73.8 cm, with the largest individual boulder reaching 160.0 cm. Even on less developed ridges, the average long axis exceeded 20.0 cm (Supplementary Table 2). The ridge-forming clasts exhibit well-rounded shapes including spheroidal, sub-spheroidal, and sub-rectangular prismatic forms (Fig. 3 and Supplementary Fig. 1). The four most prominent ridges (A, E, H, M) display relatively lower Corey Shape Index (CSI) values (0.56–0.61; Supplementary Dataset 1), and the poorly sorted mixture of clast sizes and shapes reflects deposition under chaotic, high-energy storm conditions rather than prolonged sorting. Wave buoy data from the 2017 Polynyas and Ice Production and Seasonal Evolution in the Ross Sea (PIPERS) campaign indicate a mean significant wave height (SWH) of only ~0.5 m under calm conditions in the Ross Sea^42^, with extreme events occasionally exceeding 3 m in the broader open Ross Sea^42,43^; wave energy within Terra Nova Bay would be further attenuated. The scale of the observed ridges and the size of their constituent boulders thus suggest that their formation may have required wave conditions exceeding those typical of the present-day coastal environment.

Some exceptionally large clasts were excluded from the dataset, as their angular morphology and low roundness indicate minimal wave reworking and suggest they are likely in situ glacial erratics rather than wave-transported boulders (e.g., ridge A: 330.0 × 330.0 × 60.0 cm; ridge D: 500.0 × 300.0 × 280.0 cm; ridge I: 400.0 × 185.0 × 200.0 cm; ridge G: 420.0 × 220.0 × 140.0 cm; Supplementary Fig. 2). While finer materials may have been modified or removed by cold, dry katabatic winds, our analysis focuses on the larger, well-rounded boulders interpreted to have been emplaced by high-energy wave events. These observations and methodological choices collectively support the interpretation that storm-induced extreme waves were the dominant agent shaping the raised beach ridges on Inexpressible Island.

### Dates of the raised beaches

**Fig. 4:**
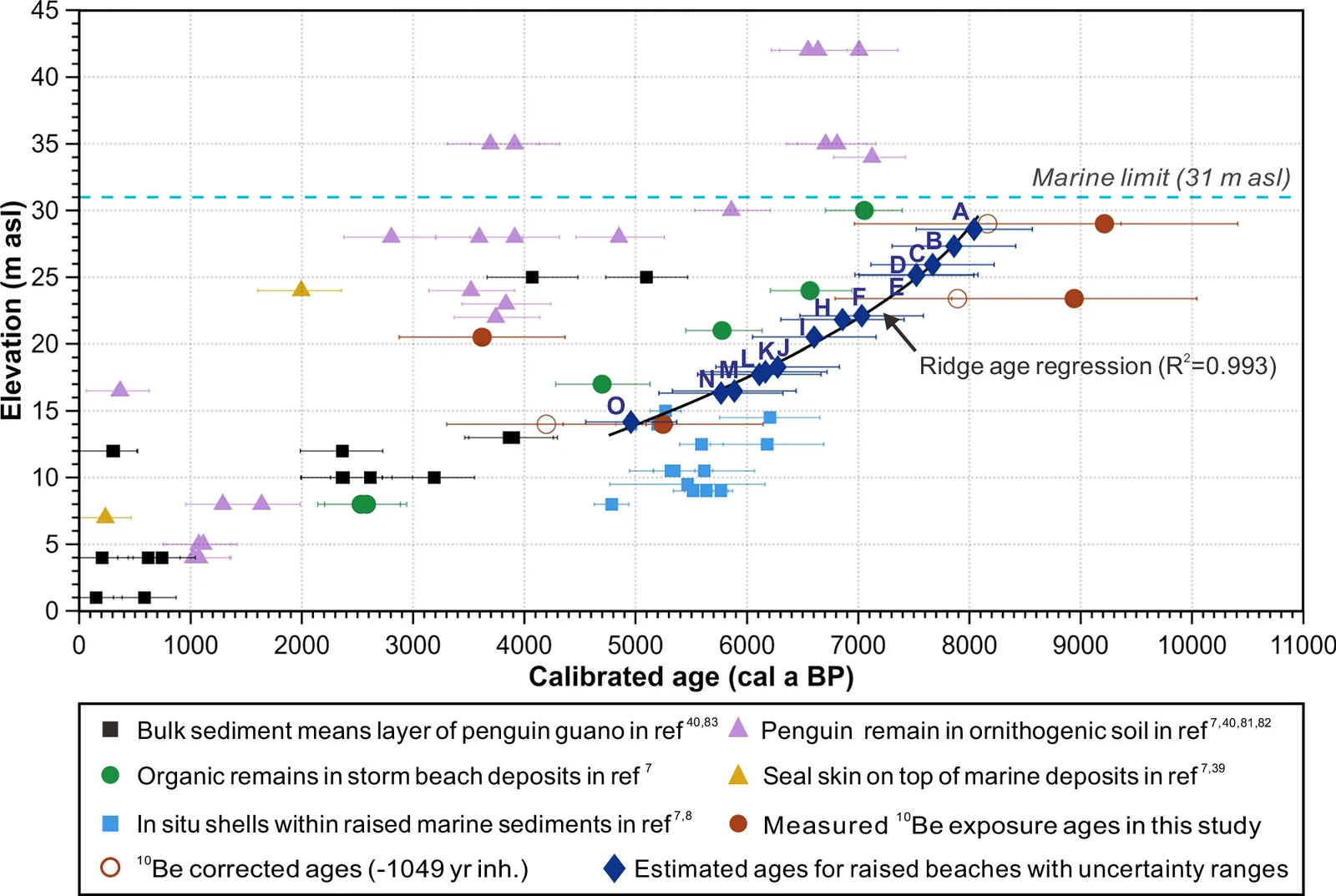
Age–elevation relationship of storm-built raised beaches at Southernmost Bay on Inexpressible Island, Antarctica. Navy blue diamonds show Bayesian fusion formation ages for ridges A–O; the solid regression line has *R*^2^ = 0.993. Brown filled circles show measured ^10^Be exposure ages; open brown circles show inheritance-corrected ^10^Be ages (−1049 a). Purple triangles show penguin remains in ornithogenic soils; black squares show bulk sediment (penguin guano layers); yellow-brown triangles show seal skin on top of marine deposits; green circles show organic remains in storm beach deposits; light blue squares show in situ shells within raised marine sediments. The dashed cyan line indicates the marine limit at 31 m asl. Error bars on ^10^Be exposure ages and Bayesian fusion formation ages represent 1*σ* uncertainties. Error bars on all AMS ^14^C ages represent 2*σ* uncertainties. ^10^Be exposure ages are listed in Supplementary Table 4; AMS ^14^C ages from previously published studies (refs. ^7,8,39,40,80–82^) are listed in Supplementary Dataset 2; Bayesian fusion formation ages for individual ridges are listed in Supplementary Table 5.

Four cosmogenic ^10^Be exposure ages were obtained from raised beach ridges on Inexpressible Island (Fig. 4). Sample information and results are summarized in Supplementary Tables 3 and 4. Apparent exposure ages from ridges A, E, H, and O are 9.07 ± 1.20 ka, 8.83 ± 1.10 ka, 3.61 ± 0.75 ka, and 5.22 ± 0.90 ka, respectively (1*σ* external uncertainties; see Methods). These represent raw modeled outputs assuming a single period of continuous exposure, prior to geological corrections described below. GIA corrections were applied to all four samples using iceTEA^44^ with the ICE-6G model. The resulting age offsets are small (10–140 a, ≤1.5% of uncorrected ages) and negligible relative to measurement uncertainties, but are incorporated for completeness; GIA-corrected ages are reported in Supplementary Table 4.

To better constrain the formation ages of the raised beaches, we compiled 48 published AMS ^14^C ages from the South Bay and Southernmost Bay of Inexpressible Island (Fig. 4; Supplementary Fig. 3; and Supplementary Dataset 2), derived from storm beach organics, seal skin overlying marine sediments, penguin remains in ornithogenic soils, bulk sediments containing penguin guano, and in situ marine shells. These different material types provide complementary chronological constraints: penguin and seal remains yield minimum limiting ages for beach emergence, since these animals only colonize land surfaces after they emerge above sea level; in situ marine shells provide maximum limiting ages, as these organisms lived when the beach was still submerged; and organic materials within well-preserved beach stratigraphy relatively closely approximate true depositional ages.

Comparison of the GIA-corrected ^10^Be ages with the compiled AMS ^14^C dataset reveals that samples appear slightly older than AMS ^14^C ages from nearby beach deposits at comparable elevations, a pattern consistent with a small inherited ^10^Be component accumulated prior to final deposition through shallow-water production or intermittent subaerial exposure during boulder transport. We note, however, that AMS ^14^C ages of organic materials from beach deposits also carry a systematic offset relative to true beach formation age, as colonization by penguins and seals necessarily postdates beach emergence. This colonization lag (~1000 ± 400 a, estimated from the offset between storm beach organics and the broader AMS ^14^C dataset at equivalent elevations) was therefore incorporated into the inheritance estimation. Using a weighted regression of GIA-corrected ^10^Be ages against lag-corrected storm beach organic AMS ^14^C ages, we estimate a mean inheritance of ~1049 a (95% CI: 0 to +2850 a), based on samples from ridges A, E, and O. The wide confidence interval reflects the limited sample size and sensitivity to the assumed colonization lag (Fig. 4); a sensitivity analysis across lag values of 0–1500 a yields inheritance estimates ranging from ~550 to ~2050 a. Sample NYD-MCB8-1 from ridge H was excluded from this estimate, as it yields an anomalously young apparent age (3.61 ± 0.75 ka) relative to both the local ^14^C chronology and the other ^10^Be samples. This anomaly is consistent with post-depositional surface disturbance, though the precise mechanism remains uncertain. Possible processes include storm waves overtopping and reworking the lower beach ridges (H–O), among other disturbance mechanisms. After applying the inheritance correction, the ^10^Be ages from ridges A, E, and O show substantially improved agreement with the AMS ^14^C chronology, and residual offsets fall within the combined external uncertainties of both methods (Fig. 4).

Formation ages for individual ridges were estimated using a Bayesian fusion approach combining inheritance-corrected ^10^Be ages, lag-corrected storm beach organic AMS ^14^C ages, and a distance-from-shoreline auxiliary regression for ridges lacking direct dating control (see Methods for full details). Stratigraphic bounds from in situ marine shells (upper age limit) and penguin/seal remains (lower age limit) were applied where available. Based on this framework, ridge A (~29 m asl, ~225 m from shore) formed at approximately 8.0 ± 0.5 ka, ridge E (~25 m asl, ~191 m from shore) at ~7.5 ± 0.5 ka, ridge H (~22 m asl, ~156 m from shore) at ~6.9 ± 0.6 ka, ridge M (~17 m asl, ~113 m from shore) at ~5.9 ± 0.6 ka, and ridge O (~14 m asl, ~87 m from shore) at ~5.0 ± 0.4 ka (Supplementary Table 5). Together, these results indicate that prominent beach ridge formation on Inexpressible Island occurred episodically during the early to middle Holocene at roughly millennial intervals, driven by a combination of RSL fall and episodic high-energy coastal processes.

This chronology corresponds well with the onset of deglaciation on Inexpressible Island, beginning around 11 ka^45^, and with the establishment of open-water polynya conditions in Terra Nova Bay by at least ~8.6 ka, as indicated by the earliest radiocarbon-dated Adélie penguin remains^40^. The presence of penguin nesting sites above the marine limit further supports ice-free conditions by at least 7.1 ka (Fig. 4 and Supplementary Dataset 2). Corroborating evidence comes from elephant seal remains: abundant desiccated skin fragments preserved on the raised beach at ~30 m asl indicate sustained occupation of the study area during the early Holocene^39^, consistent with ice-free coastal access along the Victoria Land coast at that time.

### Reconstruction of storm events

We estimated the wave heights required to initiate boulder transport on the beach ridges of Inexpressible Island (Supplementary Table 6 and Supplementary Dataset 3). Angular, oversized boulders with low roundness were excluded from the analysis, as these are more likely to represent reworked glacial till than wave-transported material. The ridge-forming clasts are predominantly granitic, broadly consistent with the local Granite Harbor Intrusive bedrock and the modern beach sediments observed during fieldwork, suggesting a local nearshore provenance following ice retreat and limited transport distances.

Specifically, critical wave height thresholds were calculated for sliding, rolling/overturning, and saltation/lifting modes across the full size distribution of boulders on each ridge. Wave parameters were calculated for the P5–P95 percentiles, where P50 represents typical formative conditions, and P95 captures extreme events (Supplementary Fig. 4). The results reveal systematic relationships between ridge morphology, boulder size, and wave energy requirements (Supplementary Fig. 4 and Supplementary Dataset 3). For sliding, median boulders (P50) require wave heights of 0.73–2.56 m across all ridges, while the largest boulders (P95) demand 1.19–4.19 m. Rolling/overturning requires substantially higher energy: P50 thresholds range from 0.96–5.08 m, and P95 thresholds reach 4.62–8.90 m for the most developed ridges (A, E, H, M, N). Saltation/lifting would demand unrealistically large waves for meter-scale boulders (>26 m for some P95 cases), indicating a negligible role in transporting the largest clasts.

The broad size distribution within each ridge argues against formation by a single extreme event and instead reflects cumulative deposition from multiple storms of varying intensity. Smaller boulders (P5–P25) record frequent, lower-energy storms, while the largest clasts (P75–P95) preserve rare, extreme events. The most prominent ridges yield notably similar P95 thresholds, suggesting comparable extreme storm intensities among ridges formed during different periods of the Holocene: sliding/rolling wave heights are 4.09/8.90 m for ridge A (P95 clast: 125.0 × 90.0 × 40.0 cm), 4.19/8.16 m for ridge E (130.0 × 90.0 × 50.0 cm), 2.84/4.95 m for ridge H (180.0 × 60.0 × 40.0 cm), and 2.87/4.62 m for ridge N (90.0 × 60.0 × 45.0 cm). Taken together, ridge construction was driven by a spectrum of storm events, with typical formative conditions of ~1–3 m and episodic extremes of ~4–9 m significant wave height.

## Discussion

### Deglaciation and GIA history in Terra Nova Bay

**Fig. 5:**
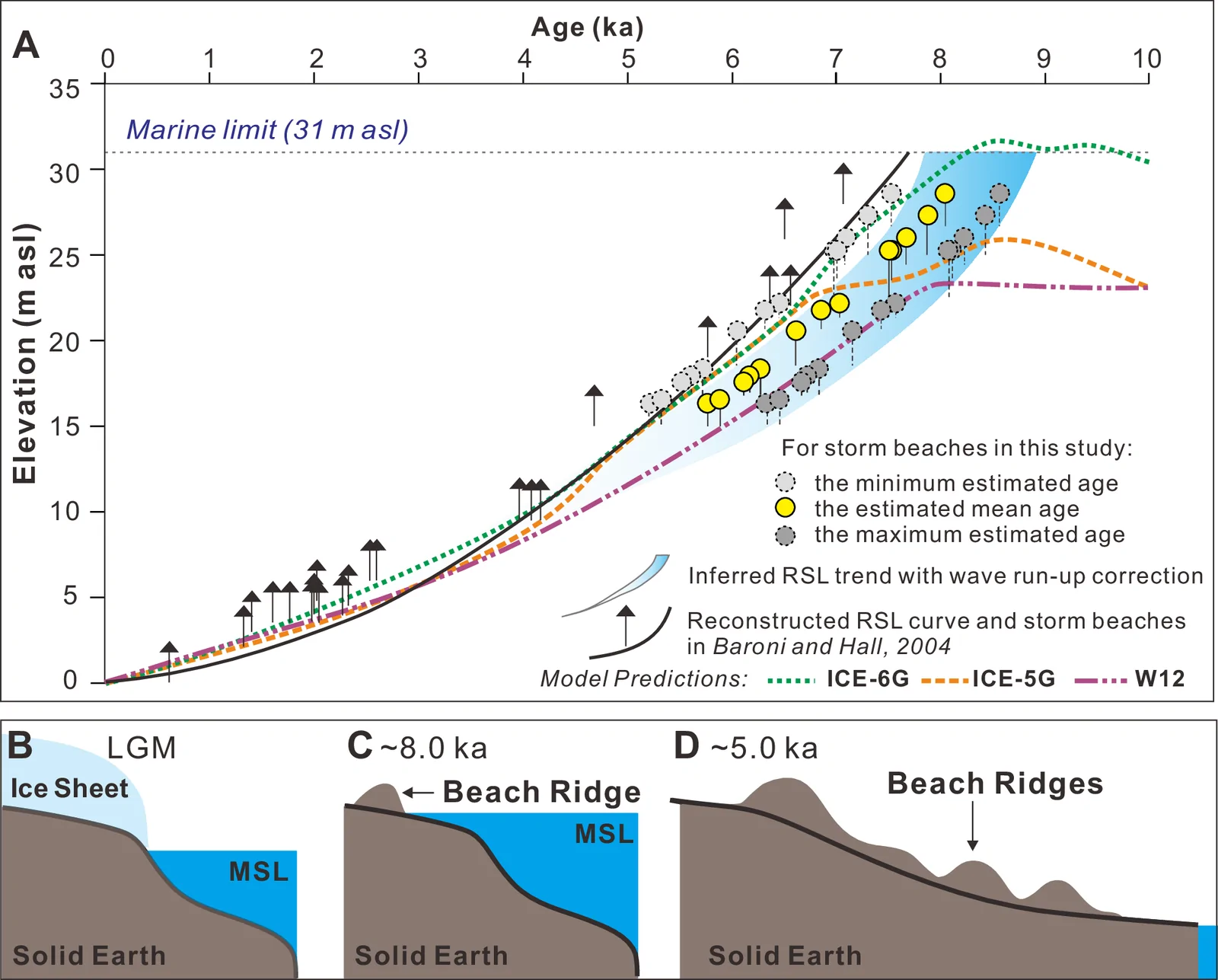
Postglacial coastal evolution and relative sea-level (RSL) changes inferred from storm-built ridges on Inexpressible Island, Antarctica. **A** Inferred RSL trend reconstructed from the formation ages and elevations of storm-built beaches. For storm beaches in this study: dashed light gray circles indicate the minimum estimated age; yellow filled circles indicate the estimated mean age; dashed dark gray circles indicate the maximum estimated age. Downward vertical bars beneath each circle indicate wave run-up elevation corrections (P50 typical wave heights calculated using the sliding threshold transport mode), with bar length representing the magnitude of the correction; the base of each bar represents the corrected RSL position. The blue shaded envelope represents the uncertainty range of the inferred RSL trend. Black triangles show storm beach data points from ref. ^7^, with downward vertical bars indicating a uniform 2 m wave run-up correction applied in that study; the black solid line shows the RSL curve reconstructed in ref. ^7^. (recalculated using the Marine20 calibration dataset and reservoir corrections^10^). Glacial isostatic adjustment (GIA) model predictions are shown for ICE-6G (green dotted; ref. ^18^), ICE-5G (orange dashed; ref. ^19^), and W12 (purple dash-dot; ref. ^17^). The dotted gray line indicates the marine limit at 31 m asl. **B** Deglaciation stage during the Last Glacial Maximum (LGM) with no preserved ridges, showing the ice sheet grounded at the coast and mean sea level (MSL) position. **C** Formation of earliest ridges during marine highstand under storm-wave conditions (~8.0 ka). **D** Progressive postglacial uplift and development of successive storm-built ridges during episodic high-energy events (~5.0 ka).

During the LGM, outlet glaciers in the region coalesced into a piedmont glacier that reached ~400 m asl, merging with grounded ice in the Ross Sea^7,8,46^. Our reconstruction of the raised beach sequence from Southernmost Bay of Inexpressible Island provides chronological constraints on the glacial retreat history of Terra Nova Bay and its subsequent GIA. Overall, our results are consistent with the generally accepted sea-level curve^7^, based on radiocarbon dating of ~100 organic samples. However, the ages of raised beaches above ~18 m asl (Ridges A–J; 8.0–6.3 ka) tend to indicate an earlier timing of glacier retreat and beach emergence than the reference curve, by approximately 1.0–0.5 ka (Fig. 5). Our ^10^Be exposure ages indicate that the oldest preserved beach ridge, situated ~29 m asl, formed at ~8.0 ± 0.5 ka, implying that the coastline of Terra Nova Bay had already become ice-free and exposed to marine processes by that time. This interpretation is supported by independent evidence, including penguin guano deposits at ~34 m asl on Inexpressible Island (7.1 ± 0.3 cal ka BP, 2*σ*)^40^. We infer that glacier retreat began in the early Holocene, creating open-water conditions that facilitated the formation of the earliest beach ridges.

This conclusion aligns with recent records from the southern Transantarctic Mountains and the Scott Coast, which indicate rapid grounding-line retreat and thinning between 9 and 8 ka^47^. Our data indicate that Terra Nova Bay may also have experienced open-water conditions by ~8 ka, suggesting glacier retreat may have occurred earlier than indicated by some prior estimates. These findings support the hypothesis of an early and rapid Holocene deglaciation in this sector of Antarctica, during which most of the grounded ice in the western and central Ross Sea was removed in less than two millennia^45,48–50^.

The raised beach ridges developed following deglaciation of Terra Nova Bay, when the coastline became exposed to open-marine conditions and subjected to wave reworking. Their formation reflects the combined influence of RSL fluctuations, sediment availability, and high-energy storm-wave events. Reconstructing the wave conditions required to build these ridges therefore offers valuable insights into coastal evolution following deglaciation. Well-developed ridges typically show a relative relief of ~2–3 m and contain boulders exceeding 1 m in diameter—hallmarks of high-energy storm events. For ridge A, situated at 29 m asl, the P50 sliding threshold indicates that typical formative waves exceeded ~2 m. Taking the water level during ridge formation as the sum of contemporaneous sea level and wave run-up height derived from the P50 sliding estimate, we infer that post-deglacial coastal uplift at this site was approximately 27 m relative to present sea level. By integrating wave-height reconstructions from multiple ridge levels with chronological data, we find that, although uncertainties in storm energy and formation age limit the precision of our RSL curve, the overall pattern aligns with the W12^17^ GIA model as well as ICE-5G^19^ and ICE-6G^18^. Higher or lower assumed storm-wave energies would proportionally modify the inferred rates of postglacial rebound.

### Early-mid Holocene extreme waves and climate–ocean coupling

**Fig. 6:**
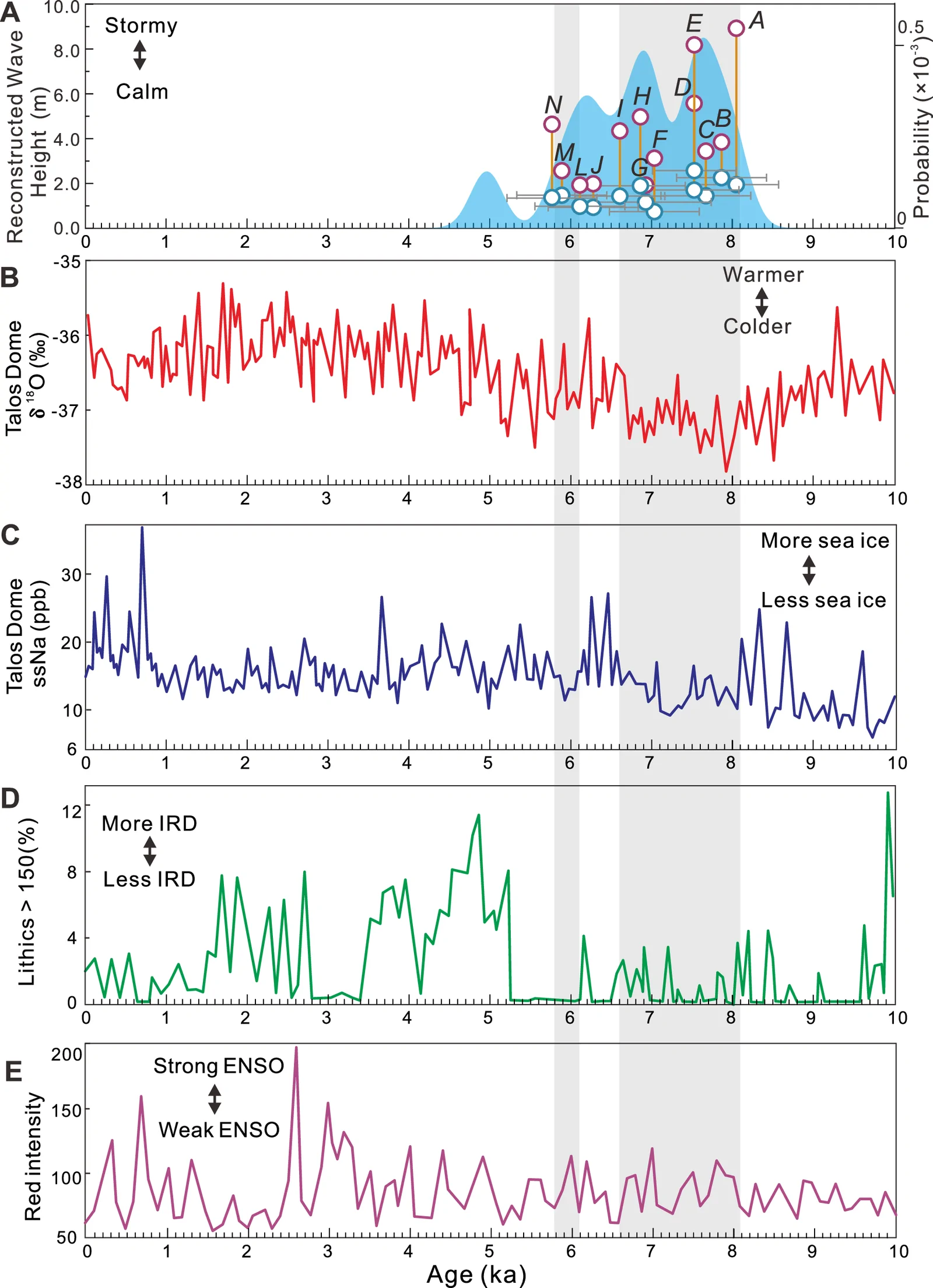
Forcing factors and chronology of extreme wave events on Inexpressible Island, Antarctica. **A** Reconstructed wave heights and probability distribution of storm events. Open circles with blue outlines indicate typical wave heights (sliding threshold, P50); open circles with purple outlines indicate maximum wave heights (rolling threshold, P95); horizontal error bars represent 1*σ* age uncertainties derived from Bayesian fusion formation ages (Supplementary Table 5). The blue shaded curve shows the probability density distribution of storm events (right y-axis, Probability × 10^−3^). Gray shading indicates periods of inferred storm-wave activity. **B** δ^18^O record from Talos Dome as a proxy for local temperature variability^36^. **C** Sea-salt sodium (ssNa) record from Talos Dome as a coastal sea ice proxy^36^. **D** Ice-rafted debris (IRD) from the Antarctic Polar Front^53^. **E** Red color intensity from Laguna Pallcacocha, Ecuador, as a proxy of ENSO intensity^58^.

Although our reconstruction of the raised beaches on Inexpressible Island is affected by dating uncertainties and cannot resolve centennial-scale variability, the overall pattern indicates that wave activity during the early to mid-Holocene was not random but regulated by coupled ocean–ice–atmosphere dynamics in the western Ross Sea region (Fig. 6).

Based on boulder size and beach ridge morphology, we infer that between approximately 8 and 5 ka, the coastal environment of Inexpressible Island experienced a prolonged period of enhanced storminess. Ridge morphology and clast-size distributions indicate that construction involved repeated storm events of varying intensity, from typical conditions requiring wave heights of roughly 1–3 m to episodic extremes capable of entraining the largest boulders at 4–9 m under sliding and rolling thresholds. The recurrence of comparable extreme thresholds across the most well-developed ridges suggests that such high-energy events were not isolated incidents but a persistent feature of this interval, driving progressive ridge progradation over millennial timescales. Individual ridges are therefore best interpreted as cumulative constructional surfaces built by repeated storm episodes rather than single catastrophic events, with clast size recording the minimum wave energy threshold rather than storm frequency. Their preservation reflects the interplay between episodic high-energy emplacement and GIA-driven emergence, which progressively lifted each ridge beyond the reach of successive wave activity without requiring prolonged post-storm quiescence.

To evaluate the plausibility of these reconstructed values, we compared them against modern observational and modeled data specific to the TNBP. Within the TNBP, the PIPERS project recorded significant wave heights of ~2 m during strong katabatic wind events (SWIFT drifters, April 2017)^34^, and numerical simulations of ten intense wind events between 2016 and 2021 yield typical values of ~3 m, with maxima occasionally exceeding 5 m under ice-free conditions^35^. The reconstructed wave heights required to mobilise the largest ridge-forming boulders (4–9 m) therefore exceed what is routinely observed in the modern TNBP, and only overlap with the uppermost tail of modeled ice-free scenarios, suggesting that our estimates are hydrodynamically plausible while remaining larger than present-day conditions.

This interpretation is independently supported by field observations on the modern active beach at Inexpressible Island, where dominant clast sizes of approximately 20 cm yield estimated transport thresholds of ~0.7 m (sliding) and ~1.2 m (rolling). These thresholds are comfortably exceeded by the ~2 m significant wave heights recorded during strong katabatic wind events in the TNBP, confirming that our hydrodynamic equations produce physically reasonable results under present-day conditions. The close correspondence between modern beach transport thresholds and observed wave heights, set against the substantially higher thresholds of the paleo-ridges, further supports our interpretation that wave energy during the early to mid-Holocene was larger than present, reflecting reduced sea-ice cover, expanded open-water fetch, and intensified atmosphere–ocean coupling.

To better understand the climatic and oceanographic context of this enhanced wave activity, we compared our reconstruction with regional paleoenvironmental records documenting coeval variations in sea ice, oceanic heat transport, and atmospheric circulation (Fig. 6). Between ~11.8 and 8.2 ka, the TALDICE ice core record shows persistently low sea-salt sodium (ssNa) concentrations^36^, indicating decreased seasonal sea-ice formation and an expanded open-water area in the Ross Sea^51,52^. Concurrently, elevated δ¹⁸O values reflect a relatively warm atmospheric regime^36^, further suppressing sea-ice growth. This combination of reduced sea ice and a warming climate likely created favourable conditions for extended open-water fetch and enhanced wave generation, consistent with boulder ridge construction peaking around 8 ka. After 6.6 ka, storm-built ridge formation declined, but briefly intensified again after 6 ka, suggesting a transient expansion of sea-ice extent that may have locally suppressed wave activity (Fig. 6A–C). Ice-rafted debris (IRD) records similarly document an inverse relationship between storm-wave intensity and sea-ice coverage, with enhanced wave activity corresponding to warmer sea-surface temperatures and reduced iceberg discharge^53^ (Fig. 6D). This pattern is further corroborated by the initial colonization of Terra Nova Bay by Adélie penguins at ~8.6 ka^40^, indicating the establishment of a persistent polynya and reduced summer sea ice that enabled continuous access to marine food resources^40,54^. Taken together, these geological and ecological records indicate that the early Holocene Ross Sea was characterized by a seasonally open, dynamic marine environment, broadly analogous to conditions now emerging under modern anthropogenic warming.

Oceanic heat transport likely amplified these environmental shifts. High abundances of *Fragilariopsis kerguelensis* in marine sediment cores^36^, coinciding with low ssNa concentrations, indicate increased incursions of Circumpolar Deep Water (CDW) into the Ross Sea^55^, potentially driving retreat of the Ross Ice Shelf and the formation of large embayments such as the Joides Basin. The resulting expansion of open water would have increased fetch and nearshore wave energy^56^. Seasonal stratification of the upper water column, driven by the presence of seasonal ice in spring and anomalously warm surface water in summer, would have strengthened vertical thermal gradients and air–sea energy exchange, reinforcing wave development under storm conditions^57^.

Atmospheric variability, particularly associated with ENSO, also likely played a major role in modulating storm intensity. The formation phases of major ridges at approximately 7.5, 7 and 6 ka coincide with periods of enhanced ENSO variability recorded in tropical Pacific archives^58^ (Fig. 6E). During El Niño phases, anomalously warm tropical Pacific sea-surface temperatures (SSTs) intensify the meridional temperature gradient, strengthening the Hadley Cell and the subtropical jet stream. This configuration promotes an equatorward shift in mid-latitude storm tracks, increased poleward heat transport, and ultimately reduced sea-ice extent in the South Pacific sector^59^.

The sensitivity of the Inexpressible Island storm beaches to climate fluctuations is further linked to their proximity to the TNBP. While katabatic winds are the primary driver of this polynya, the intrusion of warmer, moisture-laden air from lower latitudes of the Pacific Ocean can expand the polynya’s area and enhance wave activity^31,60^. Such intrusions have been attributed to topographic steering, which deflects extratropical cyclones and associated moisture plumes eastward toward the Amundsen Sea, making the Ross Sea one of the least frequently affected regions by atmospheric rivers^61^. Although these atmospheric rivers do not directly affect the TNBP, the associated warmer and more moist air masses can intensify katabatic winds through interactions with synoptic or mesoscale cyclones, increasing offshore wind speeds and sea-ice drift in coastal polynyas around Antarctica^62,63^. A modern analogue of this mechanism was observed during 26–29 April 2017, when two low-pressure systems moving eastward north of Victoria Land and the Ross Sea injected lower-latitude air into the region. This resulted in a temperature increase of up to 7 °C in the TNBP, along with a significant decrease in sea-ice concentration along the northwestern edge of the Ross Sea and Terra Nova Bay^33^. Such mechanisms likely operated more frequently during the early to mid-Holocene, amplifying storm-driven wave energy along the coast.

In summary, the integration of geomorphic, ecological, oceanographic, and atmospheric evidence indicates that extreme wave events during the early to mid-Holocene were driven by the combined effects of reduced sea ice, intensified oceanic heat transport, and atmospheric teleconnections such as ENSO, which collectively enhanced katabatic winds and wave energy. These processes generated powerful storm waves capable of constructing the large boulder ridges on Inexpressible Island and substantially modifying the coastal landscape. Although chronological uncertainties and limitations in hydrodynamic estimation persist, the reconstructed storm events offer valuable insights into postglacial coastal evolution, polynya variability, and the sensitivity of Antarctic nearshore environments to climate forcing.

## Methods

### Experimental design

The objective of this study was to reconstruct early-mid Holocene extreme wave events and associated coastal geomorphology on Inexpressible Island, Ross Sea, and to estimate the postglacial RSL changes and GIA. The study design comprised the following components: field surveys to map raised beach ridges and measure boulder dimensions, boulder shape analysis to characterize clast morphology, in situ ^10^Be cosmogenic dating to constrain ridge formation ages, Bayesian fusion chronology to refine age estimates, and hydrodynamic calculations to estimate critical wave heights for boulder transport. Field measurements, laboratory analyses, and hydrodynamic calculations were performed following prespecified procedures to ensure reproducibility and consistency across all ridges examined.

### Field investigation

A two-month field investigation was conducted on Inexpressible Island from January to February 2023. Photographs and high-resolution satellite images were first used to identify and delineate the locations of raised beaches. High-precision Real-Time Kinematic GPS (RTK) surveys were then carried out to measure the elevation, geographic coordinates, and topographic profiles of the beach ridges. The hillslope gradient (~10°) was calculated from elevation differences measured along RTK survey profiles, which provided centimeter-level accuracy in both horizontal and vertical positioning. Measurements of boulders were collected from raised beaches along the Southernmost Bay of Inexpressible Island, on the western coast of Terra Nova Bay. A total of 690 boulders were measured from 13 of the 15 identified beach ridges (two ridges could not be surveyed owing to snow cover), with elevations ranging from 14 to 29 m asl. The length, width, and height of each clast were measured using a tape measure, and rock chips were extracted using a cutting machine for laboratory density determination based on Archimedes’ principle.

### Boulder shape analysis

Multiple descriptive indices of particle shape are utilized to reflect the high-energy hydrodynamic events on the raised beaches of Inexpressible Island. The maximum projection sphericity (MPS), $\root 3 \of {{S}^{2}/\left({IL}\right)}$, which indicates the balance of drag and gravitational forces acting on a particle immersed in a fluid, quantifies particle sphericity^64^. The Oblate-Prolate Index (OPI), defined as 10×[(*L*-*I*)/(*L*-*S*)-0.5]/(*S*/*L*), signifies the oblateness versus prolateness of the boulder; OPI becomes more negative for more disc-like boulders and more positive for thin rods^65^. Additionally, the Corey Shape Index (CSI), $S/\sqrt{{IL}}$, describing sphericity, and the Disc-Rod Index (DRI), (*L*-*I*)/(*L*-*S*), characterizing disc-rodness, are used to plot a triangular shape diagram; these two shape indices represent the three end-members of shape: spheres, discs, and rods^66^.

### In situ ^10^Be dating

Four samples were obtained from four beach ridges: Ridges A, E, and H (selected for their largest relief) and Ridge O (selected as the ridge closest to the modern coastline). The largest boulders exhibiting obvious polished surfaces, either stable on the trough bottom or firmly embedded in the beach ridges, were preferentially sampled^67^. Samples were collected exclusively from the gentle-slope surfaces to minimize issues related to reworking and partial shielding. The sampling location (latitude, longitude, and altitude) was recorded using an RTK instrument. The thickness of the sample taken and the size of the boulder (length, width, and height) were measured (see Supplementary Table 3). The dating targets were prepared in the Terrestrial Cosmogenic Nuclide (TCN) Preparation Laboratory of State Key Laboratory of Cryospheric Science, Northwest Institute of Eco-Environment and Resources, Chinese Academy of Sciences in Lanzhou, China. Quartz grains were extracted using the experimental procedure as described in ref. ^68^, which was modified from ref. ^69^. A ^9^Be carrier solution with a concentration of 935.8 μg g⁻¹ was added to each sample (~0.3 g) prior to dissolution. The ^10^Be/^9^Be ratios were measured on a 3MV Accelerator Mass Spectrometer at Xi’an AMS Center, Institute of Earth Environment, Chinese Academy of Sciences, using the 07KNSTD standard. Samples were processed in batches of nine, with one procedural blank included per batch. The four samples reported in this study (NYD-MCB1-1, NYD-MCB5-1, NYD-MCB8-1, and NYD-MCB-R1) belong to the same processing batch, for which the procedural blank (W-BLANK2) yielded a ^10^Be/^9^Be ratio of 0.731 × 10^−^^15^. The measured ^10^Be/^9^Be ratios were corrected for procedural blank contribution using the corresponding batch blank (W-BLANK2) and converted to ^10^Be concentrations in quartz for final apparent exposure age calculation. Topographic shielding was calculated using field records and was also compared with results from the Python tool in ref. ^70^. Exposure ages were calculated using the University of Washington online calculator^71^ (http://hess.ess.washington.edu/) with the LSDn scaling model^72^ and the default global production rate dataset derived from ref. ^73^. The reported uncertainties are 1*σ* external errors, which include analytical uncertainties, production rate uncertainties, and other sources of systematic error incorporated by the calculator.

### Bayesian fusion chronology for beach ridge formation ages

Formation ages for individual beach ridges were estimated using a multi-source Bayesian fusion framework implemented in MATLAB (seeSupplementary Dataset 4 for the script), integrating inheritance-corrected ^10^Be exposure ages, colonization-lag-corrected storm beach organic AMS ^14^C ages, and a distance-from-shoreline auxiliary regression.

Prior to fusion, storm beach organic AMS ^14^C ages were corrected for colonization lag by adding 1000 a (1σ: 400 a) to each raw age. ^10^Be inheritance was estimated as the mean residual offset between GIA-corrected ^10^Be ages and lag-corrected AMS ^14^C ages at equivalent elevations (ridges A, E, and O), yielding a mean of 1049 a (1σ: 919 a; 95% CI: 0–2850 a); this value was subtracted from all ^10^Be ages prior to regression. The combined inheritance uncertainty ${{{\boldsymbol{\sigma }}}}_{{{\boldsymbol{inh}}}}=\sqrt{{{{\boldsymbol{\sigma }}}}_{{{\boldsymbol{\triangle }}}}^{{{\boldsymbol{2}}}}{{\boldsymbol{+}}}{{{\boldsymbol{\sigma }}}}_{{{\boldsymbol{lag}}}}^{{{\boldsymbol{2}}}}}$ was propagated in quadrature with individual ^10^Be measurement uncertainties.

A second-order polynomial was then fitted to the lag-corrected AMS ^14^C and inheritance-corrected ^10^Be ages as a function of ridge elevation using inverse-variance weighting. The uncertainties ${{{\boldsymbol{\sigma }}}}_{{{\boldsymbol{resid}}}}$ and ${{{\boldsymbol{\sigma }}}}_{{{\boldsymbol{lag}}}}$, derived from the regression coefficient covariance matrix, were propagated in quadrature to yield per-ridge regression age uncertainties. An independent geometric constraint was additionally derived by regressing ridge elevation directly against distance from the modern shoreline, assigned a conservative uncertainty of 800 a (1σ) to prevent it from dominating the posterior where isotopic constraints exist. For each ridge, the Bayesian fusion step computed the posterior formation age as the inverse-variance-weighted mean of up to three estimates: the regression-based age, the inheritance-corrected ^10^Be age (ridges A, E, and O only), and the distance-based auxiliary age. The posterior age and its uncertainty are given by:

1 $${{{\boldsymbol{t}}}}_{{{\boldsymbol{post}}}}=\frac{{\sum }_{{{\boldsymbol{i}}}}{{{\boldsymbol{w}}}}_{{{\boldsymbol{i}}}}{{{\boldsymbol{\mu }}}}_{{{\boldsymbol{i}}}}}{{\sum }_{{{\boldsymbol{i}}}}{{{\boldsymbol{w}}}}_{{{\boldsymbol{i}}}}},{{{\boldsymbol{\sigma }}}}_{{{\boldsymbol{post}}}}={\left({\sum }_{{{\boldsymbol{i}}}}{{{\boldsymbol{w}}}}_{{{\boldsymbol{i}}}}\right)}^{-{{\bf{1}}}/{{\bf{2}}}},{{{\boldsymbol{w}}}}_{{{\boldsymbol{i}}}}=\frac{{{\bf{1}}}}{{{{\boldsymbol{\sigma }}}}_{{{\boldsymbol{i}}}}^{{{\bf{2}}}}}$$

where ${{{\boldsymbol{\mu }}}}_{{{\boldsymbol{i}}}}$ and ${{{\boldsymbol{\sigma }}}}_{{{\boldsymbol{i}}}}$ are the age estimate and 1σ uncertainty of the i-th source, respectively. Stratigraphic bounds were then applied as hard constraints: in situ marine shell ages within ±1 m elevation served as upper age limits; penguin and seal remain ages within ±1 m served as lower limits. Where the fused age exceeded the shell-derived upper bound, it was capped at that value and the posterior uncertainty scaled by 0.8 to reflect the additional constraint. Where the fused age fell below the minimum age indicated by penguin or seal remains, it was raised to that lower bound with the posterior uncertainty unchanged.

To assess robustness, the full procedure was repeated for colonization lag values of 0, 500, 1000, and 1500 a, yielding mean inheritance estimates of ~2049, ~1549, ~1049, and ~549 a, respectively (1σ: ~919 a in all cases). The systematic decrease with increasing lag reflects the correction structure, but the central estimates remain of comparable magnitude across the plausible lag range, supporting the robustness of the preferred correction.

### Wave height calculations

Nandasena’s hydrodynamic equations^28^ were employed to estimate the wave heights required to initiate boulder transport on the beach ridges of Inexpressible Island. The equations, derived from the moment of hydrodynamic forces applied to the boulder (including drag, lift, inertia, buoyancy, and gravity), can be applied to the respective environment of a boulder: submerged, subaerial, or joint-bounded, providing estimations of the minimum flow velocity required to initiate transport. The specific formulae are provided in Supplementary Table 6.

In Noji’s experiment^74^, drag coefficient values ranged between 1.5 and 5, with most values used in past studies falling between 1.5 and 3. In this study, coefficients of drag, lift, and static friction were chosen as ${C}_{{{\rm{d}}}}=2$, ${C}_{{{\rm{l}}}}=0.178$, ${\mu }_{{{\rm{s}}}}=0.7$, respectively. The density of seawater was taken as $1.02{{\rm{g}}}/{{{\rm{cm}}}}^{3}$, and gravitational acceleration is 9.81 ${{\rm{m}}}/{{{\rm{s}}}}^{2}$. The beach slope was measured to be approximately 10°, and the boulders are granite with a density of 2.55 ${{\rm{g}}}/{{{\rm{cm}}}}^{3}$. Critical wave heights were calculated across the full size distribution (P5–P95) of boulders on each ridge for three transport modes: sliding, rolling/overturning, and saltation/lifting (Supplementary Dataset 3).

To apply these equations, nearshore wave conditions were first derived from deep-water parameters via wave transformation. Open-ocean wave observations from the 2017 PIPERS campaign provide the necessary boundary conditions. During a major wave event in late June, the mean SWH in the open Ross Sea reached ~6.3 m, corresponding to a deep-water wave height (${H}_{0}$) exceeding 7 m^42,43^. Wave periods of 6–10 s were recorded across events where SWH exceeded 4 m, consistent with WAVEWATCH III model outputs^75^. Based on these data, we adopt ${H}_{0}=7\,{{\rm{m}}}$ and ${T}_{0}=6\,{{\rm{s}}}$ as representative deep-water storm inputs for nearshore wave transformation at the western coast of Inexpressible Island. Using the deep-water wave length equation ${L}_{0}=\frac{g{T}^{2}}{2\pi }$, the resulting wave length is approximately 56 m. With a measured beach slope of *θ* = 10° (tan *θ* ≈ 0.176), the wave run-up height (${R}_{2\%}$) was estimated using the formula^76^:

2 $${R}_{2\%}=1.1\left[0.35\tan \theta \sqrt{\left({H}_{0}{L}_{0}\right)}+0.5\sqrt{\left({H}_{0}{L}_{0}\right)\left(0.563{\tan }^{2}\theta+0.004\right)}\right]$$

yielding a maximum run-up height (${R}_{2\%}$) of approximately 3.19 m. This exceeds the relief of the raised beaches (0.50–3.09 m), confirming that storm waves of this magnitude were capable of overtopping and reworking the beach surface.

To further determine whether wave breaking occurred in the nearshore zone, we analyzed the relationship between the wave height and water depth at the breaking point. The breaking wave height ${H}_{{{\rm{b}}}}$ was estimated using the empirical equation proposed by Sunamura and Horikawa^77^:

3 $$\frac{{H}_{{{\rm{b}}}}}{{H}_{0}}={\left(\tan \beta \right)}^{0.2}{\left(\frac{{H}_{0}}{{L}_{0}}\right)}^{-0.25}$$

where β represents the average slope of the nearshore profile from the shoreline to a depth of 20 m, here approximated as consistent with the measured beach slope. The corresponding water depth at the breaking point ${h}_{b}$ was estimated using the empirical relationship provided by Collins^78^:

4 $$\frac{{H}_{{{\rm{b}}}}}{{h}_{{{\rm{b}}}}}=0.72+5.6\tan \beta$$

The calculated wave height at the breaking point is 8.32 m, with a corresponding water depth of 4.88 m (${H}_{{{\rm{b}}}}/{h}_{{{\rm{b}}}}\approx 1.70$), indicating that waves in the nearshore zone of Inexpressible Island were already in a breaking regime, enhancing their capacity to mobilize large boulders.

Accordingly, the critical wave height required to initiate boulder transport can be estimated based on the critical flow velocity using the equation $u=\delta \sqrt{{gH}}$, where *H* is the wave height (in meters), and δ is a wave type parameter. Specifically, when a storm occurs and wave breaking is present, δ = 0.5; when a storm occurs without wave breaking, *H* is defined as the breaking wave height ${H}_{{{\rm{b}}}}$, and δ = 1; for tsunami conditions, δ = 2 (ref. ^22^). In this study, δ is set to 0.5, reflecting the assumption of wave breaking during storm events. Based on the boulder size, density, and elevation, we then calculated the wave period conditions necessary to initiate boulder motion using the following equation^79^:

5 $$T=\frac{2}{g}\left(\frac{{\rho }_{{{\rm{w}}}}}{{\rho }_{{{\rm{s}}}}-{\rho }_{{{\rm{w}}}}}\right)\left(\frac{{C}_{{{\rm{d}}}}}{\tan \theta }\right)\left(\frac{{h}_{{{\rm{clast}}}}}{{D}_{i}}\right)u$$

where ${h}_{{{\rm{clast}}}}$ is the vertical displacement of the boulder (ridge relief minus boulder short axis), and ${D}_{i}$ is the intermediate clast diameter.

## Supplementary information

Supplementary information

Description of Additional Supplementary File

Dataset 1

Dataset 2

Dataset 3

Dataset 4

Transparent Peer Review file

## Data Availability

All data generated in this study are provided in the Supplementary Information and Supplementary Datasets 1–3, and have been deposited in the Zenodo repository (https://doi.org/10.5281/zenodo.21231862). All third-party datasets used in this study are publicly available: Antarctic boundary data (NSIDC-0709) are available at https://nsidc.org/data/nsidc-0709/versions/2; circum-Antarctic thin sea-ice and fast-ice products are available at https://coas.ouc.edu.cn/pogoc/2024/1009/c31871a485693/page.htm; Landsat 8 satellite imagery is available at http://www.gscloud.cn; Google Earth imagery is available at https://earth.google.com. All remaining third-party datasets, including Talos Dome ice core records (ref. ^36^), IRD records from the Antarctic Polar Front (ref. ^53^), Laguna Pallcacocha sediment records (ref. ^58^), previously published AMS ¹⁴C ages (refs. ^7,8,39,40,80–82^), and GIA model predictions for ICE-6G (ref. ^18^), ICE-5G (ref. ^19^), and W12 (ref. ^17^.), are available from the original publications cited in the manuscript.

## Code availability

The MATLAB script used for Bayesian fusion chronology in this study is provided as Supplementary Dataset 4 and is also publicly available at Zenodo: https://doi.org/10.5281/zenodo.21231862.
