# Supplementary material for: Early-mid Holocene Ross Sea extreme wave heights reflect sea ice retreat and ocean-atmosphere interactions
Source: Nat Commun. 2026 Aug 3;17:9721. doi: 10.1038/s41467-026-76311-y (PMC13562617; doi:10.1038/s41467-026-76311-y)
Supplement: Supplementary file 1 [file 41467_2026_76311_MOESM1_ESM.pdf]

Supplementary Information

Early-Mid Holocene Ross Sea Extreme Wave Heights Reflect Sea Ice Retreat and Ocean-Atmosphere Interactions

Shuo Wang *et al.*

*Corresponding author. Email: nlwang@nwu.edu.cn; xbdzyz05@nwu.edu.cn

This file includes:

Supplementary Figures 1–4
Supplementary Tables 1–6 (embedded in this file)

Additionally:

Supplementary Dataset 1 is provided as an Excel file (detailed clast measurements).
Supplementary Dataset 2 is provided as an Excel file (compiled ^{14}C ages).
Supplementary Dataset 3 is provided as an Excel file (wave parameter calculations).
Supplementary Dataset 4 (ridge_age_bayesian_fusion.m) is provided as a MATLAB script implementing the Bayesian fusion chronology framework described in the Methods section.

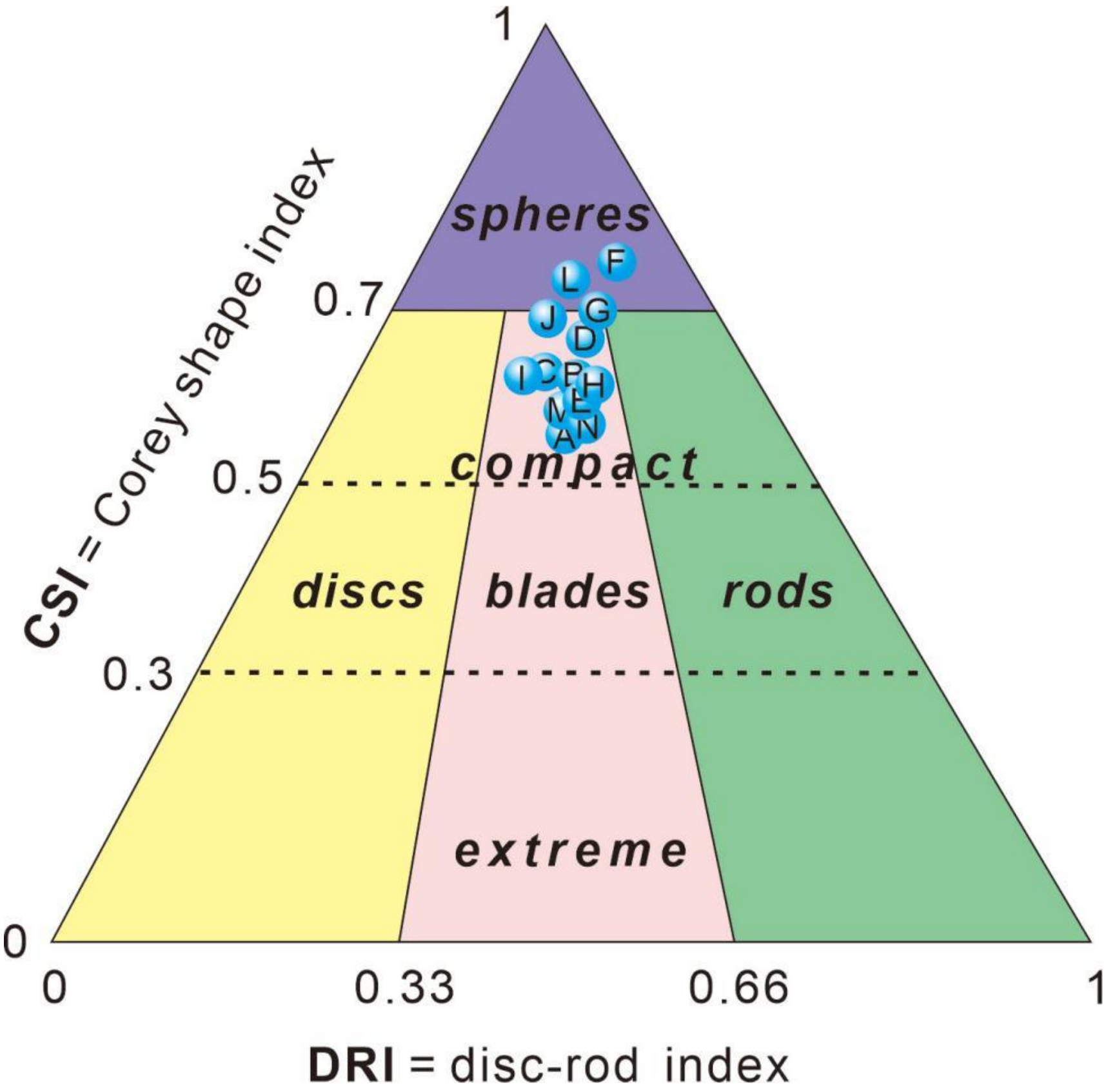

Supplementary Fig. 1.

The shape diagram: Corey Shape Index (CSI) versus Disc-Rod Index (DRI) on a triangular diagram. The three end-members of shape are spheres, discs, and rods.

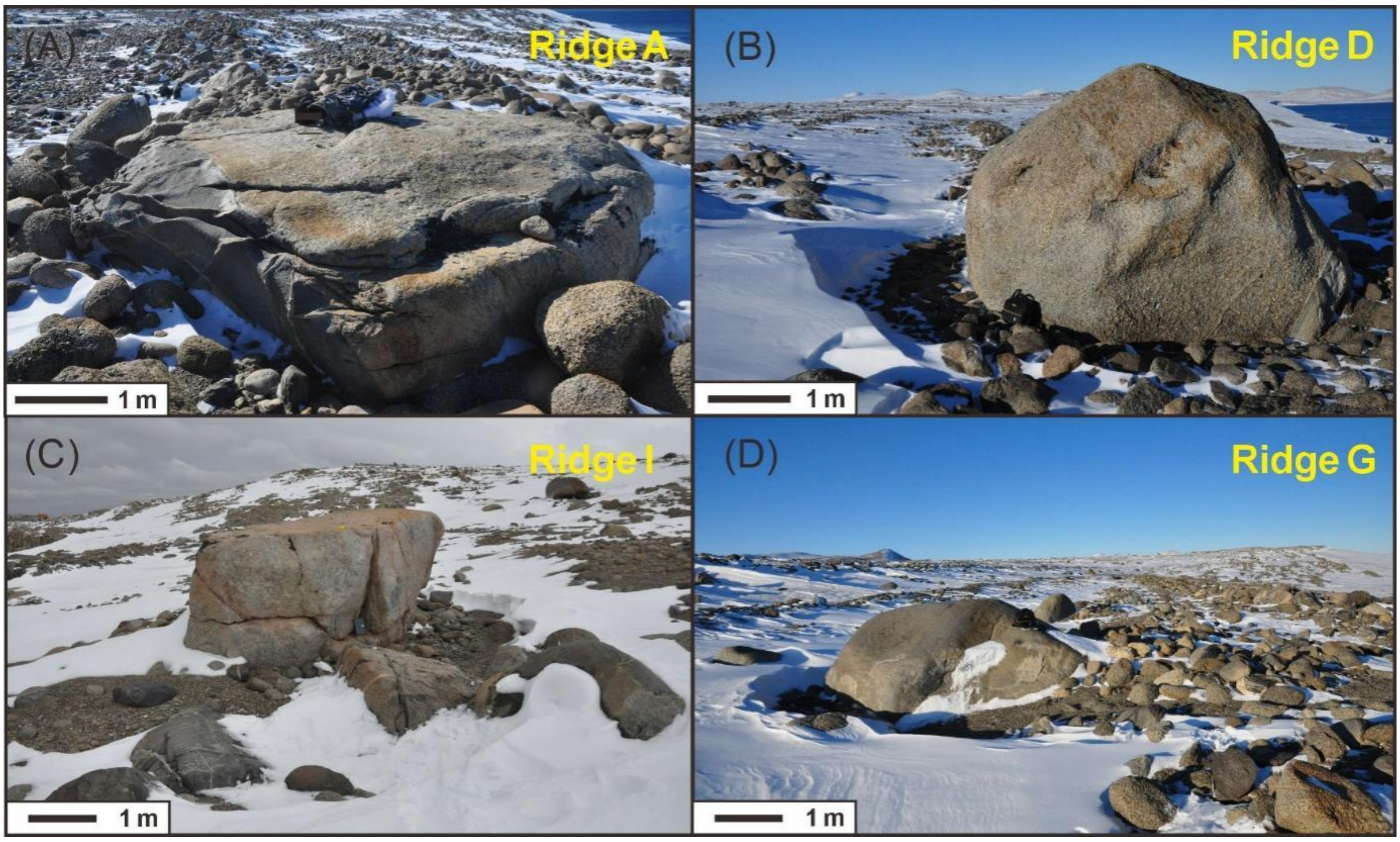

Supplementary Fig. 2.
Photographs of unusually large, angular, and poorly rounded boulders observed on raised beach ridges in Southernmost Bay of Inexpressible Island.

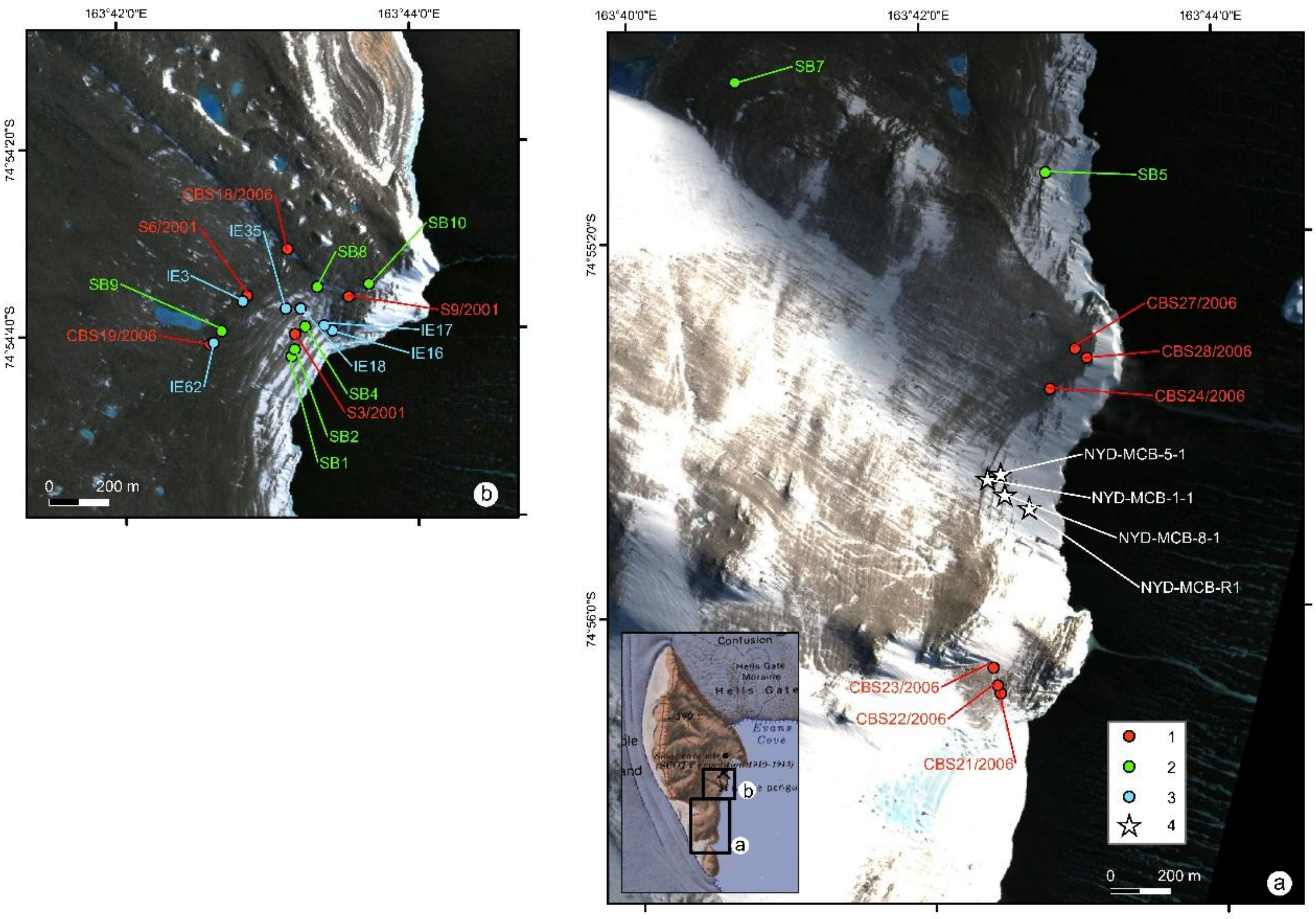

Supplementary Fig. 3.

Location of stratigraphic sections providing ^{10}Be and radiocarbon dates from "*Southernmost Bay*" (a) and "*South Bay*" (b) (see Supplementary Dataset 2). Red circles: sections from Baroni and Salvatore; green circles: sections from Baroni and Hall (2004); cyan circles: sections from Gao et al. (2022); white stars: ^{10}Be sampling sites (this study; see Supplementary Table 3). Base maps for (a) and (b) are based on a Quickbird multispectral image (University of Pisa). Inset location map is extracted from the U.S. Geological Survey Antarctica Reconnaissance Series, sheet "Mount Melbourne" (1:250,000).

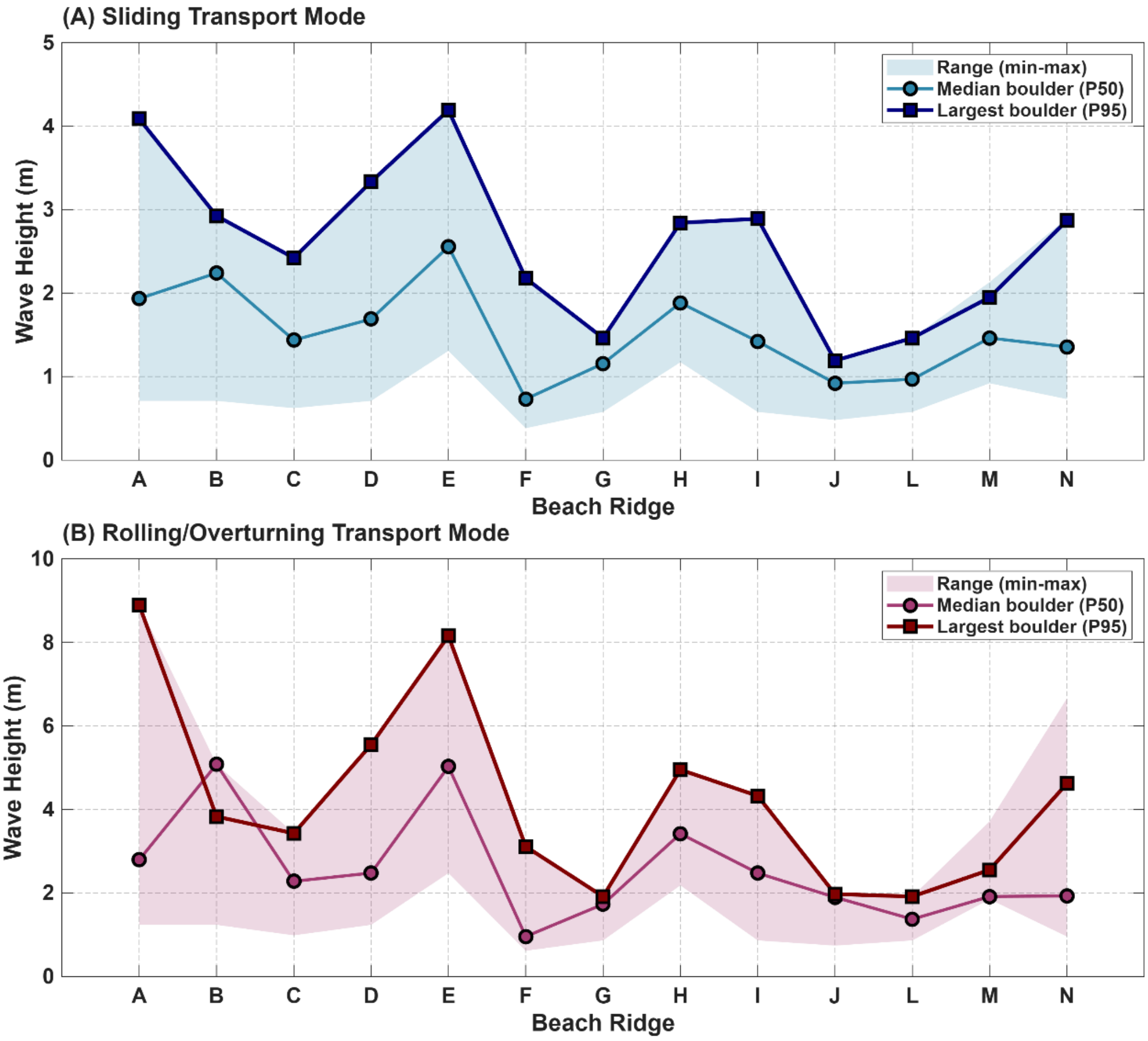

Supplementary Fig. 4.

Critical wave heights required to initiate boulder transport via sliding (A) and rolling/overturning (B) for each beach ridge (A–N) at the southernmost bay of Inexpressible Island. Circles and squares denote the median (P50) and largest (P95) boulder size classes, respectively, and shaded areas represent the full range (min–max) of wave heights across the P5–P95 size distribution. Beach ridges K and O are excluded due to snow cover during field measurement.

Supplementary Table 1.

Locations (longitude, latitude), beach ridge elevations (top, bottom, relief) and difference in distance in between adjacent beach ridge (beach ridge G was not measured due to field instrument failure).

Ridge		Elevation (m)	Relief (m)	Distance (m)	Longitude (°E)	Latitude (°S)
A	Top	28.59	3.09	-	163°42′22.330″	74°55′48.446″
	Bottom	25.50			163°42′22.168″	74°55′48.655″
B	Top	27.33	2.10	9.0	163°42′24.013″	74°55′48.101″
	Bottom	25.23			163°42′23.948″	74°55′48.128″
C	Top	25.94	0.92	10.0	163°42′25.424″	74°55′47.953″
	Bottom	25.02			163°42′25.639″	74°55′47.967″
D	Top	25.19	0.90	9.5	163°42′26.887″	74°55′47.650″
	Bottom	24.29			163°42′26.914″	74°55′47.609″
E	Top	25.15	2.81	5.5	163°42′27.559″	74°55′47.631″
	Bottom	22.34			163°42′27.746″	74°55′47.696″
F	Top	22.11	0.73	19.0	163°42′29.918″	74°55′47.619″
	Bottom	21.38			163°42′29.891″	74°55′47.628″
G	Top	-		10.0	-	-
	Bottom	-			-	-
H	Top	21.83	1.34	6.0	163°42′31.849″	74°55′47.545″
	Bottom	20.49			163°42′32.627″	74°55′47.579″
I	Top	20.51	1.20	13.5	163°42′33.685″	74°55′47.458″
	Bottom	19.31			163°42′34.204″	74°55′47.456″
J	Top	18.29	0.54	10.0	163°42′35.143″	74°55′47.462″
	Bottom	17.75			163°42′35.494″	74°55′47.413″
K	Top	17.92	0.50	7.5	163°42′35.267″	74°55′48.103″
	Bottom	17.42			163°42′35.503″	74°55′48.191″
L	Top	17.73	0.60	3.5	163°42′35.804″	74°55′48.156″
	Bottom	17.13			163°42′36.049″	74°55′48.233″
M	Top	18.21	1.72	8.5	163°42′35.675″	74°55′49.175″
	Bottom	16.49			163°42′36.331″	74°55′49.111″
N	Top	16.33	1.47	11.0	163°42′37.022″	74°55′49.519″
	Bottom	14.86			163°42′37.705″	74°55′49.602″
O	Top	14.17	0.78	15.0	163°42′38.926″	74°55′49.756″
	Bottom	13.39			163°42′39.208″	74°55′49.763″

Supplementary Table 2.

Average particle size, volume, and weight of boulders for each beach ridge (A–N) of the southernmost bay of Inexpressible Island (Beach ridges K and O were not measured due to snow cover). The smallest and largest boulders within each ridge are identified by volume.

Number of Samples	Measured dimension	Average	Smallest Boulder	Largest Boulder
A:65	Long (cm)	65.6	13.0	160.0
	Intermediate (cm)	45.6	12.0	130.0
	Short (cm)	28.8	10.0	40.0
	Volume (m^3)	0.139	0.002	0.832
	Weight (t)	0.220	0.004	2.122
B:50	Long (cm)	59.5	12.0	150.0
	Intermediate (cm)	42.9	10.0	70.0
	Short (cm)	30.0	8.0	50.0
	Volume (m^3)	0.109	0.001	0.525
	Weight (t)	0.279	0.002	1.339
C:49	Long (cm)	42.0	13.0	120.0
	Intermediate (cm)	32.4	12.0	100.0
	Short (cm)	22.4	8.0	60.0
	Volume (m^3)	0.049	0.001	0.720
	Weight (t)	0.124	0.003	1.836
D:50	Long (cm)	52.3	20.0	120.0
	Intermediate (cm)	38.7	13.0	60.0
	Short (cm)	28.8	8.0	40.0
	Volume (m^3)	0.085	0.002	0.288
	Weight (t)	0.217	0.005	0.734
E:68	Long (cm)	73.8	25.0	160.0
	Intermediate (cm)	51.2	20.0	100.0
	Short (cm)	35.7	10.0	90.0
	Volume (m^3)	0.197	0.005	1.440
	Weight (t)	0.503	0.013	3.672
F:50	Long (cm)	25.2	8.0	75.0
	Intermediate (cm)	18.9	6.0	45.0
	Short (cm)	16.6	5.0	40.0
	Volume (m^3)	0.015	<0.001	0.135
	Weight (t)	0.039	0.001	0.344
G:50	Long (cm)	30.4	15.0	55.0
	Intermediate (cm)	22.2	10.0	35.0
	Short (cm)	18.1	8.0	32.0
	Volume (m^3)	0.016	0.001	0.062
	Weight (t)	0.042	0.003	0.157
H:70	Long (cm)	62.2	20.0	110.0
	Intermediate (cm)	43.9	18.0	100.0

	Short (cm)	30.8	10.0	55.0
	Volume (m^3)	0.117	0.004	0.605
	Weight (t)	0.298	0.009	1.543
I:50	Long (cm)	44.4	10.0	180.0
	Intermediate (cm)	32.3	10.0	75.0
	Short (cm)	22.7	8.0	50.0
	Volume (m^3)	0.062	0.001	0.675
	Weight (t)	0.159	0.002	1.721
J:38	Long (cm)	21.2	10.0	40.0
	Intermediate (cm)	16.5	8.0	25.0
	Short (cm)	13.1	8.0	20.0
	Volume (m^3)	0.005	0.001	0.020
	Weight (t)	0.014	0.002	0.051
L:41	Long (cm)	26.4	10.0	45.0
	Intermediate (cm)	20.7	12.0	45.0
	Short (cm)	17.0	8.0	30.0
	Volume (m^3)	0.012	0.001	0.061
	Weight (t)	0.031	0.002	0.155
M:59	Long (cm)	48.2	18.0	90.0
	Intermediate (cm)	33.7	16.0	50.0
	Short (cm)	22.9	10.0	45.0
	Volume (m^3)	0.048	0.003	0.203
	Weight (t)	0.121	0.007	0.516
N:50	Long (cm)	53.1	20.0	105.0
	Intermediate (cm)	37.4	18.0	80.0
	Short (cm)	25.2	8.0	50.0
	Volume (m^3)	0.076	0.003	0.420
	Weight (t)	0.194	0.007	1.071

Supplementary Table 3.

Sampling information of the boulders in the beach ridges at Southernmost Bay on Inexpressible Island, Antarctica.

Location	Sample ID	Latitude (ºS)	Longitude (ºE)	Elevation (m asl)	Boulder size L/W/H (cm)	Thickness (cm)	Topographic shielding factor
Ridge A	NYD-MCB1-1	74.929501	163.706856	28.0	330/330/60	2.0	0.999901
Ridge E	NYD-MCB5-1	74.929702	163.707789	23.4	80/60/25	2.0	0.999913
Ridge H	NYD-MCB8-1	74.929948	163.708743	20.5	85/85/35	2.0	0.999904
Ridge O	NYD-MCB-R1	74.930201	163.711406	14.0	120/100/60	2.0	0.999914

Supplementary Table 4.

^{10}Be exposure ages of the boulders in the beach ridges at Southernmost Bay on Inexpressible Island, Antarctica.

Location	Sample ID	Quartz	^{9}Be carrier	Density	^{10}Be/^{9}Be	^{10}Be Concentration	Apparent exposure ages (1σ external errors)	GIA-corrected ages
		(g)	(g)	(g·cm^{-3})	(10^{-15})	10^{3} atoms·g^{-1}	(ka)	(ka)
Ridge A	NYD-MCB1-1	30.0760	0.2763	2.7	89.20 ± 10.35	50.80 ± 5.98	9.07 ± 1.20	9.21 ± 1.20
Ridge E	NYD-MCB5-1	30.1633	0.2736	2.7	87.60 ± 9.44	49.25 ± 5.40	8.83 ± 1.10	8.94 ± 1.10
Ridge H	NYD-MCB8-1	30.3168	0.2764	2.7	36.20 ± 6.97	20.20 ± 4.04	3.61 ± 0.75	3.62 ± 0.75
Ridge O	NYD-MCB-R1	30.1907	0.2746	2.7	51.00 ± 8.05	28.57 ± 4.61	5.22 ± 0.90	5.25 ± 0.90

Supplementary Table 5.

Bayesian fusion formation ages for raised beach ridges A–O at Southernmost Bay, Inexpressible Island, with contributing age sources and applied stratigraphic constraints.

Ridge	Distance from shore (m)	Elevation (m asl)	Formation age (a BP)	1σ (a)	Stratigraphic constraints	Age sources*
A	225.0	28.6	8043	522	Guano min: 4851 a BP	Reg, ^{10}Be, Dist
B	216.0	27.3	7860	555	Guano min: 4851 a BP	Reg, Dist
C	206.0	25.9	7667	555	-	Reg, Dist
D	196.5	25.2	7523	554	-	Reg, Dist
E	191.0	25.2	7523	517	-	Reg, ^{10}Be, Dist
F	172.0	22.1	7031	555	Guano min: 3834 a BP	Reg, Dist
G	162.0	-	6923	809	-	Dist (interpolated)
H	156.0	21.8	6858	555	Guano min: 3744 a BP	Reg, Dist
I	142.5	20.5	6604	555	-	Reg, Dist
J	132.5	18.3	6275	556	-	Reg, Dist
K	125.0	17.9	6164	556	-	Reg, Dist
L	121.5	17.7	6111	556	-	Reg, Dist
M	113.0	16.5	5887	556	Guano min: 365 a BP	Reg, Dist
N	102.0	16.3	5766	556	Guano min: 365 a BP	Reg, Dist
O	87.0	14.2	4960	408	Shell max: 4960 a BP	Reg, ^{10}Be, Dist

*Age sources: Reg = joint weighted regression; ^{10}Be = inheritance-corrected cosmogenic exposure age; Dist = distance-from-shoreline auxiliary constraint.

Supplementary Table 6.

Formula for calculating the critical flow velocity required to initiate boulder movement from Nandasena et al., (2011).

Transport Mode	Environment of the Boulder	
	Submerged Subaerial	Joint bounded
Sliding	$u^2 \geq \frac{2\left(\frac{\rho_s}{\rho_w}-1\right)gc(\mu_s\cos\theta+\sin\theta)}{C_d\left(\frac{c}{b}\right)+\mu_s C_l}$	/
Rolling/Overturning	$u^2 \geq \frac{2\left(\frac{\rho_s}{\rho_w}-1\right)gc\left(\cos\theta+\frac{c}{b}\sin\theta\right)}{C_d\left(\frac{c^2}{b^2}\right)+C_l}$	/
Saltation/lifting	$u^2 \geq \frac{2\left(\frac{\rho_s}{\rho_w}-1\right)gc\cos\theta}{C_l}$	$u^2 \geq \frac{2\left(\frac{\rho_s}{\rho_w}-1\right)gc(\cos\theta+\mu_s\sin\theta)}{C_l}$
