# Supplementary material for: Early-mid Holocene Ross Sea extreme wave heights reflect sea ice retreat and ocean-atmosphere interactions
Source: Nat Commun. 2026 Aug 3;17:9721. doi: 10.1038/s41467-026-76311-y (PMC13562617; doi:10.1038/s41467-026-76311-y)
Supplement: Supplementary file 2 [file 41467_2026_76311_MOESM2_ESM.pdf]

Description of the Additional Supplementary Files

Supplementary Dataset 1: Detailed measurements of all 690 individual boulders, including axis dimensions (long, intermediate, and short axes), volume, weight and Maximum Projection Sphericity (MPS), Oblate-Prolate Index (OPI), Corey Shape Index (CSI), and Disc-Rod Index (DRI).

Supplementary Dataset 2: Compilation of published radiocarbon (14C) ages from South Bay and Southernmost Bay of Inexpressible Island.

Supplementary Dataset 3: Wave velocity (u), wave height (H), and wave period (T) calculated using Nandasena et al. (2011) equations for three transport modes. Percentiles (P5-P95) represent the distribution of boulder sizes, reflecting the range of wave conditions during beach formation. P50 represents median conditions; P95 represents extreme events.

Supplementary Dataset 4: MATLAB script (ridge_age_bayesian_fusion.m) implementing the Bayesian fusion chronology framework described in the Methods section.
