# Supplementary material for: Early-mid Holocene Ross Sea extreme wave heights reflect sea ice retreat and ocean-atmosphere interactions
Source: Nat Commun. 2026 Aug 3;17:9721. doi: 10.1038/s41467-026-76311-y (PMC13562617; doi:10.1038/s41467-026-76311-y)
Supplement: Supplementary file 3 [file 41467_2026_76311_MOESM7_ESM.pdf]

nature portfolio

Peer Review File

Early-Mid Holocene Ross Sea Extreme Wave Heights Reflect Sea Ice Retreat and Ocean-Atmosphere Interactions

Corresponding Author: Professor Ninglian Wang

This file contains all reviewer reports in order by version, followed by all author rebuttals in order by version.

Version 0:

Reviewer comments:

Reviewer #1

(Remarks to the Author)
L1: The title is fitting for the contents, but not ideal for a wider and interdisciplinary readership.

L36ff: For populated coastlines, an increase in overall wave energy and in the frequency of extreme events matters for obvious reasons. Please work out (and briefly explain here), how this matters for Antarctica?

L44: Arguably, raised boulder beaches are only found at a few locations along the Antarctic coastlines.

L46ff: Isostatic uplift and relative sea level development are not that straight-forward close to ice shields and despite glacier retreat, local subsidence is not uncommon in both Greenland and Antarctica (e.g. Whitehouse et al. 2012, Geophys. J. Int.; Whitehouse et al. 2019, Nat. Comms.).

L50ff: You skip an important step here: Beach ridges are sediment-surplus features and form at locations, where mobile sediment is present and can be preserved (sediment input + accommodation). Due to their wide-spread occurrence in previously glaciated areas, it is a common misconception that a forced regression is a prerequisite for their formation. They do occur under RSL stability and even subsidence (see e.g. Sander et al. 2019 Polar Res., Hein et al. 2013 Sedimentology).

L59 (Fig. 1): The blue/dotted shape is not explained in the legend. It works well in the zoom level of map frame “a”, but is too coarse in frame “b”. Since the energy (for the formation of the beach ridges) comes from the east it is important to delineate the polynya and to show the extent of seasonal drift and fast ice. The scale bar does not work: the distance from 0-500 km is separated into three subsections, which gives an odd measure. Would it make sense to provide references to the dots/studies in frame “b”?

L60f: This reads a little inflated. Antarctic beach ridges have not been used anywhere near as much as Arctic beach ridges and the distribution of sites around Antarctica is too small to meaningfully inform GIA and ice sheet models.

L64f: The relationship between beach-ridge height and RSL is not straight forward – in most climates and sedimentary settings. The uncertainty is largest on energetic boulder beaches.

L72f: Please rewrite – meaning unclear: “Since raised beach elevation data may be affected by wave heights of 2-3 meters”. Do you mean runup?

L75-83: This is where you shine! Make sure to build the introduction more carefully towards your main argument and dataset. Several of the beach ridges aspects are too general.

L87-104: Could you provide more (quantitative) details on the polynya? How large is it (fetch!)? Is it open year-round or only seasonally ice-free? Do geological reconstructions exist as to the persistence of the polynya? Please separate info on the regional setting more from the methods and aims. The paragraph is very important as a transition from the very general introduction to the results.

L101f: These are results.

L110 (Fig. 2): Map “a” (label is not readable) is a little messy and it takes a while to understand what’s displayed. Could you find a clearer satellite image? Also, the data source is missing. Please use a rectangle to mark the location of map “b”. The color gradient is not explained in the legend (what is white/light gray?) and I would suggest to use more neutral colors for the land areas – please separate land and glacier.

L114: Please rewrite. It reads as if the ridges were 14-28 meters high.

L127: Photo “a” is not an aerial image (the shadow of the person taking it is visible). Could you add a scale to photos b-e?

L128: How were the boulders measured?

L129+L131: Use the same number of decimals.

L132: Do you have climate/wave data to explain what “conventional” hydrodynamic forcing is for that location?

L134: Not sure I understand. Is each ridge finer on the landward site or are the innermost ridges finer (and gradually become coarser towards the sea)?

L139: I disagree. If the source, lithology and transport pathway of the boulders is unclear, it is difficult to draw conclusions based on the shape alone. Transport and rounding could have happened long before marine processes started acting.

L143~: I agree that the size of the stones require substantial energy and that the shape of the ridge crests indicate the involvement of wave energy in the depositional processes. Since you had not mentioned it, I was just wondering: How do the modern beaches look (incl. the upper shoreface)? Would you expect any postdepositional changes? Cold, dry, katabatic winds could induce aeolian transport and remove fines.

L149: Are the ages in the same order as listed in the text (A, E, H, O). If yes, there is an age inversion and the youngest beach is ~5 kyr old.

L150: These 50 radiocarbon ages are crucial for your argument and need to be explained more in the main text – especially regarding their representativeness.

L155: I can not see how supraglacial transport should matter in the marine deposited ridges, but aeolian reworking, frost transport, or weathering might.

L166 (Fig. 4): I am a little surprised by the location of the 10Be age for ridge “O” – which appears (in Fig. 3) to be right on the modern shore. Where do all the other samples come from? Also, the text reads as if the ages are form your own previous work (which may be my own interpretation), but they are not. Please mark this very clearly incl. the fact that the date of publication was 20 and >30 years ago.
It could be useful to also plot the ages against distance from shore. How was the age of the beach ridges estimated? I can see no relationship with your 10Be ages?

L204: This is a surprisingly precise estimate of glacier retreat? Any chance there is a wrong unit here?

L211: For this you need to establish the precise elevation of the ridges and the relationship of that elevation with relative sea level (indicative meaning) and then establish the age of the deposits.

L235 (Fig. 235): The conceptual model bears striking resemblance with the model from Lindhorst & Schutter (2014, Geomorphology), including the wavy surface for the erosional unconformity. This needs to be marked clearly. The RSL curve by Baroni & Hall 2004 is based on a scatter of limiting ages and your adjusted curve probably fits well within the window of uncertainty. Where do the new details come from?

Reviewer #2

(Remarks to the Author)
It was a treat to read this manuscript, and most of the arguments are intriguing and the conclusions informative. However, I find the inferences of the wave amplitudes from which these wave heights are derived oversimplified and therefore spurious. Storm waves are random processes. The linear assumption between velocity and speed that is employed is a gross underestimation of the uncertainty that is generated by constructive and destructive wave-wave interaction that could result in the movement of boulders. I understand that the employed equations are simple, but other models of boulder transport have shown that nonlinearity of the wave and the interaction of the boulder with the moving fluid during the transport process are important considerations. Without the proper treatment of the nonlinearities of the transport process, the calculated values should not be trusted. A more sophisticated model would also allow for a more robust statistical analysis of the inverted wave heights and what they mean. Because of these flaws, I cannot recommend this manuscript for publication.

Reviewer #3

(Remarks to the Author)

Dear authors,
The manuscript by Wang et al., focus on the use of boulder beach ridges to reconstruct sea-level and extreme wave patterns from Mid-Holocene along the coast of Inexpressible Island (Antarctica).

The manuscript is well-written and provides informative figures that support the reasoning forwarded. The objectives of the manuscript are clear and are based on the use of raised boulder beaches to reconstruct storminess patterns along the Ross Sea. The manuscript is structured on a compination of field data (topographic measurements and particle size and shape measurements), age-estimation methodologies (cosmogenic and raiocarbon) and hydrodynamic modelling. This approach is sound and provides good basis for the discussion forwarded by the authors.

Overall, I have to commend the authors on their work. Nevertheless, the approach followed is classical and based on a single study area, even if it is in Antarctica. The innovative character of the work lies on the location studied. Having said this, conceptually and methodologically the approach made by the authors is robust and the interpretation seem well-supported by the data presented.

My main concerns are with the lack of geographical scope, with the height estimations, with the reliability of some of the dates obtained, the particle shape inferences, the particle transport equations used and with the storminess vs extreme events interpretation. I will detail some of my concerns below. Nevertheless, I would like to stress that my intention is to seek further clarifications and not to disregard or question the work and interpretations of the authors. I do believe they did an excellent work.

So, in detail:
1- Geographical scope - the fact that there is limited (in geographical area) evidences presented is a cause for some concern and ideally correlation with other locations need to be further stressed on the manuscript;

2- Height estimations - in one part of the manuscript you refer to have used DGPS-RTK measuments but to calculate the slope of the beach berm you refer to have used a hand-set GPS (typically with no RTK and with a very high associated height error). This is critical and should be clarified in the manuscript;

3- Age-estimation - you solely present 4 new cosmogenic dates and use 50 radiocarbon dates previously published. This might be insufficient especially when the 10Be dates show such discrepancies and deviations. Chronological accuracy is pivotal for this work and Figure 4 exposes the 10Be dates as outlyers. Therefore, further caution should be added on some of the chronological interpretations forwarded later on the manuscript;

4- Particle shape, size, hydrodynamic equations and genetic events - rounded particles, namemly gravel and boulders, tend to be a result of reccurrent action from waves. In fact, boulder ridges are mostly associated with the long-term succession of storm waves and not with single events. One aspect that I would like the authors to improve is to clarify if we are analysing gravel, bloks or boulders. Along the manuscript there is reference to different particle sizes and one wonders exactly what was studied. This has implications in the sediment transport formulas to be used. Nandasena's formulation is correct and commonly applied. By assuming rolling as the main transport mechanism you imply the smallest wave heights were responsible for transport against gravity...I have some concerns regarding this. Certainly some transport was by suspension and saltation and those values would significantly increase the wave height necessary for movement. This need to be much more discussed in the manuscript. Fiannly, I missed a section with the hydrodynamic regime (mean wave height, storm wave base, period, etc.)...and also a section of provenance of the boulders. You referred to them as granitic but can you establish the original source? Can you please provide some insight on this? This is also very important....to infer travel distances and to be certain of wave height necessary we need to have an idea were the boulders were sourced from. What was the minimum horizontal distance traveled? An the vertical? In my opinion this needs to be added to the manuscript to make the interpretations forwarded even more robust.

5- Storminess vs Extreme events - based on the point above, I consider that storminess and not single events are likely the cause of the boulder ridges described in the manuscript. This result should be even further stressed by the authors as it is the key point of this work.

Overall, a very nicely written manuscript that presents a very interesting case study. In my opinion the manuscript should be accepted for publication after minor changes are made.
Kind regards
Pedro Costa

Version 1:

Reviewer comments:

Reviewer #4

(Remarks to the Author)
I, first, want to commend the authors on the most comprehensive and thorough responses to reviewer comments that I have ever seen. This is the first version of the manuscript that I am reviewing and found the analytical methods appropriate, the results well presented, and the discussion convincing and backed by results. By combining beach ages, granulometry of beach clasts, wave modeling, and published proxy datasets, the authors present a compelling case for heightened

storminess in the early Holocene linked with decreased sea ice and initial deglaciation of the coastal environment.

A couple comments/questions that I have relate to the choice of using the second to largest clast to estimate wave energy and be careful to acknowledge that individual beaches could be formed by a single or multiple storm events. Why not use mean clast size or incorporate uncertainties by using the range of clast sizes?

Reviewer #5

(Remarks to the Author)
I enjoyed reading the revised manuscript and review/response, the paper is interesting and I think it is worth publishing. I have some comments on how it can be improved, with a focus on the 10Be exposure ages, specifically how they have been interpreted and applied:

1. The authors describe the 10Be ages as “effective” exposure ages, do they mean “apparent” exposure ages, assuming one period of continuous exposure and no erosion? If so, this should be made clearer in the text.

2. The authors state in their revised text (line 190 - 192) that:

“However, when the external uncertainties of both dating methods are taken into account, their age ranges show substantial overlap. This indicates that the apparent offset is within the expected bounds of analytical uncertainty, and both dates likely reflect the same event, without any fundamental contradiction.”

Given that we are comparing different dating methods and the overlap of external uncertainties is used to argue that the methods are dating the same event (and in agreement), I think these external uncertainties need to be included in Figure 4. At present, Figure 4 makes it seem like the 10Be ages overestimate the ages of the beaches (vs the 14C ages), as the authors state in the text (and was noted by another reviewer). The authors could argue that the 10Be ages are (at least in part) a little older than 14C ages here due to a small amount of nuclide inheritance due to, for example, 10Be being produced through shallow water, meaning boulders making up the beach may contain a prior signal to beach formation. This might not be how the authors are interpreting the 10Be ages (and they could disagree, of course), but it would be helpful if the paper had a little more info generally on how they have been interpreted. Another consideration on the 10Be ages is a correction for GIA - we know the beaches were formed at lower elevations and have since been uplifted by GIA, how would this impact the resulting exposure ages? Presumably the higher elevation, older ages would be impacted the most, as they have been uplifted the greatest extent (e.g. see Jones et al. (2019) https://doi.org/10.1016/j.quascirev.2019.03.012). Alternatively, if the authors are assuming no elevation change impact on exposure ages, this needs to be stated within the text (apologies if I have missed it!).

3. I think the description of the integrated age-elevation framework that the authors are using needs i) a bit more detail in the main text, and ii) a new, very detailed section in the methods.

To demonstrate why I think these are needed, I go through the following example. The authors state on lines 193-194 “For ridges with direct ^{10}Be or ^{14}C ages at the corresponding elevations, these were directly applied”. The age of formation for Ridge A is “ between 8400 and 7300 yrs BP”, however, Ridge A has a 10Be age of 9.07 ± 1.20 ka, so why is the age of formation not ~9 ka? With Ridge A, are the authors using the 10Be age and 14C ages to get to this age of 8400 - 7300 yrs BP? If so, this is unclear as written. If this is the case, how are the two constraints used and weighted? How does this vary for each beach ridge? I could be missing something here, but I think the paper is lacking a detailed section on this important part of the study.

4. Lastly, I think some info on the new 10Be ages is missing from the Methods section/supplement. In order to reproduce the 10Be concentrations (and ages), we need to know the concentration of the 9Be carrier, as well as the 10Be concentration (or ratio) of procedural blank(s) and how the blank correction was applied. E.g. were the four samples part of a larger batch of samples? How many procedural blanks were used to correct the sample 10Be concentrations, e.g. one blank for a larger batch of samples? Multiple blanks from one batch with a mean blank correction applied? Or some kind of running average procedural blank?

Reviewer #6

(Remarks to the Author)
review of : Modelled Extreme Wave Heights in the Ross Sea during the Early to Mid- Holocene Reflect Sea Ice Retreat and Ocean–Atmosphere Interactions by Shuo Wang et al.

I have read the manuscript following the first round of reviews and corrections and I would like to commend the authoring team for their work and effort. The manuscript is, in my option greatly improved.
Below are my my comments in addition to the more detailed ones embedded in the Pdf file:

1) I believe that the approach of reconstructing paleo wave heights undertaken here is reasonable, especially after the more comprehensive workup presented and , in my opinion, addressed the issues raised by Reviewer 1
2) Chronological control- I struggle to easily correlate dated sites with the beach ridges. This needs to be clearly presented on a figure where dates are linked to field photographs. I suggest adding this information to Figure 3A. This way the reader

can intuitively understand if- where there are any age reversals and how do they influence interpretations:
3) Age uncertainties. Authors state that ' external uncertainties' for different dates from the similar locations overlap therefore the dates are statistically representing the same 'event' whereas the +/- ranges mentioned in the text do not seem to overlap at all. This needs to be clearly addressed as I fund it confusing at the very least.
3) References to till outcrops or local bedrock source are made in order to explain clast lithology and morphology but no information about the two formations are presented- please provide this information to support the claims.
4) Authors address previous reviewers comments by comprehensively presenting modern measurements of wave heights in the region. From what I understand the modern waved heights reach similar amplitudes to the ones postulated to had been responsible for the movement of the largest clasts. If correct, it is making the claim of 'Increased storminess between 5-8ka BP' questionable as a very short met-ocean measurement campaign already provided results that indicate that the storms of such magnitude can be considered common at present. This is perhaps my main criticism that needs clarification because the whole research hypothesis hinges on that result alone. Furthermore- The authors present a photograph of the modern beach ridge affected by storm processes but it does not appear to be included in the analysis which is a shame because it could serve as a validation/calibration point for all calculations given that the present day wave heights are known. Such approach would strengthen the manuscript a lot.
My outstanding questions are:

How can increased storminess in the past be resolved if present day waves are similar?
How is the storms frequency related to the beach ridge formation and can it be informed from the clast size and the fact of preservation of past beach ridges alone?
Does the fact that a ridge is preserved mean that there was a major stormy season/s and storms were frequent or does it in fact mean that there was a period of quiescence in storms after a stormier season that lasted long enough for the GIA to uplift the area beyond the limit of the next major storm cycle?

These aspects should be at the very least addressed in the discussion.

I would welcome the

Version 2:

Reviewer comments:

Reviewer #4

(Remarks to the Author)
Regarding my comment on the choice of the second to largest clast, the updated analysis that represents the full distribution of clasts is much improved. I am very happy with how the authors have approached that issue, as well as their acknowledgement of the beaches being formed by multiple storm events rather than single events. I have no further comments or suggestions to offer.

(Remarks on code availability)

Reviewer #5

(Remarks to the Author)
I commend the authors for their comprehensive response to my own comments and those of other reviewers, too. The manuscript is undoubtedly improved and, from my own perspective, it seems ready for publication.

(Remarks on code availability)
I'm not a MATLAB expert, but I can confirm that the code runs and generates one of the figures used in the manuscript, so it appears to function as intended.

Reviewer #6

(Remarks to the Author)
I'm pleased to say that, following the last round of reviews most of my major concerns were comprehensively addressed and the manuscript itself is improved. It is now clearly demonstrated that the modern-day wave conditions are substantially smaller than ones in the past.

The figures now link much better to the results and it is possible to easily follow the reasoning.
I have one last comment regarding the following statement:'Sample MCB8-1 from ridge H was excluded from this estimate, as it yields an anomalously young apparent age (3.61 ± 0.75 ka) relative to both the local 14C chronology and the other 10Be samples, likely reflecting post-depositional surface disturbance'

Whereas it is likely that the sample was disturbed it is important, in my view, to highlight that the disturbance mechanism here is unknown and might have been here, among other processes, a storm activity washing over lower beach ridges (H-O)

and disturbing this particular one. This in consequence yielded a younger age. other disturbance mechanism are also possible.

(Remarks on code availability)

Open Access This Peer Review File is licensed under a Creative Commons Attribution 4.0 International License, which permits use, sharing, adaptation, distribution and reproduction in any medium or format, as long as you give appropriate credit to the original author(s) and the source, provide a link to the Creative Commons license, and indicate if changes were made.
In cases where reviewers are anonymous, credit should be given to 'Anonymous Referee' and the source.
The images or other third party material in this Peer Review File are included in the article's Creative Commons license, unless indicated otherwise in a credit line to the material. If material is not included in the article's Creative Commons license and your intended use is not permitted by statutory regulation or exceeds the permitted use, you will need to obtain permission directly from the copyright holder.
To view a copy of this license, visit https://creativecommons.org/licenses/by/4.0/

Summary of Changes

The authors would like to thank the senior editor, and the reviewers for their constructive comments and kind support. We provide below a detailed account on the changes that we have made in response to the comments that the reviewers have raised. We have marked the corresponding changes in the revised version in *red* color.

Response to Senior Editor:

Author's reply:

We sincerely thank you for the opportunity to consider our manuscript and for sending it back for peer review. We are very grateful for this chance and have carefully revised the manuscript in response to the reviewers' comments. In particular, we have revised the title and introduction to broaden the interdisciplinary appeal of the study and better highlight the significance of Antarctic extreme wave events, clarified the geological context, regional setting, and data representativeness with new details on the Terra Nova Bay Polynya and the spatial coverage of raised boulder beaches, and enhanced methodological descriptions and figure clarity by addressing issues related to boulder measurements, elevation data, chronological datasets, and hydrodynamic modeling.

Furthermore, we have expanded the discussion of uncertainties, including those associated with dating, boulder transport, and wave reconstruction, to ensure a balanced interpretation of our results. In addition, we have added two co-authors, Prof. Carlo Baroni and Dr. Maria Cristina Salvatore, to strengthen the verification of radiocarbon data and improve the robustness of our chronological framework.

Finally, we have provided a detailed point-by-point response to each reviewer comment, and a revised manuscript with tracked changes is included for the reviewers' consideration. We hope that these revisions adequately address the reviewers' concerns and enhance the clarity, rigor, and overall quality of our work. We greatly appreciate your time and consideration in reviewing our clarifications.

Response to Reviewer #1:

L1: The title is fitting for the contents, but not ideal for a wider and interdisciplinary readership.

Author's reply:

We sincerely thank the reviewer for this valuable suggestion. Following the reviewer's advice, we have revised the title to broaden its appeal and highlight the interdisciplinary significance of our study. The new title, "Modeled Extreme Wave Heights in the Ross Sea during the Early to Mid-Holocene Reflect Sea Ice Retreat and Ocean–Atmosphere Interactions," emphasizes both the modeling aspect and the broader climatic and oceanographic implications, making it more accessible to a wider readership.

Revision:

Modeled Extreme Wave Heights in the Ross Sea during the Early to Mid-Holocene Reflect Sea Ice Retreat and Ocean–Atmosphere Interactions

L36ff: For populated coastlines, an increase in overall wave energy and in the frequency of extreme events matters for obvious reasons. Please work out (and briefly explain here), how this matters for Antarctica?

Author's reply:

We sincerely thank the reviewer for this insightful comment. We have revised the text to clarify the significance of wave activity for the Antarctic environment. The revised sentences (Lines 40–41) now explain that intensifying wave activity can threaten Antarctic coastal stability by enhancing erosion, accelerating sea-ice breakup, and increasing the risk of ice-shelf collapse, with potential implications for glacier discharge and sea-level rise. This addition highlights the broader environmental relevance of Antarctic wave processes in a warming world.

Revision:

… including intensified erosion, accelerated sea-ice breakup, and enhanced ice-shelf instability, all of which can influence glacier discharge and sea-level rise[1,2]. …

L44: Arguably, raised boulder beaches are only found at a few locations along the Antarctic coastlines.

Author's reply:

We appreciate the reviewer's helpful comment. We agree that raised boulder beaches are not widely distributed across Antarctica. To clarify this point, we have revised the sentence to better reflect their limited occurrence (Lines 48–50).

Revision:

… Raised boulder beaches have been identified along several Antarctic coastlines (Fig. 1), predominantly in the Ross Sea and the Antarctic Peninsula, with fewer occurrences in East and West Antarctica[6-13] …

L46ff: Isostatic uplift and relative sea level development are not that straight-forward close to ice shields and despite glacier retreat, local subsidence is not uncommon in both Greenland and Antarctica (e.g. Whitehouse et al. 2012, Geophys. J. Int.; Whitehouse et al. 2019, Nat. Comms.).

L50ff: You skip an important step here: Beach ridges are sediment-surplus features and form at locations, where mobile sediment is present and can be preserved (sediment input + accommodation). Due to their wide-spread occurrence in previously glaciated areas, it is a common misconception that a forced regression is a prerequisite for their formation. They do occur under RSL stability and even subsidence (see e.g. Sander et al. 2019 Polar Res., Hein et al. 2013 Sedimentology).

Author's reply:

We thank the reviewer for these valuable insights. We fully agree that the formation of raised beaches is not solely controlled by glacio-isostatic rebound or forced regression, but instead reflects the combined influence of relative sea-level fluctuations, sediment dynamics, and episodic high-energy wave activity. In the revised text, we chose to emphasize the wave-related processes shaping these features to maintain a clear focus on the study's main objective—quantifying the intensity of extreme wave events in Antarctica (Lines 50–51; 61–62).

Revision:

…Existing evidence indicates that periods of intensified beach formation often coincided with reduced sea-ice extent and enhanced wave activity …

…However, using raised beaches for RSL reconstruction is challenging because beach elevations are influenced not only by vertical land motion but also by hydrodynamic processes. …

L59 (Fig. 1): The blue/dotted shape is not explained in the legend. It works well in the zoom level of map frame "a", but is too coarse in frame "b". Since the energy (for the formation of the beach ridges) comes from the east it is important to delineate the polynya and to show the extent of seasonal drift and fast ice. The scale bar does not work: the distance from 0-500 km is separated into three subsections, which gives an odd measure. Would it make sense to provide references to the dots/studies in frame "b"?

Author's reply:

We thank the reviewer for the valuable comments and have carefully revised Fig. 1. The blue/dotted shape is now explained in the legend to indicate ice-shelf boundaries. Frame (B) has been updated to display a higher-resolution view. We have also added the delineation of the Terra Nova Bay Polynya and the extents of seasonal thin ice and fast ice. The scale bar has been corrected for proper representation, and the annotation of the study area in frame (B) has been removed.

Revision:

Fig. 1: Antarctic Holocene raised-beach landforms and their environmental context. (A) Distribution of Holocene raised beaches across Antarctica, with the oldest reported ages indicated by colored circles (locations and radiocarbon ages from ref[6].). Antarctic coastlines and ice-shelf boundaries are from the MEaSUREs Antarctic Boundaries for IPY 2007–2009 from Satellite Radar, Version 2 (NSIDC-0709; https://nsidc.org/data/nsidc-0709/versions/2). The maps

also show circum-Antarctic thin sea-ice thickness (0–0.2 m) as examples for the cold season (23 April 2020) and the warm season (23 January 2020); these layers are taken from the publicly released daily thin-ice products produced following the method of ref[84] and based on AMSR-E/2 L1R brightness temperatures and NSIDC NT2 sea-ice concentration data (source: https://coas.ouc.edu.cn/pogoc/2024/1009/c31871a485693/page.htm). Antarctic fast-ice extent is from the 2020 fast-ice dataset provided at the same source. (B) Enlarged view of the study area, showing the Terra Nova Bay Polynya region, with the red and orange shaded areas indicating the zone of >60% occurrence frequency during the freezing period (2003–2011) as reported by ref[84].

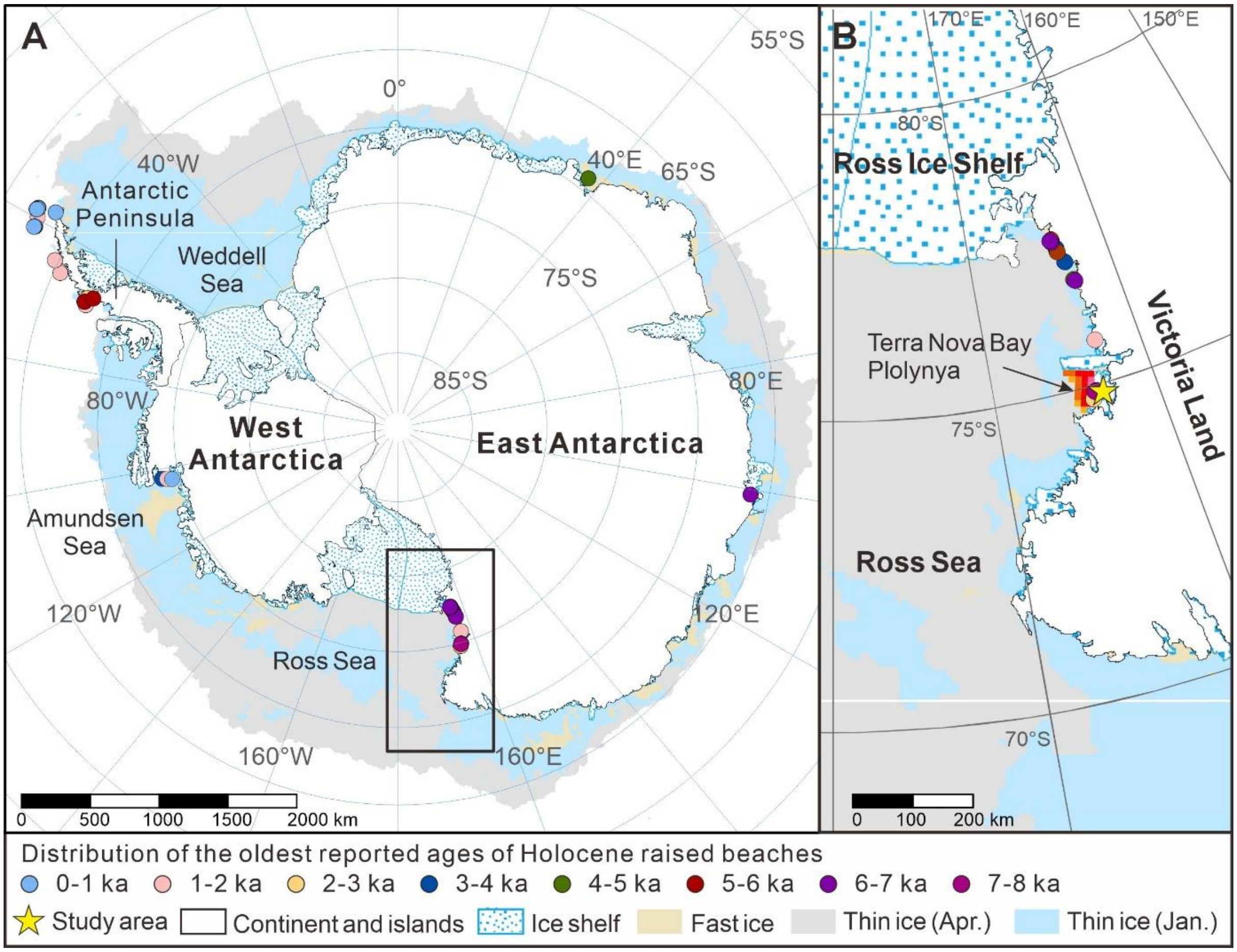

L60f: This reads a little inflated. Antarctic beach ridges have not been used anywhere near as much as Arctic beach ridges and the distribution of sites around Antarctica is too small to meaningfully inform GIA and ice sheet models.

Author's reply:

We thank the reviewer for this valuable comment and fully agree that describing Antarctic raised beaches as being "extensively used" for Holocene RSL reconstructions was an overstatement. Accordingly, We have revised the sentence to provide a more accurate description, acknowledging that, although sparse, these data still provide valuable constraints. We also added relevant references to clarify that existing GIA models, such as the W12 model (Whitehouse et al., 2012) and the ICE-6G model (Argus et al., 2014), incorporate RSL data from 14 and 12 Antarctic coastal sites, respectively, which are primarily based on material from raised beaches (Lines 59–61).

Revision:

…

Antarctic raised beaches have also served as key archives for Holocene relative sea-level (RSL) reconstructions[7-10], providing essential constraints on glacial isostatic adjustment (GIA) and ice-sheet models[13,18,19]. …

L64f: The relationship between beach-ridge height and RSL is not straight forward – in most climates and sedimentary settings. The uncertainty is largest on energetic boulder beaches.

Author's reply:

We thank the reviewer for this insightful comment and fully agree. In the revised manuscript, we have clarified that raised-beach elevations are influenced not only by vertical land motion but also by hydrodynamic processes, including wave run-up, which can introduce substantial uncertainty in RSL reconstructions, especially on energetic boulder beaches (Lines 61–64).

Revision:

…However, using raised beaches for RSL reconstruction is challenging because beach elevations are influenced not only by vertical land motion but also by hydrodynamic processes. Wave run-up of several meters can elevate beach deposits above still-water level[7,12], introducing uncertainty into RSL estimates and potentially obscuring the effects of extreme events.…

L72f: Please rewrite – meaning unclear: "Since raised beach elevation data may be affected by wave heights of 2-3 meters". Do you mean runup?

Author's reply:

We thank the reviewer for this helpful comment. We specified that the previously mentioned 2–3 m heights refer to wave run-up (Lines 62–63).

Revision:

…Wave run-up of several meters can elevate beach deposits above still-water level[7,12], introducing uncertainty into RSL estimates and potentially obscuring the effects of extreme events. …

L75-83: This is where you shine! Make sure to build the introduction more carefully towards your main argument and dataset. Several of the beach ridges aspects are too general.

Author's reply:

We thank the reviewer for this constructive suggestion. We fully agree that the introduction should be more focused on the main research question and dataset. In the revised version, we have carefully restructured the introduction to better guide readers toward the study's central objectives and the Inexpressible Island dataset. Specifically, the previous general statements about the distribution and origin of raised beaches across Antarctica have been condensed and replaced with a more focused discussion linking beach development directly to wave energy and storm activity (Lines 50–51). We also

added a short review summarizing existing studies on the relationship between Antarctic raised-beach formation and wave activity, to place our work within the broader regional context and highlight its representativeness (Lines 51–58).

In addition, we have added a clearer rationale connecting hydrodynamic influences and wave run-up with uncertainties in RSL reconstruction, emphasizing the importance of quantifying wave energy for understanding Antarctic coastal evolution (Lines 61–64, 69–72). We also incorporated more quantitative details on the Terra Nova Bay polynya, including recent observations and simulations of local wave conditions, to strengthen the transition toward our dataset and research objectives (Lines 88–93). We believe these revisions more clearly demonstrate why the raised boulder beaches of Inexpressible Island provide an ideal natural archive for reconstructing Holocene extreme wave events in Antarctica.

Revision:

…Existing evidence indicates that periods of intensified beach formation often coincided with reduced sea-ice extent and enhanced wave activity …

…For instance, granulometric records from Calmette Bay show increased beach development at approximately 1–2 and 5–6 ka, accompanied by more rounded clasts that reflect intensified wave reworking[6]. Similar observations from Anchorage Island and Rothera Station indicate heightened wave energy between 2.2 and 3.5 ka[9], while records from Horseshoe Island suggest enhanced wave activity and reduced summer sea ice between 2 and 6 ka[14]. Comparable relationships between diminished sea-ice cover and increased beach formation have also been reported from other high-latitude coasts, including the Varanger Peninsula in Arctic Norway, the Kola Peninsula in northwest Russia, and Lowther Island in the Canadian Arctic[15-17].

…

…However, using raised beaches for RSL reconstruction is challenging because beach elevations are influenced not only by vertical land motion but also by hydrodynamic processes. Wave run-up of several meters can elevate beach deposits above still-water level[7,12], introducing uncertainty into RSL estimates and potentially obscuring the effects of extreme events.…

… These discrepancies highlight the importance of distinguishing geomorphic and hydrodynamic influences when interpreting raised-beach elevations, and quantifying the wave energy responsible for their formation is therefore essential for understanding Antarctic coastal evolution.

…

… The TNBP always connects to the open sea directly in summer, with its area peaking at about 5000 km^2, while during the cold season it remains partially open, with a mean area of about 2361 km^2 (Fig. 2)[33,34]. These conditions enable the generation of local waves reaching 2–3 m in height even when most of the Ross Sea remains ice-covered, and allow the intrusion of Southern Ocean swells during summer when regional sea ice retreats[35,36]…

L87-104: Could you provide more (quantitative) details on the polynya? How large is it (fetch!)? Is it open year-round or only seasonally ice-free? Do geological reconstructions exist as to the persistence of the polynya? Please separate info on the regional setting more from the methods and aims. The paragraph is very important as a transition from the very general introduction to the results.

Author's reply:

We thank the reviewer for this valuable suggestion. We fully agree that a more quantitative and contextual description of the Terra Nova Bay Polynya (TNBP) would improve the transition from the general introduction to the study's regional focus. In the revised version, we have added specific information on the seasonal variability and extent of the TNBP, noting that it connects directly to the open sea in summer with an area of about 5000 km², and remains partially open during the cold season with a mean area of approximately 2361 km² (Lines 88–90). We also included recent observational and modeling evidence indicating that local waves of 2–3 m can develop even when most of the Ross Sea remains ice-covered, and that Southern Ocean swells can intrude during summer when regional sea ice retreats (Lines 90–93). Furthermore, we expanded the geological context by adding records of marine diatoms, sediments, and biological indicators (e.g., Adélie penguin colonies and elephant seal remains), which collectively demonstrate the persistence of the TNBP and open-water conditions since the mid-Holocene (Lines 93–100). These additions help to clearly separate the regional setting from the study aims and strengthen the logical transition to the following sections.

Revision:

… The TNBP always connects to the open sea directly in summer, with its area peaking at about 5000 km², while during the cold season it remains partially open, with a mean area of about 2361 km² [33,34]. These conditions enable the generation of local waves reaching 2–3 m in height even when most of the Ross Sea remains ice-covered, and allow the intrusion of Southern Ocean swells during summer when regional sea ice retreats[35,36]. Geological and paleoenvironmental evidence indicates that the TNBP has persisted for several millennia, maintaining open-water conditions that shaped coastal sediment dynamics[37-40]. Marine diatom and sediment records reveal millennial-scale variability in polynya efficiency and coastal sea-ice extent since the mid-Holocene[37]. Geomorphic and biological indicators, such as wave-formed raised beaches[8,38,39], Adélie penguin colonies[41], and elephant seal remains along the Victoria Land coast[40], further suggest the long-term persistence of open-water conditions in Terra Nova Bay.. …

L101f: These are results.

Author's reply:

We thank the reviewer for this helpful comment. We agree that the previous text included statements that read more like results rather than study objectives. In the revised version, we have rewritten this paragraph to clearly present our research aims and methodological framework instead of outcomes. The new text (Lines 105–111) now explicitly outlines the study's goals of quantitatively reconstructing the intensity and timing of Holocene extreme wave events, while describing the integration of field measurements, hydrodynamic modeling, and chronological analysis as part of the methodological approach. This adjustment helps ensure that the introduction concludes with a concise statement of objectives rather than a summary of results.

Revision:

… Specifically, boulder dimensions (length, width, and height) are used to estimate the wave heights and flow velocities required to initiate boulder transport under various hydrodynamic conditions. Radiocarbon (AMS ^{14}C) and in situ cosmogenic nuclide (^{10}Be) dating are applied to constrain the formation ages of these raised beaches and the timing of past

extreme wave events. By correlating reconstructed wave energy with post-glacial climate and relative sea-level variability, this work seeks to reveal how Antarctic coastal systems have responded to Holocene environmental changes and to provide new insights into the frequency and drivers of extreme wave activity.

L110 (Fig. 2): Map "a" (label is not readable) is a little messy and it takes a while to understand what's displayed. Could you find a clearer satellite image? Also, the data source is missing. Please use a rectangle to mark the location of map "b". The color gradient is not explained in the legend (what is white/light gray?) and I would suggest to use more neutral colors for the land areas – please separate land and glacier.

Author's reply:

We thank the reviewer for the helpful comments. In response, Figure 2 has been carefully revised. For frame (A), we selected a clearer Landsat 8 image with improved readability of labels and annotation. The data source is now explicitly provided (Landsat 8, 14 February 2023; http://www.gscloud.cn). A red rectangle is used to indicate the location of frame (B). Frame (B) has been replaced with a Google Earth image, which better displays the land and ice-shelf extent of Inexpressible Island, as well as the sampling locations. This revision improves the clarity of the spatial distribution of both previously published ^{14}C dating sites and newly collected ^{10}Be samples, all of which are situated on raised beaches.

Revision:

Fig. 2: Study area and sampling locations on Inexpressible Island, Antarctica. (A) satellite image of the Terra Nova Bay region, with nearby glaciers labeled in dark blue capital letters. The study area on Inexpressible Island is indicated by a red rectangle. Satellite imagery is from Landsat 8, 14 February 2023 (source: http://www.gscloud.cn). (B) Google Earth image showing the locations of collected samples on raised beaches across South Bay and Southernmost Bay of Inexpressible Island. Yellow circles indicate previously published radiocarbon (^{14}C) dating sites (see Supplementary Fig. 4 and Table 6), and green diamonds indicate the locations of newly collected ^{10}Be exposure age samples.

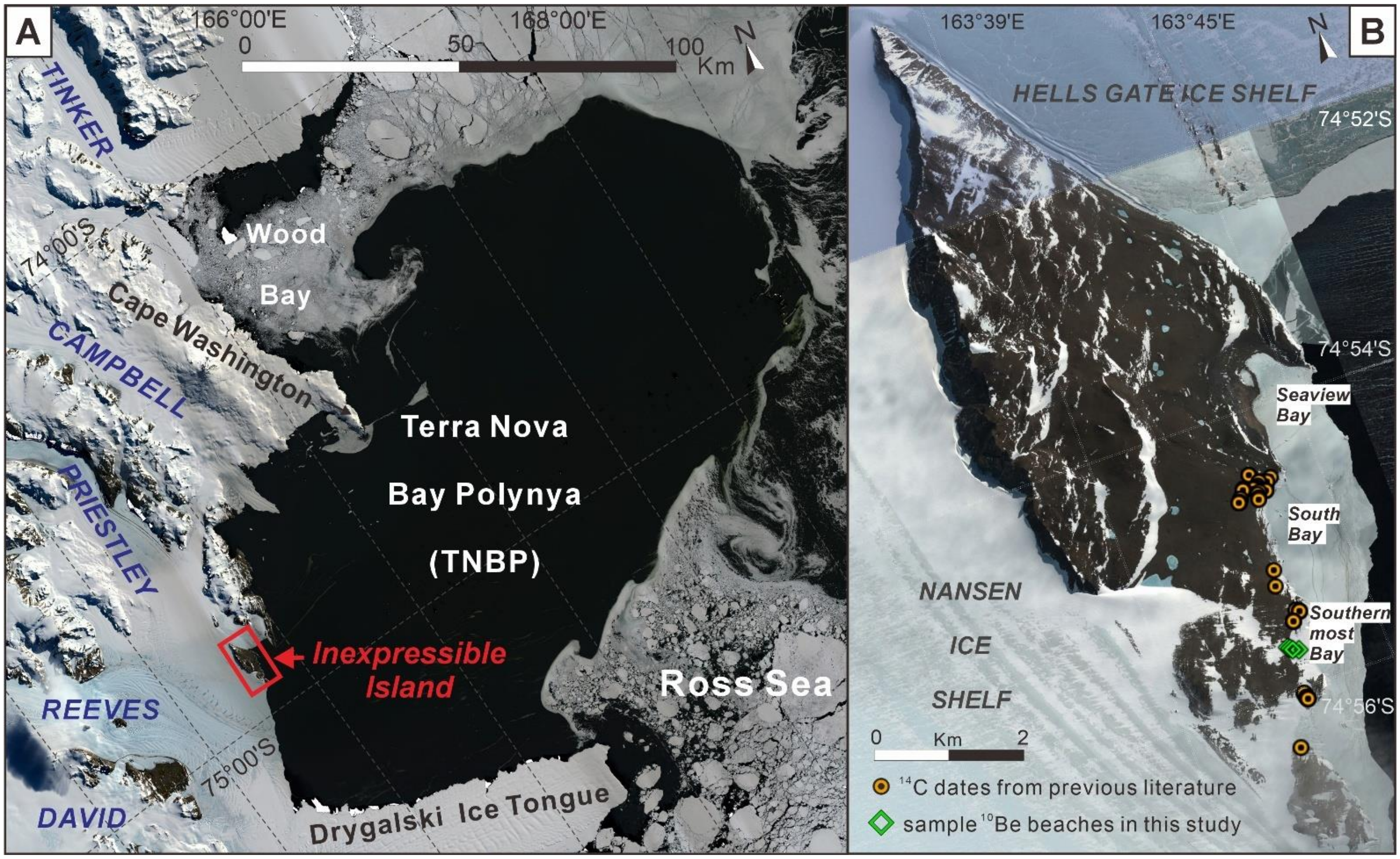

L114: Please rewrite. It reads as if the ridges were 14-28 meters high.

Author's reply:

We thank the reviewer for this helpful comment. We agree that the original wording could be misinterpreted as referring to the ridge heights rather than their elevations. To clarify this, we have revised the sentence to explicitly indicate that the ridges occur at elevations between 14 and 29 meters above sea level (Lines 115–117).

Revision:

… These ridges occur along a steep slope of about 10°, are spaced a few meters apart, and are situated at elevations between 14 and 29 meters above sea level (see Supplementary Table 1).…

L127: Photo "a" is not an aerial image (the shadow of the person taking it is visible). Could you add a scale to photos b-e?

Author's reply:

We thank the reviewer for the comment and apologize for the oversight. Photo (A) is a standard field photograph of the raised beaches along Southernmost Bay, not an aerial image, and this has been clarified in the figure caption. For photos (B–E), we have added 50 cm scale bars, which were established during field photography using reference objects, to accurately indicate the size of the beach boulders.

Revision:

Fig. 3: Field photographs of raised beaches on Inexpressible Island. (A) Overview of raised beaches along Southernmost Bay, viewed from the southwest, with 15 beach ridges labeled A–O. (B–E) Example photographs of well-preserved beach ridges, dominated by large, well-rounded boulders.

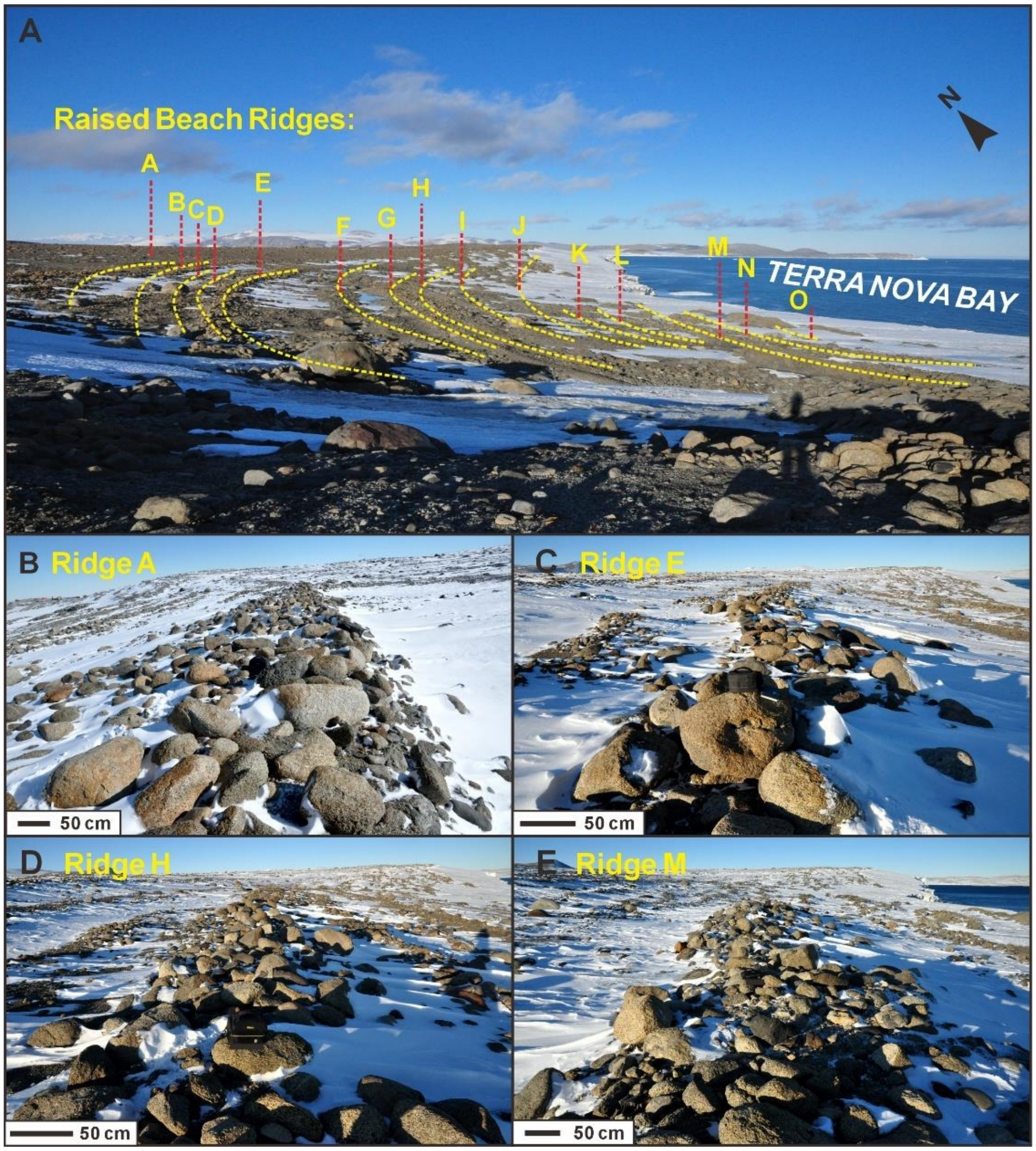

L128: How were the boulders measured?

Author's reply:

We thank the reviewer for this comment and apologize for the lack of clarity in the original text. In the revised version, we have explicitly stated that the boulders were measured manually using a measuring tape during fieldwork (Lines 127–128).

Revision:

…

A total of 688 boulders from 15 raised beach ridges on Inexpressible Island were measured manually using a measuring tape during fieldwork…

L129+L131: Use the same number of decimals.

Author's reply:

We thank the reviewer for pointing this out. In the revised manuscript, we have standardized the number of decimals for boulder measurements to improve consistency and readability (Lines 129–132).

Revision:

… On well-developed beach ridges with prominent relief, the average long-axis boulder size ranged from 48.2 to 72.3 cm, with the largest individual boulder reaching 150.0 cm. Even on less developed ridges, the average long axis exceeded 20.0 cm (Supplementary Table 2).…

L132: Do you have climate/wave data to explain what "conventional" hydrodynamic forcing is for that location?

Author's reply:

We thank the reviewer for this helpful comment. We agree that the previous text did not clearly define what "conventional" hydrodynamic forcing is for Inexpressible Island. In the revised version, we have clarified this by including observational wave data from the 2017 PIPERS campaign. The new text (Lines 133–137) now specifies that the mean significant wave height (SWH) under calm conditions is typically around 0.5 m, providing limited transport capacity, whereas energetic wave events with mean SWHs exceeding 3.0 m can occur when sea-ice concentration is low. This addition provides quantitative context and supports our interpretation that storm waves, rather than fair-weather waves, are the primary drivers of boulder transport and beach ridge formation.

Revision:

… According to wave buoy data collected during the 2017 PIPERS campaign, the mean significant wave height (SWH) in the Ross Sea under calm weather conditions is typically around 0.5 m[43], which offers limited transport capacity. In contrast, when sea-ice concentration is low, energetic wave events with mean SWHs exceeding 3.0 m have been recorded, potentially providing the necessary force to mobilize such large clasts[43,44].

…

L134: Not sure I understand. Is each ridge finer on the landward site or are the innermost ridges finer (and gradually become coarser towards the sea)?

Author's reply:

We thank the reviewer for this insightful comment. The reviewer is correct that our original wording was ambiguous regarding whether the fining trend occurred within individual ridges or among different ridges. Our intended meaning was that each ridge becomes finer toward its landward side. However, as this detail is not critical to the main findings and could cause confusion, we have removed this sentence in the revised version for clarity and conciseness.

L139: I disagree. If the source, lithology and transport pathway of the boulders is unclear, it is difficult to draw conclusions based on the shape alone. Transport and rounding could have happened long before marine processes started acting.

Author's reply:

We thank the reviewer for this valuable comment. We agree that, without clear constraints on the source, lithology, and transport history of the boulders, it would be inappropriate to infer prolonged marine reworking solely from clast roundness. Accordingly, we have removed this sentence in the revised manuscript to avoid overinterpretation.

L143~: I agree that the size of the stones require substantial energy and that the shape of the ridge crests indicate the involvement of wave energy in the depositional processes. Since you had not mentioned it, I was just wondering: How do the modern beaches look (incl. the upper shoreface)? Would you expect any postdepositional changes? Cold, dry, katabatic winds could induce aeolian transport and remove fines.

Author's reply:

We sincerely thank the reviewer for this insightful comment. As shown in Fig. R1, the modern beach environment in Inexpressible Island's South Bay (photographed on 10 February 2023) consists mainly of rounded pebbles and cobbles (typically 10–20 cm along the long axis). Although this site is adjacent to, rather than directly within, the Southernmost Bay where our raised beach samples were collected, both areas display comparable coastal geomorphic settings and sedimentary characteristics. The deposits are actively shaped by wave action during ice-free periods and exhibit similar morphological features to the raised beaches, though with smaller clast sizes.

Fig. R1. Modern beach in South Bay, Inexpressible Island (photographed on 10 February 2023), showing rounded cobbles exposed to wave action.

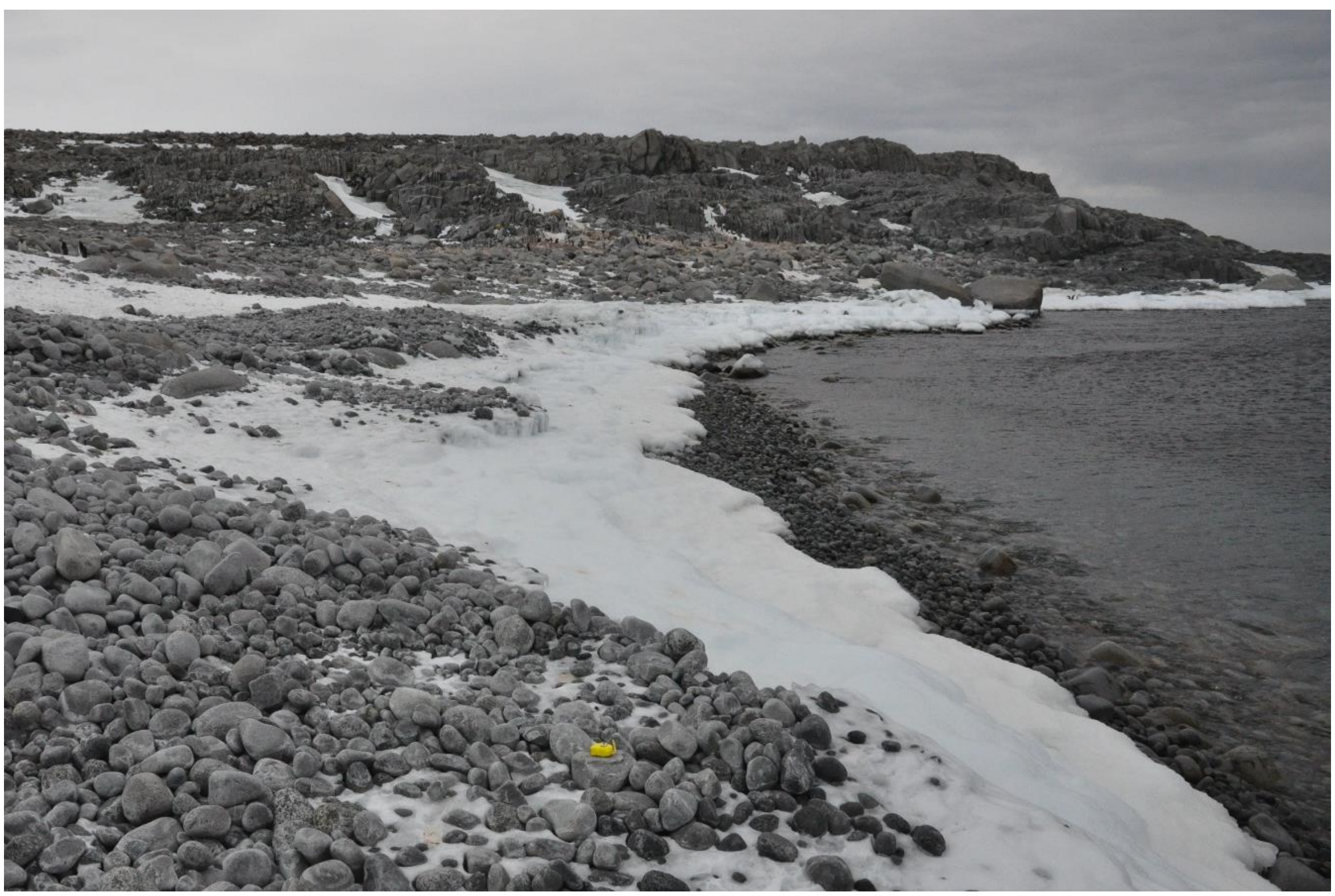

Regarding potential post-depositional modification, we fully agree that cold, dry katabatic winds can exert significant aeolian deflation and abrasion on exposed coastal sediments in Antarctica, preferentially removing finer materials and

altering surface textures. However, our study focuses on large clasts (⩾50 cm), for which aeolian transport is considered negligible under realistic polar wind conditions. While long-term surface weathering or minor edge rounding may occur, we consider it unlikely that wind erosion has substantially modified the long-axis dimensions or overall morphology of these large boulders. Therefore, while acknowledging the potential influence of aeolian processes on fine materials and surface features, our hydrodynamic interpretation is primarily based on the larger, mechanically stable boulders that best preserve their depositional characteristics. We have clarified this context in the revised text to better emphasize that our interpretation of wave-driven deposition relies on the hydrodynamically emplaced boulders rather than finer sediments that could have been modified by wind (Lines 146–149).

Revision:

… While finer materials may have been modified or removed by cold, dry katabatic winds, our analysis focuses on the larger, well-rounded boulders interpreted to have been emplaced by high-energy wave events. When estimating wave energy, we used the second-largest boulder to avoid bias from non-wave-derived clasts.…

L149: Are the ages in the same order as listed in the text (A, E, H, O). If yes, there is an age inversion and the youngest beach is ~5 kyr old.

Author's reply:

We thank the reviewer for pointing this out. The ages in the text are indeed listed in stratigraphic order from ridges A, E, H, and O. We acknowledge that the apparent age inversion between ridges H (3.61 ± 0.75 ka) and O (5.22 ± 0.90 ka). However, these two ages overlap within their 1σ external uncertainties, implying that they may still be statistically consistent within the analytical error. We have revised the text accordingly to clarify this issue and to emphasize that, despite this minor inconsistency, the remaining exposure ages show good internal agreement and correspond well with regional early– to mid-Holocene deglacial records (Lines 156–160).

Revision:

…While three of the samples yield exposure ages clustering in the early to middle Holocene, the youngest age (3.61 ± 0.75 ka) is notably lower than the others. However, this age overlaps within 1σ uncertainty with the 5.22 ± 0.90 ka result from a lower-elevation ridge, suggesting that both may be statistically consistent. Overall, the exposure ages display reasonable internal consistency and align with independent chronological evidence from nearby sites.

…

L150: These 50 radiocarbon ages are crucial for your argument and need to be explained more in the main text – especially regarding their representativeness.

Author's reply:

We thank the reviewer for highlighting the importance of clarifying the ^{14}C dataset and its representativeness. To ensure a more reliable chronological framework for constraining the formation ages of the raised beaches, we invited Prof. Carlo Baroni and Dr. Maria Cristina Salvatore (University of Pisa) to join as co-authors and assist in systematically compiling and

verifying the existing radiocarbon data from Inexpressible Island. The revised dataset now includes 48 published ^{14}C ages, all originally measured by Prof. Baroni, Dr. Salvatore, or their collaborators during Antarctic field campaigns. We have added Supplementary Fig. 4 and Supplementary Table 6 to provide full details for each sample, including sampling location, profile name, coordinates, elevation, dated material, and associated uncertainties.

In the main text, we have also expanded the description of the dataset's representativeness, noting that the ^{14}C ages cover multiple raised beach ridges across the southern coastline and include a range of well-documented organic and biogenic materials (Lines 171–176). These additions clarify the provenance and reliability of the dataset and provide a robust basis for reconstructing the timing of raised beach formation on Inexpressible Island.

Revision:

…These ages derived from a diverse suite of well-documented coastal sites and materials, including organic remains within storm beach deposits[7], seal skin overlying marine sediments[40], penguin remains preserved in ornithogenic soils[7,41,46,47], bulk sediments containing penguin guano[41,48], and in situ marine shells[7,8] (Fig. 4). The dataset spans multiple beach ridges and depositional contexts along the southern coastline, providing a spatially extensive and stratigraphically representative chronology for reconstructing the timing of coastal uplift and beach formation on Inexpressible Island.

…

L155: I can not see how supraglacial transport should matter in the marine deposited ridges, but aeolian reworking, frost transport, or weathering might.

Author's reply:

We sincerely thank the reviewer for this insightful comment. We agree that supraglacial transport is not relevant to boulders deposited within marine raised beaches. In the revised manuscript, we have removed this interpretation and clarified the comparison between the ^{10}Be exposure ages and the radiocarbon ages. The discussion now emphasizes that while some divergence is observed for samples above 15 m elevation, the apparent offset (e.g., 9.1 ± 1.2 ka from ^{10}Be versus 7.1 ± 0.4 ka from ^{14}C) largely falls within the combined external uncertainties of both dating methods. Therefore, the two chronometers are considered to be statistically consistent and likely record the same coastal uplift event rather than a true chronological discrepancy (Lines 187–192).

Revision:

…For example, the ^{10}Be age obtained at 29 m elevation (~9100 ± 1200 yr, external error), which appears approximately 2000 years older than the radiocarbon age of organic material from nearby beach deposits at 30 m (~7055 ± 350 yr, 2σ error). However, when the external uncertainties of both dating methods are taken into account, their age ranges show substantial overlap. This indicates that the apparent offset is within the expected bounds of analytical uncertainty, and both dates likely reflect the same event, without any fundamental contradiction.

…

L166 (Fig. 4): I am a little surprised by the location of the 10Be age for ridge "O" – which appears (in Fig. 3) to be right on the modern shore. Where do all the other samples come from? Also, the text reads as if the ages are form your own previous work (which may be my own interpretation), but they are not. Please mark this very clearly incl. the fact that the date of publication was 20 and >30 years ago.

It could be useful to also plot the ages against distance from shore. How was the age of the beach ridges estimated? I can see no relationship with your 10Be ages?

Author's reply:

We sincerely thank the reviewer for this valuable comment. The sample from ridge "O" was collected from the lowest raised ridge on Inexpressible Island, located at approximately 14 m a.s.l. and positioned several tens of meters inland from the modern shoreline (Fig. R2, Fig. R3). The other samples (ridges A, E, H) were obtained from progressively higher-elevation ridges further inland (Fig. R3).

In response to the reviewer's suggestion, we have revised Fig. 4 and added explicit references in both the figure caption and the main text to clearly acknowledge the original sources of the chronological data. The ^{14}C ages were published in previous studies, including Baroni and Hall (2004), Hall et al. (2023), Gao et al. (2022), Lorenzini et al. (2010, 2012), and Xu et al. (2021). Furthermore, to ensure the accuracy and proper contextualization of these datasets, we invited Prof. Carlo Baroni and Dr. Maria Cristina Salvatore, who originally collected and published part of these data, to join as co-authors.

We are grateful for this valuable comment, which helped us clarify both the sampling context and the provenance of the age dataset in the revised manuscript.

Fig. R2. The beach ridge "O" in Southernmost Bay, Inexpressible Island (photographed on 24 January 2023).

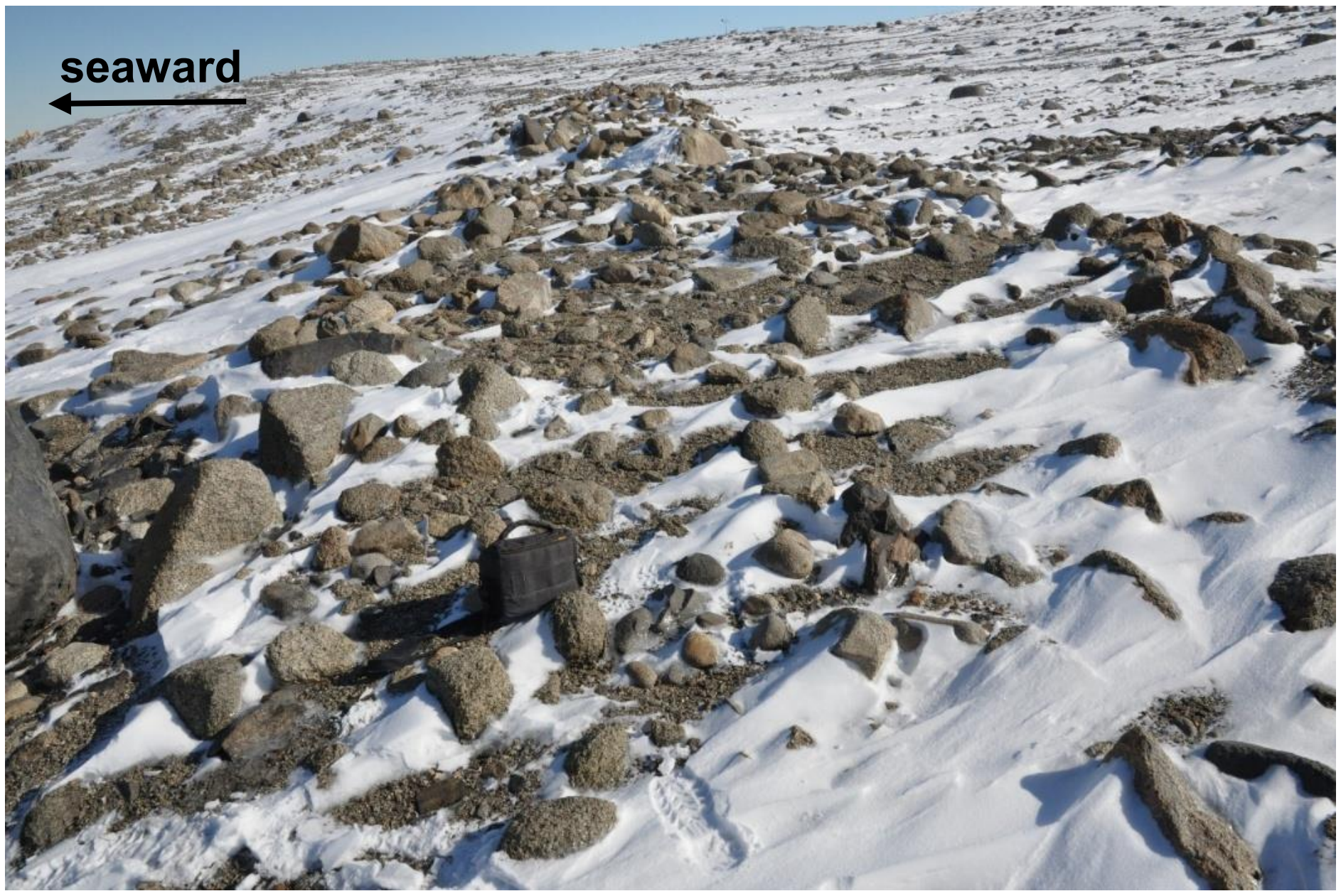

Fig. R3. Google Earth image showing the locations of the ^{10}Be samples collected from the raised beach ridges in Southernmost Bay, Inexpressible Island.

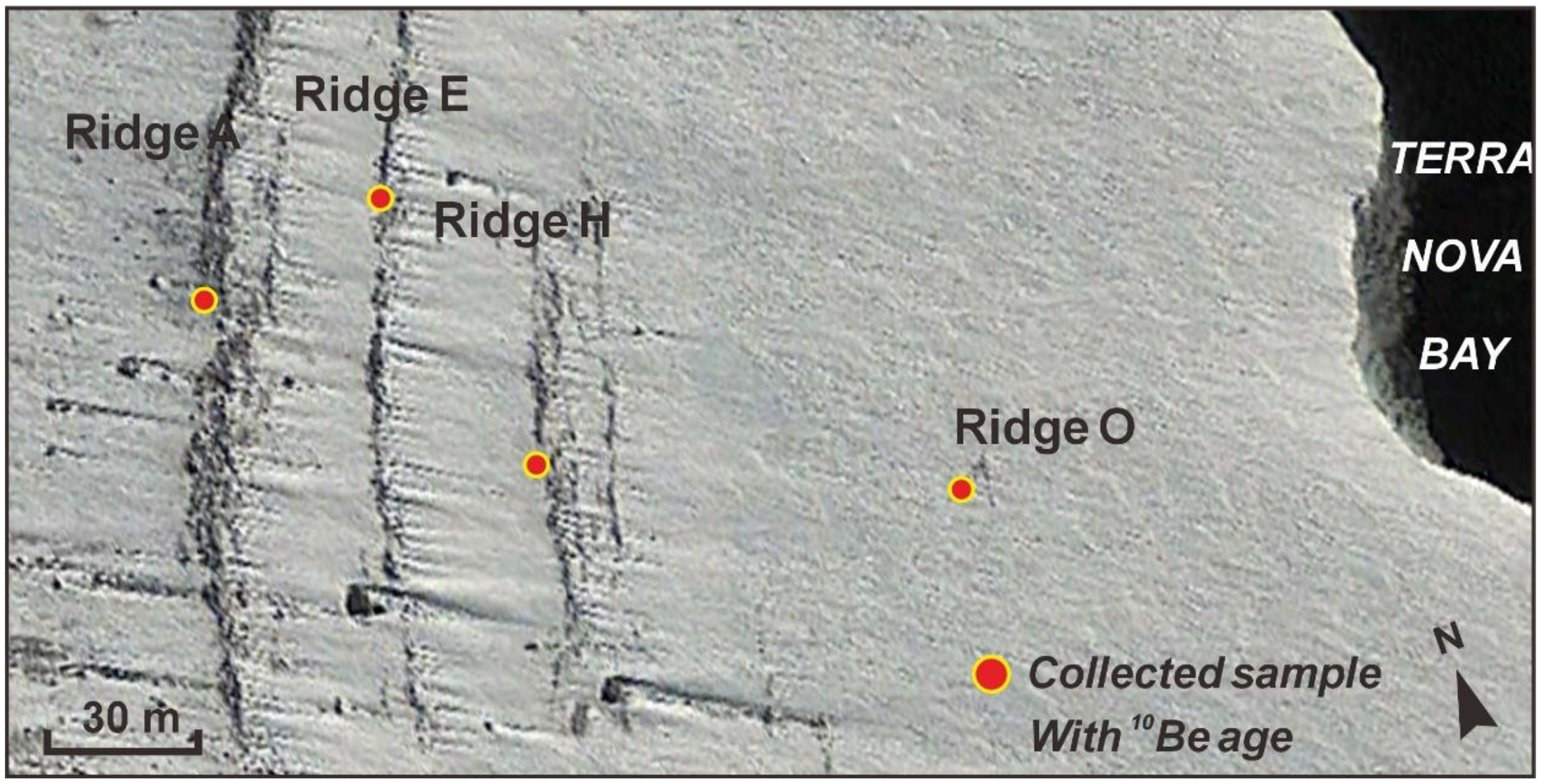

Revision:

Fig. 4: Age–elevation relationship of storm-built raised beaches in Southernmost Bay, Inexpressible Island. ^{10}Be exposure ages, newly obtained in this study, are listed in Supplementary Table 5, and AMS ^{14}C radiocarbon ages from previously published studies (ref f[7,8,40,41,46-48]) are listed in Supplementary Table 6.

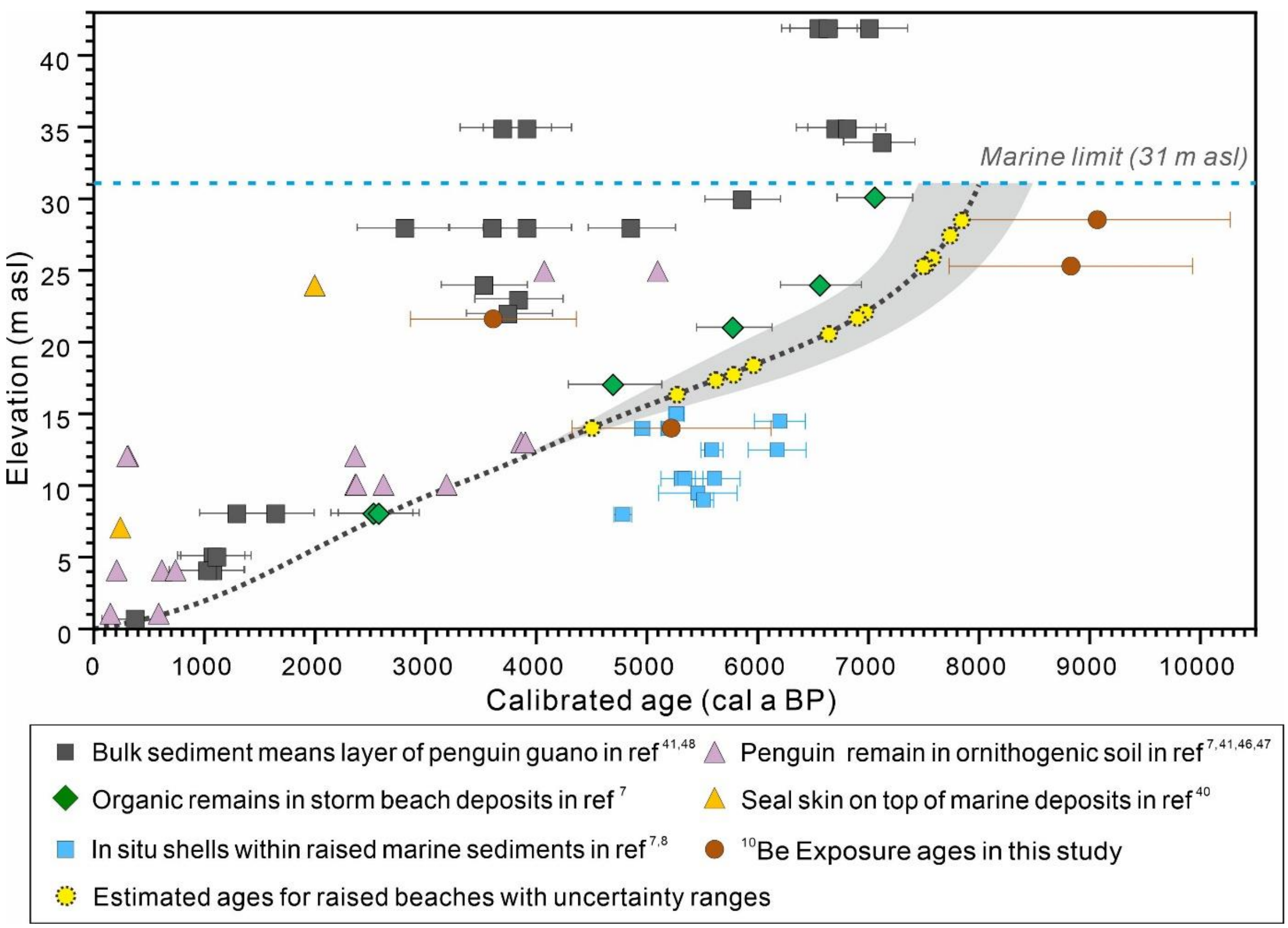

It could be useful to also plot the ages against distance from shore. How was the age of the beach ridges estimated? I can see no relationship with your 10Be ages?

Author's reply:

We thank the reviewer for this suggestion. Ridge ages were not estimated based on distance from the modern shoreline, as accurate measurements of the horizontal distance between the lowest boulder ridge and the shoreline were not recorded during fieldwork. Instead, we used ridge elevation as a proxy for relative formation timing, integrating both ^{10}Be exposure ages and published AMS ^{14}C ages within an age-elevation framework. For ridges with ^{10}Be or ^{14}C ages at corresponding elevations, these values were directly used to constrain formation. For ridges lacking direct dating at the same elevation, linear interpolation between dated elevations was applied to estimate their ages.

We have also clarified the comparison between the ^{10}Be exposure ages and radiocarbon ages. While some divergence is observed for samples above 15 m elevation—for example, 9.1 ± 1.2 ka from ^{10}Be versus 7.1 ± 0.4 ka from ^{14}C—the apparent offset largely falls within the combined external uncertainties of both dating methods. Therefore, the two chronometers are considered statistically consistent and likely record the same coastal uplift event rather than representing a true chronological discrepancy.

This integrated approach provides a consistent chronology of ridge development along the southern coastline of Inexpressible Island, including higher-elevation ridges where ^{14}C-datable materials within beach deposits are sparse. The revised Fig. 4 and main text now clearly explain this method and the associated uncertainties, offering a transparent basis for the inferred ages of the raised beaches (Lines 187–200).

Revision:

…For example, the ^{10}Be age obtained at 29 m elevation (~9100 ± 1200 yr, external error), which appears approximately 2000 years older than the radiocarbon age of organic material from nearby beach deposits at 30 m (~7055 ± 350 yr, 2σ error). However, when the external uncertainties of both dating methods are taken into account, their age ranges show substantial overlap. This indicates that the apparent offset is within the expected bounds of analytical uncertainty, and both dates likely reflect the same event, without any fundamental contradiction.

…

…For ridges with direct ^{10}Be or ^{14}C ages at the corresponding elevations, these were directly applied; for ridges lacking direct dating, linear interpolation between dated elevations was used. An uncertainty margin of ~500–600 years was adopted to reflect the chronological resolution achievable in this region. Based on this approach, ridge A (29 m) likely formed between 8400 and 7300 yrs BP, ridge E (25 m) between 8000 and 7000 yrs BP, ridge H (22 m) between 7400 and 6400 yrs BP, and ridge M (18 m) between 6100 and 5100 yrs BP. These results suggest that raised beach formation on Inexpressible Island occurred episodically during the early to middle Holocene, with prominent ridges forming at roughly one-thousand-year intervals.

…

L204: This is a surprisingly precise estimate of glacier retreat? Any chance there is a wrong unit here?

Author's reply:

We thank the reviewer for this constructive comment. The glacier retreat rate reported by Rhee et al. (2020) (approximately 254 m over 3000 years) indeed appears to overestimate the persistence of local ice during the early Holocene. Subsequent studies have provided robust radiocarbon evidence indicating that the area was already completely deglaciated by around 8.6 ka BP (Gao et al., 2022; Baroni and Orombelli, 1991, 1994; Baroni and Hall, 2004). After careful discussion among the co-authors, we have removed this statement from the revised manuscript to ensure consistency with the well-established deglaciation chronology of the region. This revision provides a more accurate representation of the local glacial history and avoids citing an interpretation that is not fully supported by the broader body of evidence.

L211: For this you need to establish the precise elevation of the ridges and the relationship of that elevation with relative sea level (indicative meaning) and then establish the age of the deposits.

Author's reply:

We thank the reviewer for this insightful comment. We fully agree that constructing a relative sea-level (RSL) curve based on raised beach deposits requires precise determination of ridge elevations, accurate chronological control, and a well-defined relationship between ridge altitude and contemporaneous sea level, as demonstrated in previous studies (e.g., Baroni et al., 1991; Hall and Denton, 1999; Baroni et al., 2004; Bentley et al., 2005; Hall et al., 2010; Braddock et al., 2022). In our study, ridge elevations were obtained through field measurements, formation ages of storm beaches were constrained using combined ^{10}Be exposure and AMS ^{14}C dating, and wave run-up heights were quantitatively estimated to define the relationship between beach-crest elevation and sea level at the time of formation.

In the revised manuscript, Fig. 5 now clearly presents the formation ages of storm-built ridges and their associated uncertainties, with vertical bars representing the relative relationship between ridge elevation and contemporaneous sea level (i.e., the minimum wave run-up calculated in this study). We have removed the RSL reconstruction from the Results section and instead provide a careful discussion in the first paragraph of the Discussion, emphasizing that the formation of raised beaches in our study area reflects the deglaciation history of Terra Nova Bay, subsequent glacial isostatic adjustment, and relative sea-level changes (Lines 249–265).

Revision:

…The raised beach ridges developed after the retreat of the Ross Ice Shelf grounding line, when the coastline became exposed to open-marine conditions and subjected to wave reworking. Their formation reflects the combined influence of relative sea-level fluctuations, sediment availability, and high-energy storm-wave events. Reconstructing the wave conditions required to build these ridges therefore offers valuable insights into coastal evolution following deglaciation. Well-developed ridges typically show a relative relief of ~2–3 m and contain boulders exceeding 1 m in diameter—hallmarks of high-energy storm events. In our analysis, storm-wave run-up heights were derived from the minimum and maximum wave heights required to initiate sliding and rolling of the largest transportable boulders, with the sliding mode providing a more conservative (lower) estimate of the minimum wave height. For instance, for ridge A at 29 m elevation, model results indicate that sliding and rolling initiation would require waves exceeding 3 m and 6 m, respectively. Taking the water level during ridge formation as the sum of contemporaneous sea level and wave run-up height, and applying the sliding-mode estimate for correction, we infer that post-deglacial coastal uplift at this site was likely around 26 m. By integrating wave-

height reconstructions from multiple ridge levels with chronological data, we find that—despite uncertainties in storm energy and formation age that limit the precision of our relative sea-level curve—the overall pattern aligns with the W12[18] GIA model as well as ICE-5G[54] and ICE-6G[19]. Higher or lower assumed storm-wave energies would proportionally modify the inferred rates of postglacial rebound.

…

L235 (Fig. 5): The conceptual model bears striking resemblance with the model from Lindhorst & Schutter (2014, Geomorphology), including the wavy surface for the erosional unconformity. This needs to be marked clearly. The RSL curve by Baroni & Hall 2004 is based on a scatter of limiting ages and your adjusted curve probably fits well within the window of uncertainty. Where do the new details come from?

Author's reply:

We sincerely thank the reviewer for this insightful comment. We have carefully revised Fig. 5 and removed the conceptual model representation. The updated Fig. 5 now focuses on illustrating how the raised beach sequence on Inexpressible Island reflects post-glacial ice retreat, glacio-isostatic adjustment, and storm-wave processes, rather than serving as a schematic model. We have also expanded the relevant discussion to clarify that our results are broadly consistent with the relative sea-level (RSL) curve proposed by Baroni & Hall (2004). However, our new chronological data from beaches above approximately 18 m elevation tend to indicate a slightly earlier onset of glacier retreat and coastal emergence. We acknowledge that the adjusted curve fits within the uncertainty range of previous reconstructions, and our interpretation aims to complement rather than replace existing RSL models (Lines 230–248).

Revision:

…

Our reconstruction of the raised beach sequence from Southernmost Bay of Inexpressible Island provides new chronological constraints on glacial retreat history of Terra Nova Bay and its subsequent GIA. Overall, our results are consistent with the widely cited sea-level curve[7], based on radiocarbon dating of ~100 organic samples. However, the ages of raised beaches above ~18 m elevation tend to indicate a somewhat earlier timing of glacier retreat and beach emergence (Fig. 5). During the Last Glacial Maximum (LGM), outlet glaciers in the region coalesced into a piedmont glacier, reaching ~400 m above present sea level and merging with grounded ice in the Ross Sea[7,8,49]. Our ^{10}Be exposure ages indicate that the oldest preserved beach ridge, situated ~29 m above present sea level, formed between 8.4-7.3 ka, implying that the coastline of Terra Nova Bay had already become ice-free and exposed to marine processes by that time. This interpretation is supported by independent evidence, including penguin guano deposits at ~34 m elevation on Inexpressible Island (7.1 ± 0.3 ka, 2σ)[41]. We infer that glacier retreat began in the early Holocene, creating open-water conditions that facilitated the formation of the earliest beach ridges (Fig. 5).

This conclusion aligns with recent records from the southern Transantarctic Mountains and the Scott Coast, which indicate rapid grounding-line retreat and thinning between 9–8 ka BP[50]. Our data suggest that Terra Nova Bay may also have experienced open-water conditions by ~8 ka BP, implying earlier glacier retreat than previously inferred. These findings support the hypothesis of an early and rapid Holocene deglaciation in this sector of Antarctica, during which most of the

grounded ice in the western and central Ross Sea was removed in less than two millennia. Notably, this deglaciation phase appears to be independent of the MWP-1A meltwater pulse event[45,51-53]….

Fig. 5: Postglacial coastal evolution and relative sea-level changes inferred from storm-built ridges at Inexpressible Island. (A) Inferred RSL trend reconstructed from the formation ages and elevations of storm-built beaches, with vertical bars indicating elevation adjustments for minimum wave run-up heights calculated using the siding transport mode. The curve is compared with a recalculated RSL reconstruction from ref[7] (updated with Marine20 calibration dataset and reservoir corrections[10]) and with GIA model predictions. (B) Deglaciation stage with no preserved ridges. (C) Formation of earliest ridges during marine highstand under storm-wave conditions. (D) Progressive postglacial uplift and development of successive storm-built ridges during episodic high-energy events.

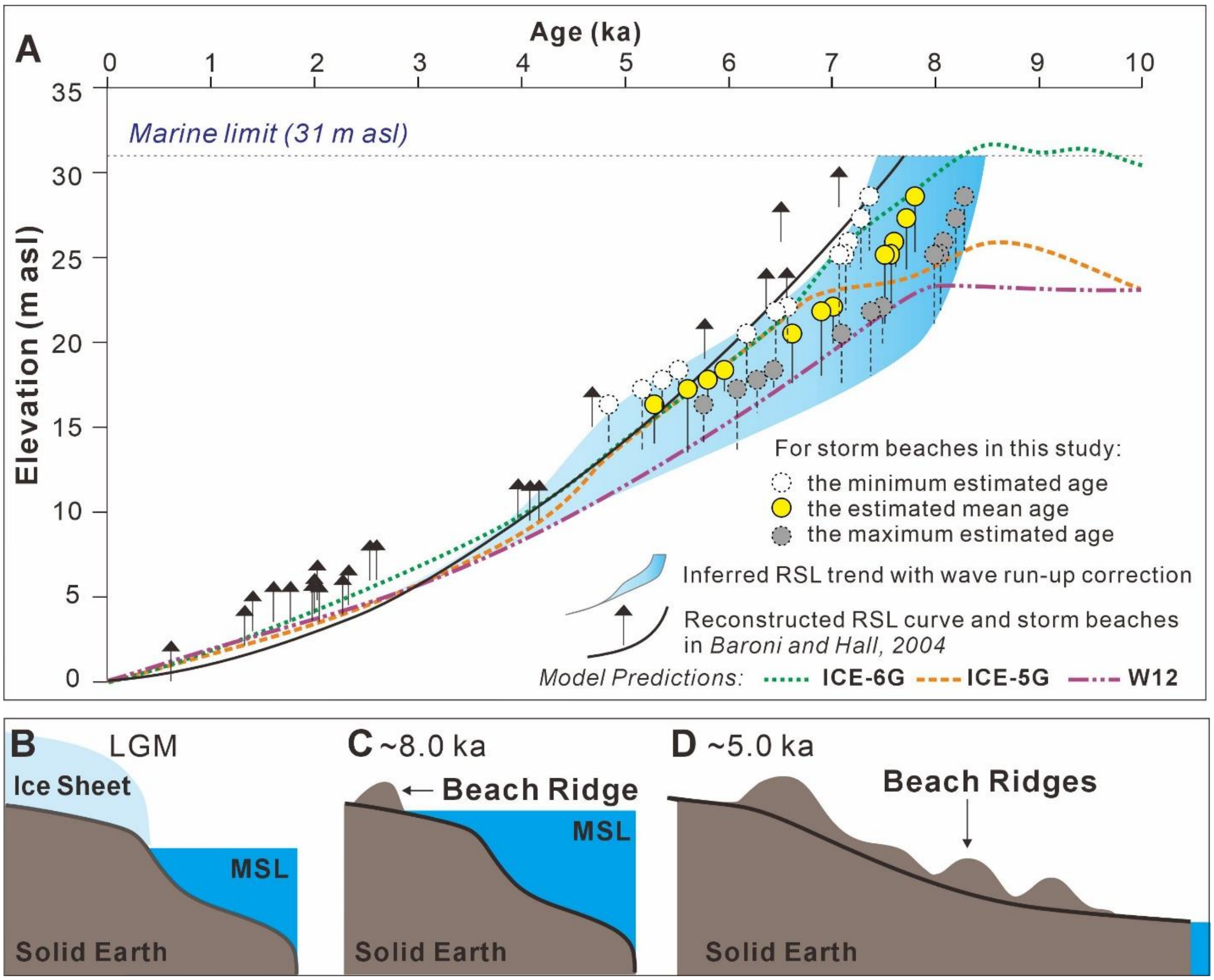

Response to Reviewer #2:

It was a treat to read this manuscript, and most of the arguments are intriguing and the conclusions informative. However, I find the inferences of the wave amplitudes from which these wave heights are derived oversimplified and therefore spurious. Storm waves are random processes. The linear assumption between velocity and speed that is employed is a gross underestimation of the uncertainty that is generated by constructive and destructive wave-wave interaction that could result in the movement of boulders. I understand that the employed equations are simple, but other models of boulder transport have shown that nonlinearity of the wave and the interaction of the boulder with the moving fluid during the transport process are important considerations. Without the proper treatment of the nonlinearities of the transport process, the calculated values should not be trusted. A more sophisticated model would also allow for a more robust statistical analysis of the inverted wave heights and what they mean. Because of these flaws, I cannot recommend this manuscript for publication.

Author's reply:

We thank the reviewer for the detailed and constructive comments. We fully acknowledge that the nonlinearity of waves and the interaction of boulders with the moving fluid during transport are important considerations, and that neglecting these nonlinearities can introduce uncertainties in calculated wave heights.

In the study of extreme wave events and boulder transport on beaches, two main approaches are commonly used: forward modeling and inverse modeling. Forward modeling uses hydrodynamic numerical models to simulate boulder transport under various wave conditions, and the results are then used to constrain past extreme wave events (e.g., Imamura et al., 2008; Nandasena and Tanaka, 2013). Methods such as the nonlinear shallow water equations and nonlinear long-wave equations have been applied to simulate the mechanisms of boulder movement under storm waves (Scardino et al., 2025). While forward modeling can theoretically provide more accurate simulations, its practical application faces challenges: (1) numerical models involve highly nonlinear calculations that require simplifying assumptions (e.g., uniform wave propagation, fixed coastal topography), which introduces uncertainties in the absence of experimental validation; (2) model outputs strongly depend on accurate reconstructions of paleo-coastlines, bathymetry, and water depths, which are often unavailable or uncertain in Quaternary geomorphological studies (Wang et al., 2020, 2022). For example, Minamidate et al. (2020, 2022) simulated storm-wave-induced boulder transport on Kudaka Island, Japan, over the past 3500 years, but assumptions of mean sea level and neglect of local coastal evolution could bias the results, limiting their applicability to real extreme events. In Antarctica, systematic field validation and numerical simulations of extreme wave events are scarce, making the direct use of highly nonlinear forward models challenging.

Inverse modeling, on the other hand, estimates the minimum flow velocity or wave height required to mobilize boulders of known size, density, and shape, thereby inferring extreme wave event intensity. First introduced by Nott (1997, 2003), this approach has been widely applied in geomorphology and Quaternary studies due to its intuitive logic and practical applicability (Engel and May, 2012; Pepe et al., 2018). While it also has limitations—such as uncertainties in drag and lift coefficients (Sugawara et al., 2014; Nandasena et al., 2022; Kennedy et al., 2021), and assumptions that nearshore wave height equals offshore wave height—this approach remains the most feasible for reconstructing Holocene storm-wave events in remote regions like Inexpressible Island.

To address the reviewer's concerns, we have carefully revised our calculations and incorporated additional hydrodynamic considerations. We employed the Nandasena et al. (2011) model, which builds upon Nott (2003) and explicitly accounts for beach slope in boulder transport. In this model, boulders are treated as rectangular blocks, and the combined effects of drag, lift, gravity, buoyancy, and friction are used to derive the critical flow velocities required for initiation of motion, including sliding, rolling, and saltation modes. Field-measured boulder volumes, densities, and dimensions (axes L, I, S), together with laboratory-determined rock densities, were used to calculate these critical velocities, ensuring that estimates are specific to the boulders at each ridge.

We further refined our treatment of nearshore wave conditions by explicitly considering wave transformation in shallow water and wave breaking. Breaking-point wave heights (H_b) were calculated using the Sunamura–Horikawa equation, and corresponding water depths (h_b) were estimated following Collins' empirical formula. Effective wave heights at the shore were then inferred using the shallow-water wave equation ($u = \delta\sqrt{gH}$), with δ parameterized to reflect wave-breaking conditions during storm events. This approach allows a more realistic estimation of the hydrodynamic forces acting on boulders while accounting for the differences between deep-water, breaking-point, and nearshore wave heights (Lines 417–452).

To constrain the hydrodynamic environment, we integrated modern observations and numerical simulations from the Ross Sea and the Terra Nova Bay Polynya. Wave buoy data from the 2017 PIPERS project, together with high-resolution numerical simulations of strong wind events (Hermann, 2024), provide realistic estimates of significant wave heights and periods under nearshore conditions. These data were used to validate the calculated flow velocities and compare them with the reconstructed elevations of raised beaches, demonstrating that storm waves of the estimated magnitude were sufficient to mobilize boulders and form the Holocene storm-built ridges (Lines 271–298). By combining field measurements, laboratory data, and observational constraints, this method provides a conservative yet robust estimation of the storm-wave conditions responsible for boulder transport on Inexpressible Island during Holocene.

Future studies incorporating more precise reconstructions of paleo-coastlines and sea level, together with modern wind and wave buoy observations, could apply refined hydrodynamic models to simulate and validate wave conditions, potentially improving reconstructions of Holocene storm events.

Revision:

…

Based on boulder size and beach ridge morphology, we infer that between approximately 8 and 5 ka, the coastal environment of Inexpressible Island experienced a prolonged period of enhanced storminess, during which multiple exceptionally energetic storm episodes generated significant wave heights exceeding 3 m and occasionally approaching 6 m, accompanied by several moderate events around 2 m. Rather than representing isolated extreme incidents, these high-energy events likely occurred repeatedly over millennial timescales, with cumulative wave impacts driving boulder entrainment, clast redistribution, and progressive ridge progradation. The estimated wave heights thus represent the critical nearshore conditions necessary to initiate boulder motion under sustained extreme storm regimes. To evaluate the plausibility of these reconstructed values, we compared them with modern observational and modeled data from the Ross Sea and the Terra Nova

Bay Polynya (TNBP). The PIPERS project (Polynyas, Ice Production, and seasonal Evolution in the Ross Sea), conducted in the seasonal sea-ice zone, recorded significant wave heights exceeding 2 m during strong katabatic wind events in the Terra Nova Bay Polynya (April 2017) using SWIFT drifters[35]. Numerical simulations of ten intense wind events occurring between 2016 and 2021 (in September–October) in the same region produced typical significant wave heights of around 3 m, with maximum values exceeding 5 m under ice-free conditions[36]. In the broader Ross Sea region, wave buoy data from the 2017 PIPERS campaign documented several extreme events, with mean significant wave heights reaching 6.3 m, deep-water wave heights exceeding 7 m, and wave periods of 6–10 s[43,44].

These modern observations and simulations provide an important reference for evaluating our reconstructed Holocene wave heights. For ridges composed of smaller clasts, the calculated significant wave heights are around 2 m, whereas the largest ridges, containing meter-scale boulders, require waves exceeding 3 m, and up to ~6 m under rolling transport conditions, to initiate motion. Although these values are higher than those observed during most modern extreme events in the TNBP, they remain within the upper range of simulated ice-free scenarios, indicating that our estimates are hydrodynamically realistic. Importantly, these reconstructions represent millennial-scale storm regimes that were far rarer and more energetic than short-term modern events, thus providing a longer-term perspective on extreme wave activity in the Ross Sea. The close correspondence between reconstructed and modeled magnitudes further suggests that wave energy during the early Holocene was more vigorous than at present, reflecting a period of reduced sea-ice cover, expanded open-water fetch, and intensified atmosphere–ocean coupling.…

…

In the Ross Sea region, wave buoy data collected during the 2017 PIPERS project revealed several significant wave events. In late June, a major wave event recorded an average SWH of approximately 6.3 m, with deep-water wave heights exceeding 7 m and marginal ice zone wave heights surpassing 5 m. During events where SWH exceeded 4 m, wave periods ranged from 6 to 10 second[43,44] These observations are consistent with the predictions from the WAVEWATCH III (WW3) model simulations presented by ref[79]. Based on these data, we assume a representative deep-water significant wave height (H_0) of 7 m and a wave period (T_0) of 6 s during major storm events affecting the western coast of Inexpressible Island. Using the deep-water wave length equation $L_0 = \frac{gT^2}{2\pi}$, the resulting wave length is approximately 56 m. Field measurements indicate an average beach slope (tan θ) of 10° in the southern bay area.

Applying these parameters to the wave run-up formula[80]:

$$R_{2\%} = 1.1\left[0.35 \tan\theta \sqrt{(H_0 L_0)} + 0.5\sqrt{(H_0 L_0)(0.563 \tan^2\theta + 0.004)}\right],$$

we estimate a maximum run-up height ($R_2\%$) of approximately 3.19 m. This value exceeds the relief of the raised beaches in the area, which ranges between 0.5 and 3.09 m, indicating that storm waves of this magnitude were indeed capable of overtopping and reworking the beach surface, leading to the formation of storm surge-related sedimentary deposits.

To further determine whether wave breaking occurred in the nearshore zone, we analyzed the relationship between the wave height and water depth at the breaking point. The breaking wave height H_b was estimated using the empirical equation proposed by Sunamura and Horikawa[81]:

$$\frac{H_b}{H_0} = (\tan\beta)^{0.2}\left(\frac{H_0}{L_0}\right)^{-0.25}$$

where β represents the average slope of the nearshore profile from the shoreline to a depth of 20 m.

The corresponding water depth at the breaking point h_b was estimated using the empirical relationship provided by Collins[82]:

$$\frac{H_b}{h_b} = 0.72 + 5.6\tan\beta$$

The calculated wave height at the breaking point (8.32 m) and corresponding water depth (4.88 m). This indicates that waves in the nearshore zone of Inexpressible Island were already in a breaking regime, enhancing their capacity to mobilize large boulders through rolling or saltation processes.

Accordingly, the critical wave height required to initiate boulder transport can be estimated based on the critical flow velocity using the equation $u = \delta\sqrt{gH}$, where H is the wave height (in meters), and δ is a wave type parameter. Specifically, when a storm occurs and wave breaking is present, $\delta = 0.5$; when a storm occurs without wave breaking, H is defined as the breaking wave height H_b, and $\delta = 1$; for tsunami conditions, $\delta = 2$[23]. In this study, δ is set to 0.5, reflecting the assumption of wave breaking during storm events. Based on the boulder size, density, and elevation, we then calculated the wave period conditions necessary to initiate boulder motion using the following equation[83]:

$$T = \frac{2}{g}\left(\frac{\rho_w}{\rho_s - \rho_w}\right)\left(\frac{C_d}{tan\,\theta}\right)\left(\frac{h_{clast}}{D_i}\right)u$$

where h_{clast} is the vertical displacement of the boulder (m), defined as the difference between the ridge relief and the boulder's height (short axis), and D_i is the intermediate diameter of the transported clast (m).

…

Response to Reviewer #3:

Dear authors,

The manuscript by Wang et al., focus on the use of boulder beach ridges to reconstruct sea-level and extreme wave patterns from Mid-Holocene along the coast of Inexpressible Island (Antarctica).

The manuscript is well-written and provides informative figures that support the reasoning forwarded. The objectives of the manuscript are clear and are based on the use of raised boulder beaches to reconstruct storminess patterns along the Ross Sea. The manuscript is structured on a compination of field data (topographic measurements and particle size and shape measurements), age-estimation methodologies (cosmogenic and raiocarbon) and hydrodynamic modelling. This approach is sound and provides good basis for the discussion forwarded by the authors.

Overall, I have to commend the authors on their work. Nevertheless, the approach followed is classical and based on a single study area, even if it is in Antarctica. The innovative character of the work lies on the location studied. Having said this, conceptually and methodologically the approach made by the authors is robust and the interpretation seem well-supported by the data presented.

My main concerns are with the lack of geographical scope, with the height estimations, with the reliability of some of the dates obtained, the particle shape inferences, the particle transport equations used and with the storminess vs extreme events interpretation. I will detail some of my concerns below. Nevertheless, I would like to stress that my intention is to seek further clarifications and not to disregard or question the work and interpretations of the authors. I do believe they did an excellent work.

So, in detail:

1- Geographical scope - the fact that there is limited (in geographical area) evidences presented is a cause for some concern and ideally correlation with other locations need to be further stressed on the manuscript;

Author's reply:

We thank the reviewer for this valuable comment. We agree that the geographic scope of our field evidence is limited to Inexpressible Island. In the revised manuscript, we have strengthened both the Introduction and Discussion to better highlight the regional representativeness and broader implications of our findings.

In the Introduction, we now provide a more comprehensive synthesis of raised boulder beaches identified along the Antarctic coast (Fig. 1), emphasizing that, although such geomorphic archives are spatially restricted, they provide rare and valuable archives of extreme wave activity and coastal environmental change (Lines 48–58). The revised text clarifies that raised boulder beaches are mainly concentrated in the Ross Sea and Antarctic Peninsula, and that our study site, located along the western Ross Sea coast, fills a critical spatial gap in Holocene wave reconstructions (Lines 80–84). Given the persistent open-water conditions maintained by the Terra Nova Bay Polynya, the Inexpressible Island record provides an ideal natural laboratory to investigate storm-wave processes and their relationship with deglaciation and coastal evolution in this region (Lines 88–98).

In addition, the Discussion has been expanded to include comparisons with deglaciation and paleoenvironmental records from the southern Transantarctic Mountains and the Scott Coast, which together illustrate that the raised beaches of Inexpressible Island reflect regional patterns of ice retreat, glacio-isostatic adjustment, and sea-ice variability (Lines 242–248). We also integrated multiple proxy datasets, including ice-core and marine sediment records, to link enhanced storm-wave activity with reduced sea-ice extent and intensified ocean–atmosphere interactions during the early to mid-Holocene.

Overall, while the field evidence is geographically limited, we believe that the geomorphic, chronological, and paleoenvironmental records from Inexpressible Island provide regionally representative insights into Holocene coastal dynamics in the Ross Sea sector, complementing existing studies from the Antarctic Peninsula and improving spatial coverage for understanding Antarctic extreme wave activity.

Revision:

…Raised boulder beaches have been identified along several Antarctic coastlines (Fig. 1), predominantly in the Ross Sea and the Antarctic Peninsula, with fewer occurrences in East and West Antarctica[6-13]. Existing evidence indicates that periods of intensified beach formation often coincided with reduced sea-ice extent and enhanced wave activity. For instance, granulometric records from Calmette Bay show increased beach development at approximately 1–2 and 5–6 ka, accompanied

by more rounded clasts that reflect intensified wave reworking[6]. Similar observations from Anchorage Island and Rothera Station indicate heightened wave energy between 2.2 and 3.5 ka[9], while records from Horseshoe Island suggest enhanced wave activity and reduced summer sea ice between 2 and 6 ka[14]. Comparable relationships between diminished sea-ice cover and increased beach formation have also been reported from other high-latitude coasts, including the Varanger Peninsula in Arctic Norway, the Kola Peninsula in northwest Russia, and Lowther Island in the Canadian Arctic[15-17].

…

…In Antarctica, most studies, mainly from the Antarctic Peninsula, have relied on qualitative indicators such as sediment roundness to infer relative wave intensity[6,9,14]. Earlier studies assumed that Holocene storm-raised beaches formed 2–3 m above sea level[7,12,22], whereas modern beaches along the Ross Sea coast can reach up to 4 m[30], suggesting that previous estimates may have underestimated the magnitude of Antarctic extreme waves.

…For instance, granulometric records from Calmette Bay show increased beach development at approximately 1–2 and 5–6 ka, accompanied by more rounded clasts that reflect intensified wave reworking[6]. Similar observations from Anchorage Island and Rothera Station indicate heightened wave energy between 2.2 and 3.5 ka[9], while records from Horseshoe Island suggest enhanced wave activity and reduced summer sea ice between 2 and 6 ka[14]. Comparable relationships between diminished sea-ice cover and increased beach formation have also been reported from other high-latitude coasts, including the Varanger Peninsula in Arctic Norway, the Kola Peninsula in northwest Russia, and Lowther Island in the Canadian Arctic[15-17].

… The TNBP always connects to the open sea directly in summer, with its area peaking at about 5000 km², while during the cold season it remains partially open, with a mean area of about 2361 km² (Fig. 2)[33,34]. These conditions enable the generation of local waves reaching 2–3 m in height even when most of the Ross Sea remains ice-covered, and allow the intrusion of Southern Ocean swells during summer when regional sea ice retreats[35,36]. Geological and paleoenvironmental evidence indicates that the TNBP has persisted for several millennia, maintaining open-water conditions that shaped coastal sediment dynamics[37-40]. Marine diatom and sediment records reveal millennial-scale variability in polynya efficiency and coastal sea-ice extent since the mid-Holocene[37]. Geomorphic and biological indicators, such as wave-formed raised beaches[8,38,39], Adélie penguin colonies[41], and elephant seal remains along the Victoria Land coast[40], further suggest the long-term persistence of open-water conditions in Terra Nova Bay. …

…

This conclusion aligns with recent records from the southern Transantarctic Mountains and the Scott Coast, which indicate rapid grounding-line retreat and thinning between 9–8 ka BP[50]. Our data suggest that Terra Nova Bay may also have experienced open-water conditions by ~8 ka BP, implying earlier glacier retreat than previously inferred. These findings support the hypothesis of an early and rapid Holocene deglaciation in this sector of Antarctica, during which most of the grounded ice in the western and central Ross Sea was removed in less than two millennia. Notably, this deglaciation phase appears to be independent of the MWP-1A meltwater pulse event[45,51-53].

…

2- Height estimations - in one part of the manuscript you refer to have used DGPS-RTK measuments but to calculate the slope of the beach berm you refer to have used a hand-set GPS (typically with no RTK and with a very high associated height error). This is critical and should be clarified in the manuscript;

Author's reply:

We sincerely thank the reviewer for this helpful comment and apologize for the lack of clarity in our original description. In fact, all elevation data in this study were obtained using a high-precision GPS-RTK (Real-Time Kinematic) system, which provides centimeter-level accuracy for both horizontal and vertical positioning. The beach slope value was subsequently calculated from the relative elevation differences measured over short horizontal distances using the same RTK dataset. To avoid confusion, we have revised the Methods section to clarify this point (Lines 364–367).

Revision:

…High-precision GPS-RTK (Real-Time Kinematic) surveys were then carried out to measure the elevation, geographic coordinates, and topographic profiles of the beach ridges. The slope value was derived from the relative elevation differences obtained from the same RTK dataset, ensuring centimeter-level accuracy for both horizontal and vertical positioning.…

3- Age-estimation - you solely present 4 new cosmogenic dates and use 50 radiocarbon dates previously published. This might be insufficient especially when the 10Be dates show such discrepancies and deviations. Chronological accuracy is pivotal for this work and Figure 4 exposes the 10Be dates as outlyers. Therefore, further caution should be added on some of the chronological interpretations forwarded later on the manuscript;

Author's reply:

We sincerely thank the reviewer for highlighting this important point. We fully agree that chronological accuracy is crucial for interpreting raised beach formation. In this study, we present four new ^{10}Be exposure ages alongside 48 previously published radiocarbon ages. The limited number of new ^{10}Be ages reflects logistical constraints during our field campaign, which restricted the number of samples that could be collected and transported for analysis.

To strengthen the chronological framework, we invited Prof. Carlo Baroni and Dr. Maria Cristina Salvatore as co-authors, given their extensive prior fieldwork and ^{14}C measurements in the study area. As collaborators, they systematically reviewed and verified the published ^{14}C dataset, most of which was obtained by themselves or their collaborators, and supplemented additional details including sampling location, profile, elevation, dated material, and associated uncertainties (Lines 171–176). This allowed us to discuss the formation ages of the raised beaches with greater care and confidence.

We acknowledge that some ^{10}Be ages appear as outliers when compared with radiocarbon ages. For example, the ^{10}Be age of 9.1 ± 1.2 ka is slightly older than the corresponding ^{14}C age of 7.1 ± 0.4 ka at similar elevations; however, this difference largely falls within the combined external uncertainties of both dating methods (Lines 187–192). Overall, the ^{10}Be ages display reasonable internal consistency: three samples cluster in the early to middle Holocene, while the youngest age (3.61 ± 0.75 ka) is lower than the others but overlaps within 1σ uncertainty with the 5.22 ± 0.90 ka age from a lower-elevation ridge (Lines 156–160). Furthermore, the ^{10}Be ages are supported by independent geological and biological evidence from Inexpressible Island and Terra Nova Bay, including the onset of deglaciation (~11 ka BP), the earliest open-water polynya

conditions (~8.6 ka BP), and the presence of penguin nesting sites and elephant seal occupation on ice-free beaches during the early Holocene (Lines 161–168).

To provide a consistent chronological framework, we used ridge elevation as a proxy for relative formation timing, integrating both ^{10}Be and ^{14}C ages within an age-elevation framework. For ridges lacking direct dating at the same elevation, linear interpolation between dated elevations was applied. This approach, together with careful consideration of analytical uncertainties, allows us to infer the sequence of beach ridge formation robustly, while acknowledging the inherent limitations of the dataset (Lines 193–200).

Revision:

…These ages derived from a diverse suite of well-documented coastal sites and materials, including organic remains within storm beach deposits[7], seal skin overlying marine sediments[40], penguin remains preserved in ornithogenic soils[7,41,46,47], bulk sediments containing penguin guano[41,48], and in situ marine shells[7,8] (Fig. 4). The dataset spans multiple beach ridges and depositional contexts along the southern coastline, providing a spatially extensive and stratigraphically representative chronology for reconstructing the timing of coastal uplift and beach formation on Inexpressible Island.

…

…For example, the ^{10}Be age obtained at 29 m elevation (~9100 ± 1200 yr, external error), which appears approximately 2000 years older than the radiocarbon age of organic material from nearby beach deposits at 30 m (~7055 ± 350 yr, 2σ error). However, when the external uncertainties of both dating methods are taken into account, their age ranges show substantial overlap. This indicates that the apparent offset is within the expected bounds of analytical uncertainty, and both dates likely reflect the same event, without any fundamental contradiction.

…

…While three of the samples yield exposure ages clustering in the early to middle Holocene, the youngest age (3.61 ± 0.75 ka) is notably lower than the others. However, this age overlaps within 1σ uncertainty with the 5.22 ± 0.90 ka result from a lower-elevation ridge, suggesting that both may be statistically consistent. Overall, the exposure ages display reasonable internal consistency and align with independent chronological evidence from nearby sites.

These results correspond well with the onset of deglaciation on Inexpressible Island, beginning around 11 ka BP[45], and with the establishment of open-water polynya conditions in Terra Nova Bay by at least ~8.6 ka BP, as indicated by the earliest radiocarbon-dated Adélie penguin remains[41]. The presence of penguin nesting sites above the marine limit further supports ice-free conditions by at least 7.1 ka BP (Fig. 4; Supplementary Table 6). Additionally, evidence from previous studies suggests that elephant seals gained access to ice-free beaches along the Victoria Land coast during the early Holocene; in particular, abundant desiccated skin fragments preserved on the raised beach at ~30 m elevation indicate sustained occupation of the study area by elephant seals at that time[40].

…

…For ridges with direct ^{10}Be or ^{14}C ages at the corresponding elevations, these were directly applied; for ridges lacking direct dating, linear interpolation between dated elevations was used. An uncertainty margin of ~500–600 years was adopted

to reflect the chronological resolution achievable in this region. Based on this approach, ridge A (29 m) likely formed between 8400 and 7300 yrs BP, ridge E (25 m) between 8000 and 7000 yrs BP, ridge H (22 m) between 7400 and 6400 yrs BP, and ridge M (18 m) between 6100 and 5100 yrs BP. These results suggest that raised beach formation on Inexpressible Island occurred episodically during the early to middle Holocene, with prominent ridges forming at roughly one-thousand-year intervals.

…

4- Particle shape, size, hydrodynamic equations and genetic events - rounded particles, namemly gravel and boulders, tend to be a result of reccurrent action from waves. In fact, boulder ridges are mostly associated with the long-term succession of storm waves and not with single events. One aspect that I would like the authors to improve is to clarify if we are analysing gravel, bloks or boulders. Along the manuscript there is reference to different particle sizes and one wonders exactly what was studied. This has implications in the sediment transport formulas to be used. Nandasena's formulation is correct and commonly applied. By assuming rolling as the main transport mechanism you imply the smallest wave heights were responsible for transport against gravity...I have some concerns regarding this. Certainly some transport was by suspension and saltation and those values would significantly increase the wave height necessary for movement. This need to be much more discussed in the manuscript. Fiannly, I missed a section with the hydrodynamic regime (mean wave height, storm wave base, period, etc.)...and also a section of provenance of the boulders. You referred to them as granitic but can you establish the original source? Can you please provide some insight on this? This is also very important....to infer travel distances and to be certain of wave height necessary we need to have an idea were the boulders were sourced from. What was the minimum horizontal distance traveled? An the vertical? In my opinion this needs to be added to the manuscript to make the interpretations forwarded even more robust.

Author's reply:

We sincerely thank the reviewer for the insightful and constructive comments regarding the characterization of particle sizes, transport mechanisms, and the hydrodynamic regime. These points are crucial for improving the robustness and clarity of our interpretations. Accordingly, we have thoroughly revised the manuscript to address all these aspects in detail.

First, we clarify that our analysis focuses exclusively on boulders—particles with diameters exceeding 50 cm (Lines 202–203) —which are consistent with the scale commonly used in storm-wave boulder studies (e.g., Nandasena et al., 2011; Etienne and Paris, 2010). To ensure that our analysis reflects wave-transported materials, we excluded irregular, angular, or exceptionally large boulders that could be of glacial origin or reworked from subglacial deposits, retaining only rounded to sub-rounded clasts with clear morphological evidence of wave reworking. For each beach ridge, the largest boulders are interpreted as indicators of the extreme wave conditions required for ridge formation, reflecting the necessary wave heights to mobilize and emplace these boulders. However, as some outsize boulders may result from non-wave processes, we used the second-largest boulder from each ridge—after excluding outliers—to estimate extreme wave heights. This approach balances the need to identify the upper hydrodynamic limits while avoiding potential overestimation due to isolated, non-representative blocks (Lines 203–213).

Second, following the reviewer's advice, we refined our discussion of transport mechanisms. Rather than assuming rolling as the sole motion mode, we applied Nandasena's equations to calculate the critical flow velocities for sliding, rolling (overturning), and saltation, and converted these velocities into corresponding wave heights. For the four most prominent ridges (A, E, H, M), the minimum wave heights required to initiate sliding range from 3.27–4.09 m, whereas rolling would require 6.26–8.90 m. For less developed ridges, the corresponding values are lower (sliding: 1.42–3.27 m; rolling: 2.48–6.26 m). Saltation of meter-scale boulders would require unrealistically large wave heights (> 26 m) and is therefore considered implausible, although this process may occur for smaller clasts (Lines 214–227).

Third, to better constrain the hydrodynamic context, we incorporated both modern observational and modeled wave data from the Ross Sea and the Terra Nova Bay Polynya (TNBP). The PIPERS project, conducted in the seasonal sea-ice zone of the Ross Sea, recorded significant wave heights exceeding 2 m during strong katabatic wind events in TNBP (April 2017) using SWIFT drifters (Ackley et al., 2020). Furthermore, Herman (2024) simulated ten strong wind events (in September–October between 2016 and 2021) in TNBP, yielding typical significant wave heights of around 3 m, and showing that under ice-free conditions, wave heights could exceed 5 m. In the broader Ross Sea region, wave buoy data from the 2017 PIPERS campaign documented several extreme events with mean significant wave heights reaching 6.3 m, deep-water heights exceeding 7 m, and wave periods of 6–10 s (Montiel et al., 2022; Kohout et al., 2014). These modern observations and simulations provide independent support for our reconstructed Holocene wave conditions, suggesting that the nearshore critical wave heights (>3 m, occasionally approaching 6 m) required to initiate boulder movement in the TNBP are realistic within the present hydrodynamic regime. Considering that the early Holocene was characterized by warmer climatic conditions, reduced sea-ice cover, and enhanced incursions of warm, moisture-laden air masses from the mid-latitude Pacific, it is plausible that wave activity during that period was even more energetic than today, facilitating the formation of the raised boulder beaches observed on Inexpressible Island (Lines 271–298).

Fourth, we added a new subsection, Wave height calculations, where we further quantify the nearshore hydrodynamics. Based on conditions in TNBP and the Ross Sea, a deep-water wavelength of L_0 = 56 m (T_0 = 6 s) yields an estimated wave run-up ($R_{2\%}$) ≈ 3.19 m using the Stockdon formula. For representative storm parameters, the calculated breaking depth is approximately 4.9 m, and the corresponding breaking wave height (H_b) ≈ 8.3 m (see Methods). These results indicate that during extreme events, waves were depth-limited and breaking in the nearshore, conditions that maximize boulder-mobilizing forces. Based on these parameters, we selected the formula used for wave-height calculations and also report the associated wave periods required to initiate boulder motion (Lines417–452).

Finally, regarding boulder provenance, petrographic analysis of small rock fragments collected from the ridges confirmed a granitic composition, identical to boulders in modern beaches and the nearshore zone of Inexpressible Island. Considering the regional deglaciation history, these boulders likely originated from local bedrock that was submerged and reworked by waves following ice retreat. Subsequent glacio-isostatic uplift progressively elevated these former nearshore deposits, producing the observed stepped sequence of raised beach ridges. Consistently, ^{10}Be exposure ages show an offshore-to-onshore younging trend: older ages correspond to more distant ridges, while the youngest ages occur at the innermost ridge, indicating progressive emergence of former subaqueous deposits as sea level fell (Yıldırım et al., 2024).

Given this local source, horizontal transport distances were likely short, with most clasts moved on or near the shoreface prior to uplift (Lines 206–211).

Together, these revisions substantially strengthen the physical basis of our interpretations. The revised manuscript (Results: Reconstruction of storm events; Discussion: The Early-Mid Holocene extreme waves and Climate–Ocean Coupling) now clearly defines the analyzed materials, clarifies the hydrodynamic regime and incorporates modern hydrodynamic constraints—ensuring a comprehensive and physically consistent interpretation of the storm-wave processes responsible for the formation of raised boulder beaches on Inexpressible Island.

Revision:

…

We estimated the wave heights required to initiate the transport of the largest wave-movable granite boulders preserved on the gravel beach ridges of Inexpressible Island (Supplementary Tables 7, 8). As in our methodological approach, we excluded angular, oversized boulders with low roundness and dimensions markedly greater than the surrounding clast population, which may have been reworked from glacial till rather than wave-transported. Petrographic analysis confirms that the ridge-forming clasts are predominantly granitic and compositionally identical to those observed in the modern beaches and nearshore zone, suggesting a local bedrock source that was reworked by coastal processes following ice retreat. This local provenance implies limited horizontal transport distances, meaning that the calculated wave heights primarily reflect the hydrodynamic energy necessary to mobilize in-situ or nearshore boulders rather than longshore-drifted material. To further minimize the influence of outliers, wave energy estimates for each ridge were based on the second-largest boulder rather than the absolute largest, providing a conservative and robust constraint on the hydraulic conditions necessary for ridge formation.

Using established hydrodynamic equations, we calculated the critical flow velocities at the wave breaking point for three transport modes—sliding, rolling (overturning), and saltation—which were then converted to wave heights using formulas can be found in Methods. The results reveal a clear relationship between ridge relief, boulder size, and the calculated wave heights required for sliding and rolling transport modes. For the four most prominent ridges (A, E, H, and M), minimum wave heights needed to initiate sliding range from 3.27 to 4.09 m, whereas rolling requires 6.26–8.90 m. Less developed ridges generally require lower thresholds, with sliding initiated at 1.42–3.27 m and rolling at 2.48–6.26 m. For meter-scale boulders, saltation would demand unrealistically large waves—exceeding 26 m for some examples—which is far above the 6–9 m maximum significant wave heights documented in the Ross Sea[43]; thus, its contribution to the transport of the largest clasts is likely minimal, though it may occur for smaller material under certain conditions. Overall, these calculations suggest that the formation of the most prominent ridges was likely driven by one or more exceptionally energetic storm events, producing significant wave heights exceeding 3 m and, in some cases, approaching 6 m, followed by repeated high-energy reworking that contributed to clast redistribution and ridge progradation.

…

…

Based on boulder size and beach ridge morphology, we infer that between approximately 8 and 5 ka, the coastal environment of Inexpressible Island experienced a prolonged period of enhanced storminess, during which multiple exceptionally energetic storm episodes generated significant wave heights exceeding 3 m and occasionally approaching 6 m, accompanied by several moderate events around 2 m. Rather than representing isolated extreme incidents, these high-energy events likely occurred repeatedly over millennial timescales, with cumulative wave impacts driving boulder entrainment, clast redistribution, and progressive ridge progradation. The estimated wave heights thus represent the critical nearshore conditions necessary to initiate boulder motion under sustained extreme storm regimes. To evaluate the plausibility of these reconstructed values, we compared them with modern observational and modeled data from the Ross Sea and the Terra Nova Bay Polynya (TNBP). The PIPERS project (Polynyas, Ice Production, and seasonal Evolution in the Ross Sea), conducted in the seasonal sea-ice zone, recorded significant wave heights exceeding 2 m during strong katabatic wind events in the Terra Nova Bay Polynya (April 2017) using SWIFT drifters[35]. Numerical simulations of ten intense wind events occurring between 2016 and 2021 (in September–October) in the same region produced typical significant wave heights of around 3 m, with maximum values exceeding 5 m under ice-free conditions[36]. In the broader Ross Sea region, wave buoy data from the 2017 PIPERS campaign documented several extreme events, with mean significant wave heights reaching 6.3 m, deep-water wave heights exceeding 7 m, and wave periods of 6–10 s[43,44].

These modern observations and simulations provide an important reference for evaluating our reconstructed Holocene wave heights. For ridges composed of smaller clasts, the calculated significant wave heights are around 2 m, whereas the largest ridges, containing meter-scale boulders, require waves exceeding 3 m, and up to ~6 m under rolling transport conditions, to initiate motion. Although these values are higher than those observed during most modern extreme events in the TNBP, they remain within the upper range of simulated ice-free scenarios, indicating that our estimates are hydrodynamically realistic. Importantly, these reconstructions represent millennial-scale storm regimes that were far rarer and more energetic than short-term modern events, thus providing a longer-term perspective on extreme wave activity in the Ross Sea. The close correspondence between reconstructed and modeled magnitudes further suggests that wave energy during the early Holocene was more vigorous than at present, reflecting a period of reduced sea-ice cover, expanded open-water fetch, and intensified atmosphere–ocean coupling.…

…

In the Ross Sea region, wave buoy data collected during the 2017 PIPERS project revealed several significant wave events. In late June, a major wave event recorded an average SWH of approximately 6.3 m, with deep-water wave heights exceeding 7 m and marginal ice zone wave heights surpassing 5 m. During events where SWH exceeded 4 m, wave periods ranged from 6 to 10 second[43,44] These observations are consistent with the predictions from the WAVEWATCH III (WW3) model simulations presented by ref[79]. Based on these data, we assume a representative deep-water significant wave height (H_0) of 7 m and a wave period (T_0) of 6 s during major storm events affecting the western coast of Inexpressible Island. Using the deep-water wave length equation $L_0 = \frac{gT^2}{2\pi}$, the resulting wave length is approximately 56 m. Field measurements indicate an average beach slope (tan θ) of 10° in the southern bay area.

Applying these parameters to the wave run-up formula[80]:

$$R_{2\%} = 1.1\left[0.35 \tan\theta \sqrt{(H_0 L_0)} + 0.5\sqrt{(H_0 L_0)(0.563 \tan^2\theta + 0.004)}\right],$$

we estimate a maximum run-up height (R_2%) of approximately 3.19 m. This value exceeds the relief of the raised beaches in the area, which ranges between 0.5 and 3.09 m, indicating that storm waves of this magnitude were indeed capable of overtopping and reworking the beach surface, leading to the formation of storm surge-related sedimentary deposits.

To further determine whether wave breaking occurred in the nearshore zone, we analyzed the relationship between the wave height and water depth at the breaking point. The breaking wave height H_b was estimated using the empirical equation proposed by Sunamura and Horikawa[81]:

$$\frac{H_b}{H_0} = (\tan\beta)^{0.2}\left(\frac{H_0}{L_0}\right)^{-0.25}$$

where β represents the average slope of the nearshore profile from the shoreline to a depth of 20 m.

The corresponding water depth at the breaking point h_b was estimated using the empirical relationship provided by Collins[82]:

$$\frac{H_b}{h_b} = 0.72 + 5.6\tan\beta$$

The calculated wave height at the breaking point (8.32 m) and corresponding water depth (4.88 m). This indicates that waves in the nearshore zone of Inexpressible Island were already in a breaking regime, enhancing their capacity to mobilize large boulders through rolling or saltation processes.

Accordingly, the critical wave height required to initiate boulder transport can be estimated based on the critical flow velocity using the equation $u = \delta\sqrt{gH}$, where H is the wave height (in meters), and δ is a wave type parameter. Specifically, when a storm occurs and wave breaking is present, $\delta = 0.5$; when a storm occurs without wave breaking, H is defined as the breaking wave height H_b, and $\delta = 1$; for tsunami conditions, $\delta = 2$[23]. In this study, δ is set to 0.5, reflecting the assumption of wave breaking during storm events. Based on the boulder size, density, and elevation, we then calculated the wave period conditions necessary to initiate boulder motion using the following equation[83]:

$$T = \frac{2}{g}\left(\frac{\rho_w}{\rho_s - \rho_w}\right)\left(\frac{C_d}{tan\,\theta}\right)\left(\frac{h_{clast}}{D_i}\right)u$$

where h_{clast} is the vertical displacement of the boulder (m), defined as the difference between the ridge relief and the boulder's height (short axis), and D_i is the intermediate diameter of the transported clast (m).

…

…Petrographic analysis confirms that the ridge-forming clasts are predominantly granitic and compositionally identical to those observed in the modern beaches and nearshore zone, suggesting a local bedrock source that was reworked by coastal processes following ice retreat. This local provenance implies limited horizontal transport distances, meaning that the calculated wave heights primarily reflect the hydrodynamic energy necessary to mobilize in-situ or nearshore boulders rather than longshore-drifted material. …

5- Storminess vs Extreme events - based on the point above, I consider that storminess and not single events are likely the cause of the boulder ridges described in the manuscript. This result should be even further stressed by the authors as it is the key point of this work.

Author's reply:

We thank the reviewer for this insightful comment. We fully agree that the raised boulder ridges most likely reflect the cumulative influence of repeated high-energy storm events rather than a single extreme wave impact. In response, we have revised both the Results and Discussion sections to better emphasize this interpretation.

Specifically, we clarified that the ridge formation was associated with exceptionally energetic storms followed by multiple episodes of reworking, highlighting the long-term geomorphic imprint of enhanced storminess. We also strengthened the discussion of how this pattern reflects a period of increased storm frequency and intensity under early–mid Holocene climatic conditions (Lines 224–227; 274–278; 293–298).

These revisions make it clearer that the boulder ridges represent the cumulative response to sustained storminess over centennial to millennial timescales, rather than the product of isolated extreme events, which we now stress as one of the key findings of this study.

Revision:

…Overall, these calculations suggest that the formation of the most prominent ridges was likely driven by one or more exceptionally energetic storm events, producing significant wave heights exceeding 3 m and, in some cases, approaching 6 m, followed by repeated high-energy reworking that contributed to clast redistribution and ridge progradation.

…

…

…Rather than representing isolated extreme incidents, these high-energy events likely occurred repeatedly over millennial timescales, with cumulative wave impacts driving boulder entrainment, clast redistribution, and progressive ridge progradation. The estimated wave heights thus represent the critical nearshore conditions necessary to initiate boulder motion under sustained extreme storm regimes. …

…Importantly, these reconstructions represent millennial-scale storm regimes that were far rarer and more energetic than short-term modern events, thus providing a longer-term perspective on extreme wave activity in the Ross Sea. The close correspondence between reconstructed and modeled magnitudes further suggests that wave energy during the early Holocene was more vigorous than at present, reflecting a period of reduced sea-ice cover, expanded open-water fetch, and intensified atmosphere–ocean coupling.

…

Overall, a very nicely written manuscript that presents a very interesting case study. In my opinion the manuscript should be accepted for publication after minor changes are made.

Author's reply:

We sincerely thank the reviewer for the thoughtful and encouraging comments, and for taking the time to carefully evaluate our manuscript. We greatly appreciate the recognition of our objectives, figures, and overall approach. Following the reviewer's suggestions, we have made careful revisions to improve the clarity and transparency of the manuscript, including clarifying the study's geographical context, topographic measurements, age data, boulder characteristics and provenance, and the interpretation of storm-wave processes. We hope that these revisions adequately address the reviewer's concerns and improve the overall presentation of our work, and we are grateful for the reviewer's constructive guidance that has helped strengthen the manuscript.

<End of Summary of Changes>

Summary of Changes

The authors would like to thank the reviewers for their constructive comments and kind support. We provide below a detailed account on the changes that we have made in response to the comments that the reviewers have raised. We have marked the corresponding changes in the revised version in *red* color.

Response to Reviewer #4:

I, first, want to commend the authors on the most comprehensive and thorough responses to reviewer comments that I have ever seen. This is the first version of the manuscript that I am reviewing and found the analytical methods appropriate, the results well presented, and the discussion convincing and backed by results. By combining beach ages, granulometry of beach clasts, wave modeling, and published proxy datasets, the authors present a compelling case for heightened storminess in the early Holocene linked with decreased sea ice and initial deglaciation of the coastal environment.

A couple comments/questions that I have relate to the choice of using the second to largest clast to estimate wave energy and be careful to acknowledge that individual beaches could be formed by a single or multiple storm events. Why not use mean clast size or incorporate uncertainties by using the range of clast sizes?

Authors' reply:

We thank the reviewer for this insightful suggestion. We agree that using the distribution of clast sizes provides a more representative estimate of wave energy than relying solely on the second-largest clast. We have now expanded our analysis to include percentile-based calculations (P5, P25, P50, P75, P95) for each beach ridge, which better captures the range of wave conditions during beach formation.

We sincerely thank Reviewer for the generous and encouraging assessment of our work, and for the thoughtful comments that have further strengthened the manuscript.

Regarding the choice of clast size for wave energy estimation, we fully agree with the reviewer's suggestion. In the revised manuscript, we have replaced the single second-largest clast approach with a percentile-based analysis (P5, P25, P50, P75, and P95) applied to the full boulder size distribution of each beach ridge. P50 provides a robust estimate of typical formative wave conditions, less sensitive to outliers than the second-largest clast, while P95 captures the extreme end of wave energy experienced by each ridge. We have also explicitly acknowledged that individual ridges were likely constructed through repeated storm events of varying intensity rather than single catastrophic episodes, consistent with the reviewer's caution. The broad wave height range implied by the P5–P95 distribution (sliding: 0.38–4.19 m; rolling: 0.62–8.90 m) supports this interpretation and is discussed accordingly. These revisions are reflected in the updated Supplementary Figure 3, and the revised text (lines 228–246, marked in red in the manuscript).

Revision:

…

Using established hydrodynamic equations, we estimated the critical wave heights required to initiate boulder transport via sliding, rolling/overturning, and saltation/lifting across the full size distribution of boulders on each ridge. Wave parameters were calculated for the P5–P95 percentiles, where P50 represents typical formative conditions and P95 captures extreme events (Supplementary Fig. 3). The results reveal systematic relationships between ridge morphology, boulder size, and wave energy requirements (Fig. 3; Supplementary Fig. 3). For sliding, median boulders (P50) require wave heights of 0.73–2.56 m across all ridges, while the largest boulders (P95) demand 1.19–4.19 m. Rolling/overturning requires substantially higher energy: P50 thresholds range from 0.96–5.08 m, and P95 thresholds reach 4.62–8.90 m for the most developed ridges (A, E, H, M, N). Saltation/lifting would demand unrealistically large waves for meter-scale boulders (>26 m for some P95 cases), indicating a negligible role in transporting the largest clasts.

The broad size distribution within each ridge argues against formation by a single extreme event and instead reflects cumulative deposition from multiple storms of varying intensity. Smaller boulders (P5–P25) record frequent, lower-energy storms, while the largest clasts (P75–P95) preserve rare, extreme events. The notable similarity of P95 values among the most prominent ridges (sliding/rolling wave heights, with corresponding P95 clast dimensions (L×I×S) in parentheses: A: 4.09/8.90 m (125.0×90.0×40.0 cm), E: 4.19/8.16 m (130.0×90.0×50.0 cm), H: 2.84/4.95 m (180.0×60.0×40.0 cm), N: 2.87/4.62 m (90.0×60.0×45.0 cm) suggests comparable extreme storm intensities across different phases of relative sea-level change. Taken together, ridge construction was driven by a spectrum of storm events, with typical conditions of 1–3 m significant wave height and episodic extremes reaching 4–9 m.

…

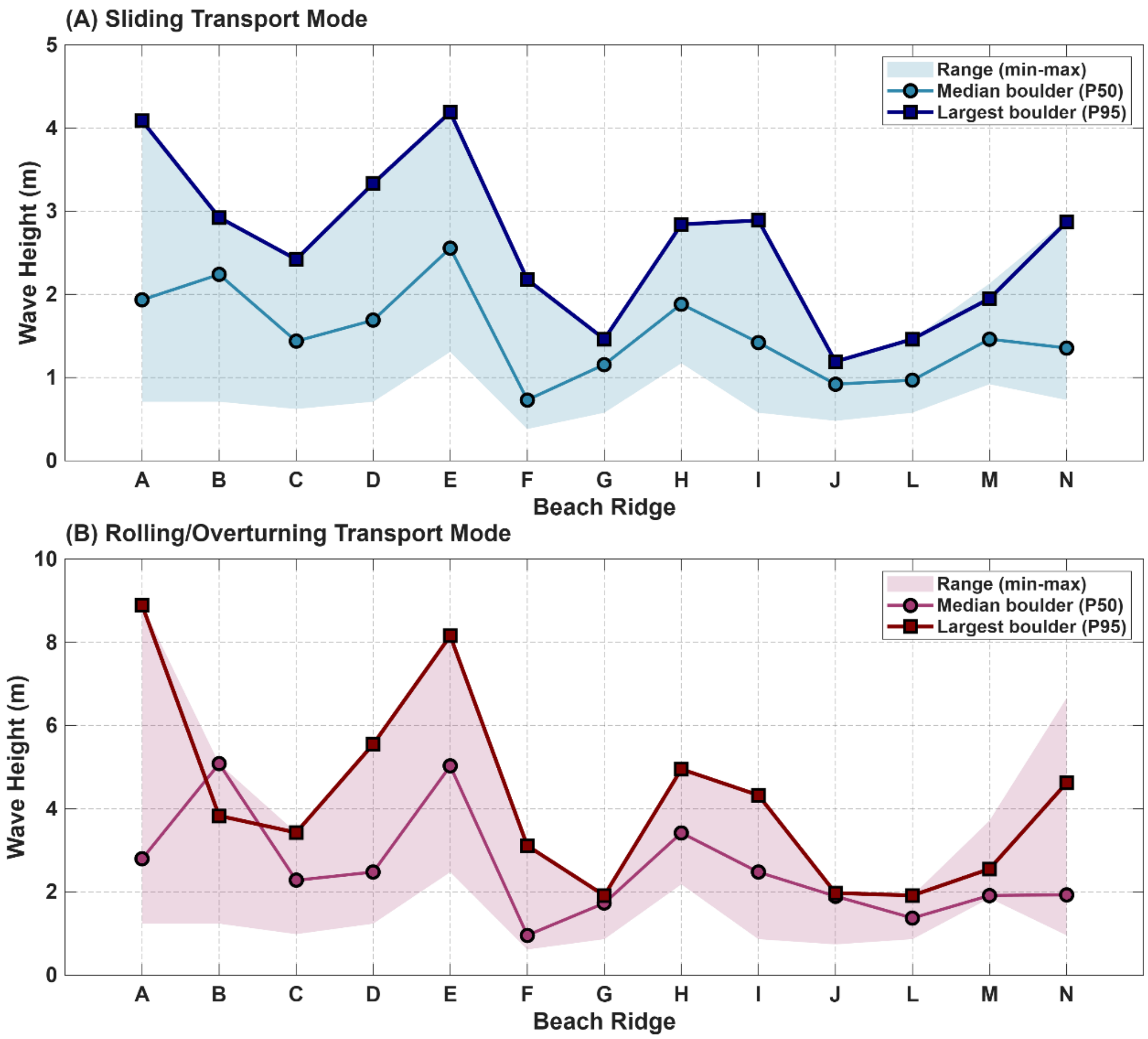

Supplementary Fig. 3.

Critical wave heights required to initiate boulder transport via sliding (A) and rolling/overturning (B) for each beach ridge (A–N) at the southernmost bay of Inexpressible Island. Circles and squares denote the median (P50) and largest (P95) boulder size classes, respectively, and shaded areas represent the full range (min–max) of wave heights across the P5–P95 size distribution. Beach ridges K and O are excluded due to snow cover during field measurement.

Response to Reviewer #5:

I enjoyed reading the revised manuscript and review/response, the paper is interesting and I think it is worth publishing. I have some comments on how it can be improved, with a focus on the 10Be exposure ages, specifically how they have been interpreted and applied:

1. The authors describe the 10Be ages as "effective" exposure ages, do they mean "apparent" exposure ages, assuming one period of continuous exposure and no erosion? If so, this should be made clearer in the text.

Authors' reply:

We thank the reviewer for this important clarification. We have replaced "effective exposure ages" with "apparent exposure ages" throughout the manuscript to adopt the standard terminology (line 167). This term refers to ages calculated assuming a single period of continuous exposure without erosion correction, representing the raw modeled output prior to any geological corrections. The potential departures from these assumptions, including nuclide inheritance and the effect of GIA-driven elevation change on cosmic ray production rates, are addressed in our response to Comment 2 below, where we describe the corrections applied to refine the final interpreted ages.

Revision:

…Apparent exposure ages from ridges A, E, H, and O are 9.07 ± 1.20 ka, 8.83 ± 1.10 ka, 3.61 ± 0.75 ka, and 5.22 ± 0.90 ka, respectively (1σ external uncertainties; see Methods).…

2. The authors state in their revised text (line 190 - 192) that:

"However, when the external uncertainties of both dating methods are taken into account, their age ranges show substantial overlap. This indicates that the apparent offset is within the expected bounds of analytical uncertainty, and both dates likely reflect the same event, without any fundamental contradiction."

Given that we are comparing different dating methods and the overlap of external uncertainties is used to argue that the methods are dating the same event (and in agreement), I think these external uncertainties need to be included in Figure 4. At present, Figure 4 makes it seem like the 10Be ages overestimate the ages of the beaches (vs the 14C ages), as the authors state in the text (and was noted by another reviewer). The authors could argue that the 10Be ages are (at least in part) a little older than 14C ages here due to a small amount of nuclide inheritance due to, for example, 10Be being produced through shallow water, meaning boulders making up the beach may contain a prior signal to beach formation. This might not be how the authors are interpreting the 10Be ages (and they could disagree, of course), but it would be helpful if the paper had a little more info generally on how they have been interpreted. Another consideration on the 10Be ages is a correction for GIA - we know the beaches were formed at lower elevations and have since been uplifted by GIA, how would this impact the resulting exposure ages? Presumably the higher elevation, older ages would be impacted the most, as they have been uplifted the greatest extent (e.g. see Jones et al. (2019) https://doi.org/10.1016/j.quascirev.2019.03.012). Alternatively, if the authors are assuming no elevation change impact on exposure ages, this needs to be stated within the text (apologies if I have missed it!).

Authors' reply:

We thank the reviewer for these constructive and detailed comments. We have addressed each point as follows.

We agree that external uncertainties should be shown in Figure 4, and the figure has been revised accordingly to display 1σ external uncertainties for all four measured ^{10}Be exposure ages (both raw and inheritance-corrected). We have also expanded the discussion of the apparent age offset between the ^{10}Be and ^{14}C ages in the revised text (lines 169–201). The slightly older apparent ^{10}Be ages are interpreted as reflecting a small inherited ^{10}Be component accumulated prior to final beach deposition, most plausibly through shallow-water cosmogenic production or intermittent subaerial exposure during boulder transport — consistent with the mechanism suggested by the reviewer. Based on ridges A, E, and O, we estimate a mean inheritance of ~1049 yr (95% CI: 0 to +2850 yr); a sensitivity analysis across colonization lag values of 0–1500 yr yields inheritance estimates ranging from ~550 to ~2050 yr. After applying this correction, the inheritance-corrected ^{10}Be ages align closely with the ^{14}C-based elevation–age trend, with residual offsets falling within the combined external uncertainties of both methods.

Regarding the GIA correction, we apologise that this was not clearly stated in the original manuscript. GIA corrections have been applied to all four ^{10}Be samples using the iceTEA tools (Jones et al., 2019) with the ICE-6G_C GIA model (Peltier et al., 2015) and the LSDn time-dependent scaling scheme (Lifton et al., 2014). The resulting age offsets are small (10 – 140 yr, ≤1.5% of uncorrected ages), reflecting the modest isostatic uplift history of Inexpressible Island during the Holocene. Although these corrections are negligible relative to measurement uncertainties, they are incorporated for completeness, and GIA-corrected ages are reported in Supplementary Table 5.

Revision:

…These represent raw modeled outputs assuming a single period of continuous exposure, prior to geological corrections described below. GIA corrections were applied to all four samples using iceTEA45 with the ICE-6G model. The resulting age offsets are small (10–140 yr, ≤1.5% of uncorrected ages) and negligible relative to measurement uncertainties, but are incorporated for completeness; GIA-corrected ages are reported in Supplementary Table 5.

To better constrain the formation ages of the raised beaches, we compiled 48 published AMS 14C ages from the South Bay and Southernmost Bay of Inexpressible Island (Fig. 4; Supplementary Fig. 4; Supplementary Table 6), derived from storm beach organics, seal skin overlying marine sediments, penguin remains in ornithogenic soils, bulk sediments containing penguin guano, and in situ marine shells. These different material types provide complementary chronological constraints: penguin and seal remains yield minimum limiting ages for beach emergence, since these animals only colonize land surfaces after they emerge above sea level; in situ marine shells provide maximum limiting ages, as these organisms lived when the beach was still submerged; and organic materials within well-preserved beach stratigraphy relatively closely approximate true depositional ages.

Comparison of the GIA-corrected 10Be ages with the compiled AMS 14C dataset reveals that samples appear slightly older than AMS 14C ages from nearby beach deposits at comparable elevations, a pattern consistent with a small inherited 10Be component accumulated prior to final deposition through shallow-water production or intermittent subaerial exposure during boulder transport. We note, however, that AMS 14C ages of organic materials from beach deposits also carry a systematic offset relative to true beach formation age, as colonization by penguins and seals necessarily postdates beach emergence.

This colonization lag (~1000 ± 400 yr, estimated from the offset between storm beach organics and the broader AMS 14C dataset at equivalent elevations) was therefore incorporated into the inheritance estimation. Using a weighted regression of GIA-corrected 10Be ages against lag-corrected storm beach organic AMS 14C ages, we estimate a mean inheritance of ~1049 yr (95% CI: 0 to +2850 yr), based on samples from ridges A, E, and O. The wide confidence interval reflects the limited sample size and sensitivity to the assumed colonization lag (Fig. 4); a sensitivity analysis across lag values of 0–1500 yr yields inheritance estimates ranging from ~550 to ~2050 yr. Sample MCB8-1 from ridge H was excluded from this estimate, as it yields an anomalously young apparent age (3.61 ± 0.75 ka) relative to both the local 14C chronology and the other 10Be samples, likely reflecting post-depositional surface disturbance. After applying the inheritance correction, the 10Be ages from ridges A, E, and O show substantially improved agreement with the AMS 14C chronology, and residual offsets fall within the combined external uncertainties of both methods (Fig. 4).

…

Fig. 4: Age–elevation relationship of storm-built raised beaches at Southernmost Bay on Inexpressible Island, Antarctica. Navy blue diamonds show Bayesian fusion formation ages for ridges A–O with 1σ uncertainty ranges; the solid regression line has $R^2 = 0.993$. 10Be exposure ages are listed in Supplementary Table 5; AMS 14C ages from previously published studies (ref7,8,40,41,83-85) are listed in Supplementary Table 6; Bayesian fusion formation ages for individual ridges are listed in Supplementary Table 7.

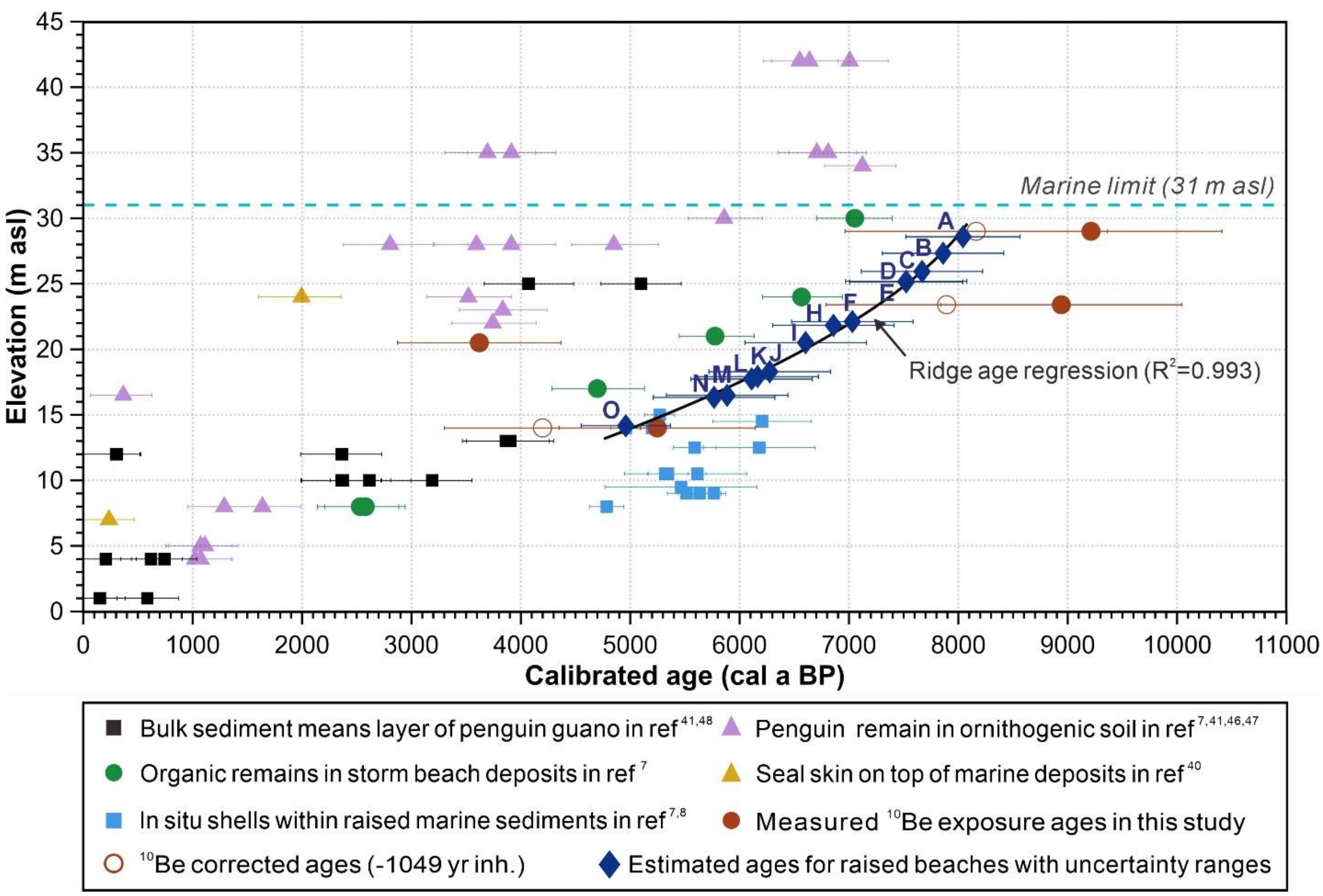

3. I think the description of the integrated age-elevation framework that the authors are using needs i) a bit more detail in the main text, and ii) a new, very detailed section in the methods.

To demonstrate why I think these are needed, I go through the following example. The authors state on lines 193-194 “For ridges with direct ^{10}Be or ^{14}C ages at the corresponding elevations, these were directly applied”. The age of formation for Ridge A is “ between 8400 and 7300 yrs BP”, however, Ridge A has a 10Be age of 9.07 ± 1.20 ka, so why is the age of formation not ~9 ka? With Ridge A, are the authors using the 10Be age and 14C ages to get to this age of 8400 - 7300 yrs BP? If so, this is unclear as written. If this is the case, how are the two constraints used and weighted? How does this vary for each beach ridge? I could be missing something here, but I think the paper is lacking a detailed section on this important part of the study.

Authors’ reply:

We thank the reviewer for this detailed and constructive comment. We fully agree that the age estimation framework was insufficiently described in the original manuscript, and we have taken this opportunity to substantially revise and expand both the main text and the Methods section in response. We hope the revised presentation addresses the reviewer's concerns and makes the full procedure transparent and reproducible.

Regarding the specific example of Ridge A: the ^{10}Be exposure age of 9.07 ± 1.20 ka is the raw measured age, which was combined with the ^{14}C ages from comparable elevations within the integrated age estimation framework to yield the formation age estimate. The previously reported range of "8400–7300 yr BP" therefore reflects this combined constraint rather than the raw ^{10}Be age alone, and we acknowledge that this was not clearly explained in the original manuscript. We have revised the relevant passage in the main text (lines 202–212; 425–458) to make this explicit.

More broadly, following the reviewer's suggestion, we have significantly strengthened the description of the age estimation framework in two ways.

In the main text, we have added an explicit description of the Bayesian fusion approach (lines 202–212), explaining that formation ages for individual ridges are estimated by combining three sources of chronological information: (i) inheritance-corrected ^{10}Be exposure ages; (ii) colonization-lag-corrected storm beach organic ^{14}C ages, integrated through a joint weighted regression against ridge elevation; and (iii) a distance-from-shoreline auxiliary regression, introduced as an independent geometric constraint for ridges lacking direct isotopic dating control. This third constraint was not present in the original manuscript and was added specifically. By exploiting the strong geometric relationship between distance from shore and ridge age, this auxiliary regression provides a meaningful independent estimate for every ridge while being assigned a conservative uncertainty of 800 yr (1σ) to prevent it from dominating the posterior where isotopic constraints exist. Stratigraphic bounds from in situ marine shells (upper age limits) and penguin/seal remains (lower age limits) are applied as hard constraints after fusion. The relative contribution of each data source therefore varies by ridge: for ridges A, E, and O, all three constraints are combined; for ridges lacking direct isotopic dating, the formation age is derived from the regression and distance-based auxiliary constraint alone.

In the Methods section (lines 425–455), we have added a new dedicated subsection ("Bayesian fusion chronology for beach ridge formation ages") describing the full procedure step by step, including: (i) the colonization lag correction applied to storm beach organic ^{14}C ages and the derivation of the elevation–age reference curve; (ii) the estimation and propagation of ^{10}Be inheritance uncertainty; (iii) the joint inverse-variance-weighted second-order polynomial regression fitted to the

combined corrected ^{14}C and ^{10}Be dataset; (iv) the distance-from-shoreline auxiliary regression and its uncertainty assignment; (v) the Bayesian fusion step, including the inverse-variance-weighted posterior age formula and its uncertainty; (vi) the application of stratigraphic hard constraints and the associated uncertainty scaling; and (vii) a sensitivity analysis across colonization lag values of 0, 500, 1000, and 1500 yr. We hope this revised and expanded Methods section addresses the reviewer's concerns, and we remain open to further clarification if needed.

Revision:

…Formation ages for individual ridges were estimated using a Bayesian fusion approach combining inheritance-corrected ^{10}Be ages, lag-corrected storm beach organic AMS ^{14}C ages, and a distance-from-shoreline auxiliary regression for ridges lacking direct dating control (see Methods for full details). Stratigraphic bounds from in situ marine shells (upper age limit) and penguin/seal remains (lower age limit) were applied where available. Based on this framework, ridge A (~29 m asl, ~225 m from shore) formed at approximately 8.0 ± 0.5 ka, ridge E (~25 m asl, ~191 m from shore) at ~7.5 ± 0.5 ka, ridge H (~22 m asl, ~156 m from shore) at ~6.9 ± 0.6 ka, ridge M (~17 m asl, ~113 m from shore) at ~5.9 ± 0.6 ka, and ridge O (~14 m asl, ~87 m from shore) at ~5.0 ± 0.4 ka. Together, these results indicate that prominent beach ridge formation on Inexpressible Island occurred episodically during the early to middle Holocene at roughly millennial intervals, driven by a combination of relative sea-level fall and episodic high-energy coastal processes.

…

…

Bayesian fusion chronology for beach ridge formation ages

Formation ages for individual beach ridges were estimated using a multi-source Bayesian fusion framework implemented in MATLAB, integrating inheritance-corrected ^{10}Be exposure ages, colonization-lag-corrected storm beach organic AMS ^{14}C ages, and a distance-from-shoreline auxiliary regression.

Colonization lag and inheritance correction. Storm beach organic AMS ^{14}C ages were corrected for colonization lag by adding 1000 yr (1σ: 400 yr) to each raw age. A weighted linear regression of these lag-corrected ages against ridge elevation yielded an elevation–age reference curve. ^{10}Be inheritance was estimated as the residual between each GIA-corrected ^{10}Be age and the reference curve prediction at the corresponding elevation; the mean residual across ridges A, E, and O was subtracted from all ^{10}Be ages. The combined inheritance uncertainty $\boldsymbol{\sigma_{inh}} = \sqrt{\boldsymbol{\sigma_{\Delta}^2} + \boldsymbol{\sigma_{lag}^2}}$, where $\boldsymbol{\sigma_{\Delta}}$ is the standard deviation of the three residuals, was propagated in quadrature with individual ^{10}Be measurement uncertainties.

Joint weighted regression. A second-order polynomial was fitted to the combined lag-corrected AMS ^{14}C and inheritance-corrected ^{10}Be ages as a function of ridge elevation using inverse-variance weighting. The regression coefficient covariance matrix was retained for uncertainty propagation, and $\boldsymbol{\sigma_{resid}}$ and $\boldsymbol{\sigma_{lag}}$ were added in quadrature to yield per-ridge regression age uncertainties.

Distance-from-shoreline auxiliary constraint. An unweighted linear regression of elevation-based age estimates against distance from the modern shoreline provided an independent geometric constraint for every ridge, assigned a conservative uncertainty of 800 yr (1σ) to prevent it from dominating the posterior where isotopic constraints exist.

Bayesian fusion and stratigraphic constraints. For each ridge, the posterior formation age was computed as the inverse-variance-weighted mean of up to three estimates — the regression-based age, the inheritance-corrected ^{10}Be age (ridges A, E, and O only), and the distance-based auxiliary age:

$$t_{post} = \frac{\sum_i w_i \mu_i}{\sum_i w_i}, \sigma_{post} = \left(\sum_i w_i\right)^{-1/2}, w_i = \frac{1}{\sigma_i^2}$$

where μ_i and σ_i are the age estimate and 1σ uncertainty of the i-th source, respectively. Stratigraphic bounds were then applied as hard constraints: in situ marine shell ages within ±1 m elevation served as upper age limits; penguin and seal remain ages within ±1 m served as lower limits. Where the fused age exceeded the shell-derived upper bound, it was capped at that value and the posterior uncertainty scaled by 0.8 to reflect the additional constraint. Where the fused age fell below the minimum age indicated by penguin or seal remains, it was raised to that lower bound with the posterior uncertainty unchanged.

Sensitivity analysis. The full procedure was repeated for colonization lag values of 0, 500, 1000, and 1500 yr, yielding mean inheritance estimates of ~2049, ~1549, ~1049, and ~549 yr respectively (1σ: ~919 yr in all cases). The systematic decrease with increasing lag reflects the correction structure, but the central estimates remain of comparable magnitude across the plausible lag range, supporting the robustness of the preferred correction (lag = 1000 yr; mean inheritance ~1049 yr; 95% CI: 0 to +2850 yr).

…

4. Lastly, I think some info on the new 10Be ages is missing from the Methods section/supplement. In order to reproduce the 10Be concentrations (and ages), we need to know the concentration of the 9Be carrier, as well as the 10Be concentration (or ratio) of procedural blank(s) and how the blank correction was applied. E.g. were the four samples part of a larger batch of samples? How many procedural blanks were used to correct the sample 10Be concentrations, e.g. one blank for a larger batch of samples? Multiple blanks from one batch with a mean blank correction applied? Or some kind of running average procedural blank?

Authors' reply:

We are grateful to the reviewer for this valuable and constructive comment, which has helped us improve the reproducibility of our reported ^{10}Be concentrations and ages. The four samples reported in this study (NYD-MCB1-1, NYD-MCB5-1, NYD-MCB8-1, and NYD-MCB-R1) were processed as part of a single batch of nine samples, with one procedural blank (W-BLANK2) included per batch. A ^{9}Be carrier solution with a concentration of 935.8 μg·g^{-1} was added to each sample (~0.3 g) prior to dissolution. The procedural blank yielded a ^{10}Be/^{9}Be ratio of 0.731 × 10^{-15}, and the measured ^{10}Be/^{9}Be ratios of all four samples were corrected for procedural blank contribution using this single batch blank prior to conversion to ^{10}Be concentrations in quartz. No running average or multi-blank correction was applied. Following the reviewer's suggestion, these details, including the carrier concentration and blank ^{10}Be/^{9}Be ratio, have been added to the revised Methods section (lines 411–417) to ensure full reproducibility.

Revision:

…A ^{9}Be carrier solution with a concentration of 935.8 μg·g^{-1} was added to each sample (~0.3 g) prior to dissolution. The ^{10}Be/^{9}Be ratios were measured by 3MV Accelerator Mass Spectrometer at Xi'an AMS Center, Institute of Earth Environment,

Chinese Academy of Sciences, using the 07KNSTD standard. Samples were processed in batches of nine, with one procedural blank included per batch. The four samples reported in this study (NYD-MCB1-1, NYD-MCB5-1, NYD-MCB8-1, and NYD-MCB-R1) belong to the same processing batch, for which the procedural blank (W-BLANK2) yielded a $^{10}Be/^{9}Be$ ratio of 0.731×10^{-15}. …

Response to Reviewer #6:

I have read the manuscript following the first round of reviews and corrections and I would like to commend the authoring team for their work and effort. The manuscript is, in my option greatly improved.

Below are my my comments in addition to the more detailed ones embedded in the Pdf file:

1) I believe that the approach of reconstructing paleo wave heights undertaken here is reasonable, especially after the more comprehensive workup presented and , in my opinion, addressed the issues raised by Reviewer 1

2) Chronological control- I struggle to easily correlate dated sites with the beach ridges. This needs to be clearly presented on a figure where dates are linked to field photographs. I suggest adding this information to Figure 3A. This way the reader can intuitively understand if- where there are any age reversals and how do they influence interpretations:

Authors' reply:

We sincerely thank the reviewer for this constructive suggestion. In response, we have made several targeted revisions to improve the visual clarity of the chronological control. First, we have added a new panel (Figure 3F) to the existing Figure 3, which presents a topographic cross-section of the beach ridge sequence from inland to seaward, with the four ^{10}Be-dated samples marked directly on their respective ridges (A, E, H, and O) alongside their exposure ages. This panel is accompanied by field photographs of each sampled ridge (Figures 3B–E), allowing the reader to intuitively link the geochronological data to both the morphological position and the field appearance of each ridge. Second, we have updated Figure 4 to include the ridge labels (A–O) at all relevant positions, so that the stratigraphic and chronological relationships are immediately legible. Third, the precise geographic locations of all four ^{10}Be sampling sites, together with all radiocarbon sample locations compiled from previous studies, are presented in Supplementary Fig. 4, which provides a georeferenced spatial overview of the full geochronological dataset at Southernmost Bay. We believe these revisions together comprehensively address the reviewer's concern and make the chronological framework considerably easier to follow.

Revision:

Fig. 3: Field photographs, ^{10}Be sampling sites, and topographic profile of raised beach ridges at Southernmost Bay on Inexpressible Island, Antarctica. (A) Overview photograph of the raised beach ridge sequence along Southernmost Bay, viewed from the southwest, with 15 ridges labeled A–O from inland to seaward. (B–E) Close-up field photographs of ridges A, E, H, and M, illustrating the well-preserved morphology and large, well-rounded boulders characteristic of these ridges. (F)Topographic profile across the beach ridge sequence (A–O), measured along a shore-perpendicular transect. Filled red circles mark the four ^{10}Be sampling sites; annotated values are the measured ^{10}Be exposure ages with 1σ external uncertainties. Precise GPS coordinates of all samples are provided in Supplementary Fig. 4 and Supplementary Table 4.

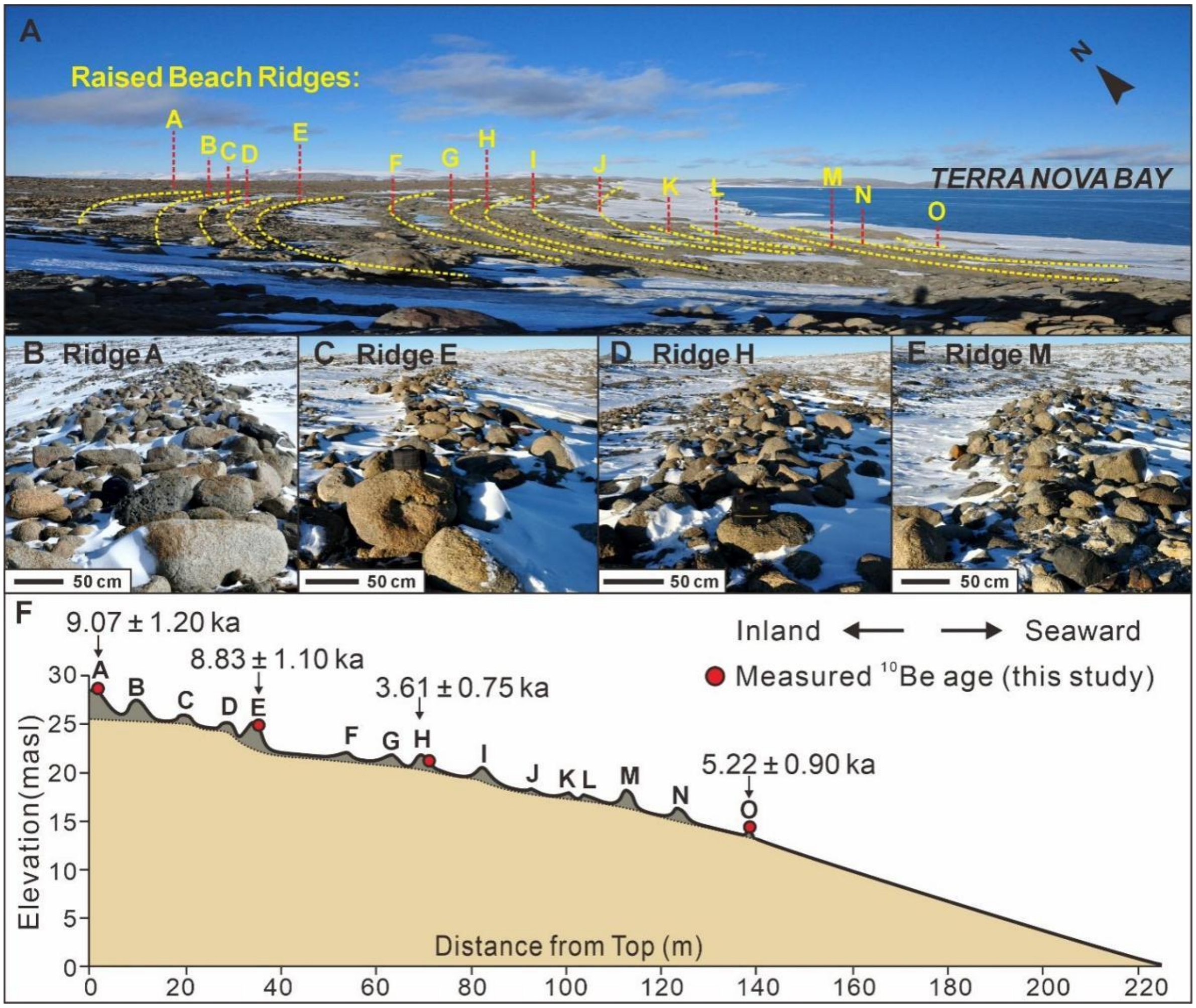

3) Age uncertainties. Authors state that ' external uncertainties' for different dates from the similar locations overlap therefore the dates are statistically representing the same 'event' whereas the +/- ranges mentioned in the text do not seem to overlap at all. This needs to be clearly addressed as I fund it confusing at the very least.

Authors' reply:

We thank the reviewer for this careful observation and apologize for the confusion caused by the imprecise presentation in the original manuscript. To address this concern, we have substantially revised the relevant sections of the manuscript. the raw ^{10}Be exposure ages carry a small inherited ^{10}Be component accumulated prior to final beach deposition, most plausibly through shallow-water cosmogenic production or intermittent subaerial exposure during boulder transport. Based on ridges A, E, and O, we estimate a mean inheritance of ~1049 yr (95% CI: 0 to +2850 yr). After applying this inheritance correction, the corrected ^{10}Be ages do overlap with the ^{14}C-based elevation–age trend within the combined 1σ external uncertainties of both methods, and the apparent contradiction is resolved. We have revised Figure 4 to display 1σ external uncertainties explicitly for all four ^{10}Be ages — both raw and inheritance-corrected — so that the degree of overlap is now directly visible to the reader. The main text has also been revised to clearly distinguish between the raw and corrected ^{10}Be ages and to explain the basis for the inheritance correction (lines 175–201), ensuring that the chronological interpretation is presented in a transparent and internally consistent manner.

Revision:

Fig. 4: Age–elevation relationship of storm-built raised beaches at Southernmost Bay on Inexpressible Island, Antarctica. Navy blue diamonds show Bayesian fusion formation ages for ridges A–O with 1σ uncertainty ranges; the solid regression line has $R^2 = 0.993$. ^{10}Be exposure ages are listed in Supplementary Table 5; AMS ^{14}C ages from previously published studies (ref[7,8,40,41,46-48]) are listed in Supplementary Table 6; Bayesian fusion formation ages for individual ridges are listed in Supplementary Table 7.

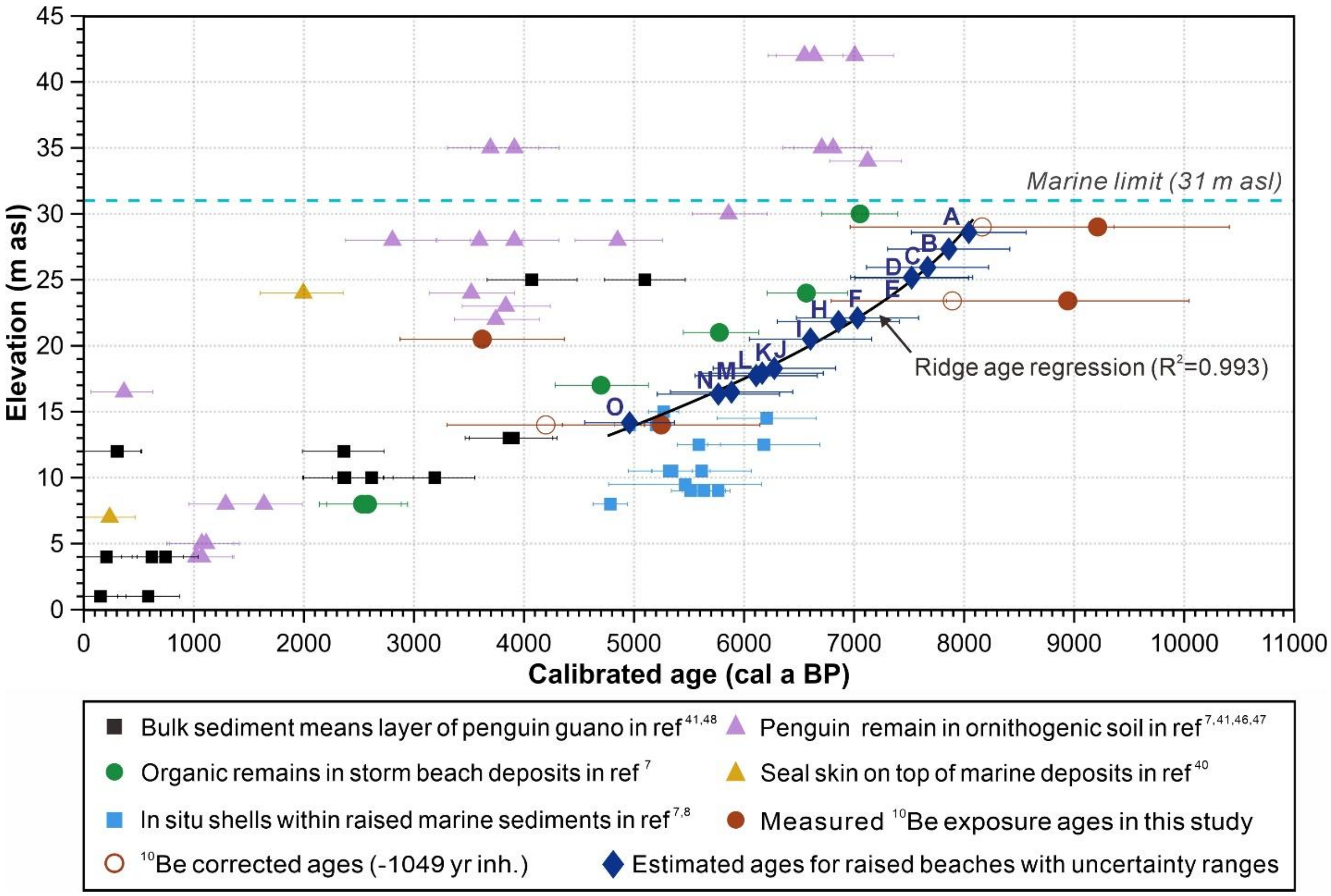

…

To better constrain the formation ages of the raised beaches, we compiled 48 published AMS 14C ages from the South Bay and Southernmost Bay of Inexpressible Island (Fig. 4; Supplementary Fig. 4; Supplementary Table 6), derived from storm beach organics, seal skin overlying marine sediments, penguin remains in ornithogenic soils, bulk sediments containing penguin guano, and in situ marine shells. These different material types provide complementary chronological constraints: penguin and seal remains yield minimum limiting ages for beach emergence, since these animals only colonize land surfaces after they emerge above sea level; in situ marine shells provide maximum limiting ages, as these organisms lived when the beach was still submerged; and organic materials within well-preserved beach stratigraphy relatively closely approximate true depositional ages.

Comparison of the GIA-corrected 10Be ages with the compiled AMS 14C dataset reveals that samples appear slightly older than AMS 14C ages from nearby beach deposits at comparable elevations, a pattern consistent with a small inherited 10Be component accumulated prior to final deposition through shallow-water production or intermittent subaerial exposure during boulder transport. We note, however, that AMS 14C ages of organic materials from beach deposits also carry a systematic

offset relative to true beach formation age, as colonization by penguins and seals necessarily postdates beach emergence. This colonization lag (~1000 ± 400 yr, estimated from the offset between storm beach organics and the broader AMS 14C dataset at equivalent elevations) was therefore incorporated into the inheritance estimation. Using a weighted regression of GIA-corrected 10Be ages against lag-corrected storm beach organic AMS 14C ages, we estimate a mean inheritance of ~1049 yr (95% CI: 0 to +2850 yr), based on samples from ridges A, E, and O. The wide confidence interval reflects the limited sample size and sensitivity to the assumed colonization lag (Fig. 4); a sensitivity analysis across lag values of 0–1500 yr yields inheritance estimates ranging from ~550 to ~2050 yr. Sample MCB8-1 from ridge H was excluded from this estimate, as it yields an anomalously young apparent age (3.61 ± 0.75 ka) relative to both the local 14C chronology and the other 10Be samples, likely reflecting post-depositional surface disturbance. After applying the inheritance correction, the 10Be ages from ridges A, E, and O show substantially improved agreement with the AMS 14C chronology, and residual offsets fall within the combined external uncertainties of both methods (Fig. 4).

…

4) References to till outcrops or local bedrock source are made in order to explain clast lithology and morphology but no information about the two formations are presented- please provide this information to support the claims.

Authors' reply:

We thank the reviewer for this insightful comment. We agree that the original phrasing lacked sufficient geological context. In the revised manuscript, we have added relevant geological background to provide clearer context for the provenance and characteristics of the ridge-forming clasts (lines 117–126).

Revision:

…

During the Last Glacial Maximum, ice inundated Inexpressible Island to possibly 400 m elevation, depositing a thin sheet of matrix-supported diamicton and erratics known as Terra Nova Drift, in which clasts range from angular to glacially moulded with striations[7]. The bedrock of Inexpressible Island consists predominantly of Ordovician Granite Harbor Intrusives and Precambrian metamorphic complexes of the Wilson Terrane[7,8]. Following glacier retreat and isostatic uplift, Holocene raised beaches were cut into Terra Nova Drift or rest directly on bedrock, with beach sediments extending up to about 30 m asl[8]. The deposits are composed mainly of granite and other intrusives, with a minor proportion of metamorphic and volcanic rocks, and their upper topographic limit is marked by a sharp and distinctive boundary where slightly stained, well-rounded pebbles and cobbles contact brown-stained, subrounded to angular, poorly sorted gravel and cobbles of the glacial drift[8].

…

5) Authors address previous reviewers comments by comprehensively presenting modern measurements of wave heights in the region. From what I understand the modern waved heights reach similar amplitudes to the ones postulated to had been responsible for the movement of the largest clasts. If correct, it is making the claim of 'Increased storminess between 5-8ka BP' questionable as a very short met-ocean measurement campaign already provided results that indicate that the storms of

such magnitude can be considered common at present. This is perhaps my main criticism that needs clarification because the whole research hypothesis hinges on that result alone. Furthermore- The authors present a photograph of the modern beach ridge affected by storm processes but it does not appear to be included in the analysis which is a shame because it could serve as a validation/calibration point for all calculations given that the present day wave heights are known. Such approach would strengthen the manuscript a lot.

My outstanding questions are:

How can increased storminess in the past be resolved if present day waves are similar?

How is the storms frequency related to the beach ridge formation and can it be informed from the clast size and the fact of preservation of past beach ridges alone?

Does the fact that a ridge is preserved mean that there was a major stormy season/s and storms were frequent or does it in fact mean that there was a period of quiescence in storms after a stormier season that lasted long enough for the GIA to uplift the area beyond the limit of the next major storm cycle?

These aspects should be at the very least addressed in the discussion.

Authors' reply:

We thank the reviewer for this thoughtful and constructive critique, which touches on the central interpretive claim of the manuscript. We believe the concern arises from a geographical distinction that was not sufficiently emphasized in the original text, and we have revised the relevant passage accordingly.

The reviewer is correct that modern wave heights in the Ross Sea can reach 6.3 m — however, these extreme values were recorded in the open Ross Sea during the PIPERS campaign, not within Terra Nova Bay. Within the Terra Nova Bay Polynya (TNBP) specifically, observed significant wave heights during strong katabatic events reach only ~2 m (SWIFT drifter data, April 2017), and numerical simulations of intense wind events under ice-free conditions yield typical values of ~3 m, with maxima occasionally exceeding 5 m. Our reconstructed wave heights —4–9 m for the well-developed ridges under sliding or rolling transport— are therefore higher than what is routinely observed in the TNBP today, and only overlap with the uppermost tail of modeled ice-free scenarios (lines 299–306). Note that the open-Ross-Sea values (SWH ~6.3 m; deep-water H_0 ~7 m) serve as hydrodynamic inputs for nearshore wave transformation calculations rather than direct comparisons with reconstructed wave heights (see Methods; lines 473–479). This distinction is now made explicit in the revised manuscript.

Regarding the reviewer's three outstanding questions:

Q1: How can increased past storminess be resolved if present-day waves are similar?

They are not similar at the relevant location. Modern TNBP observations (~2 m) fall below the wave heights required to mobilise the largest ridge-forming boulders (~4–9 m). The partial overlap with modeled ice-free maxima (~5 m) does not contradict our interpretation; it supports the premise that early Holocene conditions, characterised by reduced sea-ice cover and expanded open-water fetch, enabled wave energy regimes that are rare or absent under modern sea-ice conditions.

Q2: How is storm frequency related to beach ridge formation?

Individual ridge formation does not require a single catastrophic event; rather, we interpret each prominent ridge as the cumulative product of repeated high-energy storm episodes over centennial to millennial timescales. Clast size sets a minimum threshold for the wave energy required, but ridge preservation and spacing together constrain the tempo of high-energy coastal activity. This interpretation is now stated explicitly in the revised Discussion (lines 294–296).

Q3: Does ridge preservation indicate storminess or post-storm quiescence?

Ridge preservation requires both sufficient wave energy to emplace the clasts and subsequent relative sea-level fall via GIA to lift the ridge above the reach of later storm waves. The two conditions are not mutually exclusive: the early-to-mid Holocene was characterised by both enhanced storminess and active GIA-driven emergence. We therefore interpret the preserved ridge sequence as recording episodic high-energy emplacement events that were subsequently stabilised by ongoing isostatic uplift, a combination that is consistent with the regional deglacial chronology and does not require prolonged post-storm quiescence alone. This point is now addressed explicitly in the revised Discussion (lines 296–298).

Regarding the modern beach ridge photograph: we agree this represents a valuable calibration opportunity. Although detailed clast measurements were not conducted on the modern active beach, field observations indicate that the dominant clast size is approximately 20 cm. Applying the same hydrodynamic transport equations used for the paleo-ridges, the estimated significant wave heights required to initiate motion of these modern clasts are ~0.7 m (sliding) and ~1.2 m (rolling), well within the range of wave heights routinely observed in the TNBP (~2 m during katabatic events). This consistency confirms that the modern beach is actively reworked under present-day wave conditions, and provides an independent validation that our transport equations yield physically reasonable results. By contrast, the largest boulders on the paleo-ridges require wave heights of 4–9 m to initiate motion, substantially exceeding both modern TNBP observations and the modern beach calibration threshold, further supporting our interpretation of enhanced early Holocene wave energy. This calibration is now incorporated into the revised Discussion (lines 307–315).

Revision:

…Individual ridges are therefore best interpreted as cumulative constructional surfaces built by repeated storm episodes rather than single catastrophic events, with clast size recording the minimum wave energy threshold rather than storm frequency. Their preservation reflects the interplay between episodic high-energy emplacement and GIA-driven emergence, which progressively lifted each ridge beyond the reach of successive wave activity without requiring prolonged post-storm quiescence.

To evaluate the plausibility of these reconstructed values, we compared them against modern observational and modeled data specific to the TNBP. Within the TNBP, the PIPERS project recorded significant wave heights of ~2 m during strong katabatic wind events (SWIFT drifters, April 2017)35, and numerical simulations of ten intense wind events between 2016 and 2021 yield typical values of ~3 m, with maxima occasionally exceeding 5 m under ice-free conditions36. The reconstructed wave heights required to mobilise the largest ridge-forming boulders (4–9 m) therefore exceed what is routinely observed in the modern TNBP, and only overlap with the uppermost tail of modeled ice-free scenarios, confirming that our estimates are hydrodynamically plausible while remaining larger than present-day conditions.

This interpretation is independently supported by field observations on the modern active beach at Inexpressible Island, where dominant clast sizes of approximately 20 cm yield estimated transport thresholds of ~0.7 m (sliding) and ~1.2 m (rolling), consistent with the ~2 m wave heights routinely observed in the TNBP during katabatic events. This agreement confirms that our hydrodynamic equations produce physically reasonable results under present-day conditions. The close correspondence between modern beach calibration and observed wave heights, set against the substantially higher thresholds of the paleo-ridges, further supports our interpretation that wave energy during the early to mid-Holocene was larger than present, reflecting reduced sea-ice cover, expanded open-water fetch, and intensified atmosphere–ocean coupling.

…

…

To apply these equations, nearshore wave conditions were first derived from deep-water parameters via wave transformation. Open-ocean wave observations from the 2017 PIPERS campaign provide the necessary boundary conditions. During a major wave event in late June, the mean SWH in the open Ross Sea reached ~6.3 m, corresponding to a deep-water wave height (H_0) exceeding 7 m[43,44]. Wave periods of 6–10 s were recorded across events where SWH exceeded 4 m, consistent with WAVEWATCH III model outputs[77]. Based on these data, we adopt $H_0 = 7\ m$ and $T_0 = 6\ s$ as representative deep-water storm inputs for nearshore wave transformation at the western coast of Inexpressible Island. …

6) The detailed comments in the PDF.

Author's reply:

Q1: Equally- Extreme does not seem to be an accurate term to describe the findings. Perhaps 'Larger than present day' would be better?

We thank the reviewer for this thoughtful suggestion. We have revised the title by replacing "Modeled Extreme" with "Reconstructed Extreme," and we acknowledge the reviewer's point regarding the term "Extreme." We have retained "Extreme" in the title as it refers to the upper end of our reconstructed wave height distribution, representing episodic high-energy events that substantially exceeded routine present-day wave conditions within the Terra Nova Bay Polynya. To address the reviewer's concern, we have revised the abstract and relevant sections of the manuscript to explicitly state that the reconstructed wave heights during the early to mid-Holocene were larger than present-day values, ensuring the comparative framing is clear throughout the text.

Q2: Having read the manuscript I wonder if 'modelling' is the right term to describe the approach. It is not to detract from the work done here.

We thank the reviewer for this constructive comment. We agree that "modelling" may overstate the nature of our approach. Our methodology is based on empirical hydrodynamic equations applied to field-measured boulder dimensions, which is more accurately described as a reconstruction or estimation rather than numerical modelling. Accordingly, we have revised the title from "Modeled Extreme Wave Heights" to "Reconstructed Extreme Wave Heights," and have updated corresponding terminology throughout the manuscript to better reflect the empirical nature of our analysis.

Q3: see my comment about the ' Extreme' in the title

We thank the reviewer for this suggestion. We have revised the sentence accordingly, replacing "matched or exceeded modern extremes" with "was larger than present-day extremes" to more clearly convey the directional interpretation of our findings.

Q4: calculations or estiamtions of hydrodynamic conditions based on established empirical relationships

We thank the reviewer for this constructive suggestion. We have revised all relevant instances throughout the manuscript to accurately reflect the methodology employed.

Q5: How were the boulders selected? Were the largest boulders measured only or perhaps all boulders within an area? This matters for the results. In some way it would be resonnable to measure the biggest ones to pinpoint the maximum force and the average size to establilshe the overall storm conditions. Manual measurements often tend to be biased towards objects that are easiest to access so this needs to be clarified in the text.

We thank the reviewer for raising this important methodological point. We have clarified the boulder selection strategy in the revised text. Boulders were selected using a systematic spatial sampling approach, with at least 50 clasts measured per ridge distributed evenly along its length to minimise accessibility bias. Large boulders were deliberately included in all samples, as their dimensions are critical for constraining the maximum hydrodynamic conditions associated with extreme storm events, while the full size distribution characterises overall storm intensity.

Q6: this appears out of context. Are there till outcrops/ subcrops present in the study area? Perhaps it is needed to introduce that. Even then reworked till provides the source of boulders/erratics but it still is wave action that organises them in beach ridges.

We thank the reviewer for this insightful comment. We agree that the original phrasing lacked sufficient geological context. In the revised manuscript, we have added relevant geological background to provide clearer context for the provenance and characteristics of the ridge-forming clasts. The excluded clasts are distinguished not by their glacial origin, but by their anomalous size, angular morphology, and low roundness, which indicate they are likely in situ glacial erratics with minimal wave reworking, and thus are not representative of wave transport conditions. We have revised the sentence accordingly.

Q7: This is a huge boulder- it would be interesting to see the minimum required conditions for it to be moved by waves

We thank the reviewer for this comment. The minimum wave conditions required to mobilize boulders across the full size range (P5–P95) for each ridge are provided in the newly added Supplementary Table 9, which reports the critical flow velocity (u) and corresponding wave height (H) and wave period (T) under sliding, rolling/overturning, and saltation/lifting transport modes. For a boulder with a long axis of ~150 cm, the estimated critical wave heights are approximately 4 m (sliding), 8 m (rolling/overturning), and 33 m (saltation/lifting).

Q8: do i understand correctly that :1) boulders are measured and 2) the second largest is used as representative for wave energy calculationns? Why not use the distribution to calculate the spectrum P5-P25-P50-P75-P95 for example each representing a size along the boulder size distribution? This is likely to offer a more representatiove result.

We thank the reviewer for this insightful suggestion. Yes, your understanding is correct: boulders were measured in the field, and the original analysis used the second-largest clast as a representative size for wave energy estimation. We fully agree that this approach is suboptimal, and your suggestion to use the full size distribution is well taken. In the revised manuscript, we have replaced the single second-largest clast approach with a percentile-based analysis (P5, P25, P50, P75, and P95) applied to the complete boulder size distribution of each beach ridge. P50 provides a robust estimate of typical formative wave conditions, less sensitive to outliers than the second-largest clast, while P95 captures the extreme end of wave energy experienced by each ridge. The broad wave height range implied by the P5–P95 distribution (sliding: 0.38–4.19 m; rolling: 0.62–8.90 m) offers a more representative picture of the spectrum of storm conditions responsible for ridge construction. These revisions are reflected in the updated Supplementary Figure 3 and the revised text.

Q9: A figure showing beach location a and sample location as well as time overlap of each sample would be good here. Figure 4 does not clerly indicate the presence of samples with respect to the presence of ridges. I suggest using figure 3A and adding the dating information.

We thank the reviewer for this helpful suggestion. We agree that a clearer visual link between sample locations, ridge positions, and dating information is essential for the reader to evaluate the chronological framework. We have made several targeted revisions to address this concern. Specifically, we have added a new panel (Figure 3F) presenting a topographic cross-section of the beach ridge sequence from inland to seaward, with the four ^{10}Be-dated samples marked directly on their respective ridges (A, E, H, and O) alongside their exposure ages. This panel is accompanied by field photographs of each sampled ridge (Figures 3B–E), allowing the reader to intuitively link the geochronological data to both the morphological position and the field appearance of each ridge. In addition, Figure 4 has been updated to include ridge labels (A–O) at all relevant positions, and the precise geographic locations of all ^{10}Be and radiocarbon sampling sites are provided in Supplementary Fig. 4. We believe these revisions collectively address the reviewer's concern and make the chronological framework considerably easier to follow.

Q10: please clarify what you mean by external uncertainties. From what i can see here the range for the 9100 date is 7900-10300 whereas the 7055 data has a range of 670- 7405. both dates do not overalp based on the numbers provided. How should this be understood?

We thank the reviewer for this careful observation and for pressing for clarification — this is an important point. The key issue is that the raw ^{10}Be exposure ages carry an inherited ^{10}Be component accumulated prior to final beach deposition, most plausibly through shallow-water cosmogenic production or intermittent subaerial exposure during boulder transport. This inheritance systematically shifts the raw ages to older values. Based on ridges A, E, and O, we estimate a mean inheritance of ~1049 yr (95% CI: 0 to +2850 yr). After applying this inheritance correction, the corrected ^{10}Be ages do overlap with the ^{14}C-based elevation–age trend within the combined 1σ external uncertainties of both methods, and the apparent contradiction between the two dates is resolved. To make this transparent, we have revised Figure 4 to display 1σ external uncertainties explicitly for all four ^{10}Be ages — both raw and inheritance-corrected — so that the degree of overlap is directly visible. The main text has also been revised to clearly distinguish between raw and corrected ages and to explain the basis for the inheritance correction.

Q11: once again this should be illustrated on a figure (suggested figure 3A with clearly indicated data points and estimated/ calculated dates.

We thank the reviewer for this helpful suggestion. We note that the estimated formation ages for all beach ridges (A–O), along with their 1σ uncertainty ranges, are displayed as navy blue diamonds in Figure 4, where each ridge is individually labeled within the age–elevation framework. We hope this presentation adequately addresses the reviewer's concern.

Q12: is there a local bedrock source and is it the same lithology?

We thank the reviewer for raising this point. To address it, we have added geological background to the manuscript clarifying that the bedrock of Inexpressible Island consists predominantly of Ordovician Granite Harbor Intrusives and Precambrian metamorphic complexes of the Wilson Terrane. The ridge-forming clasts are predominantly granitic, broadly consistent with both the local Granite Harbor Intrusive bedrock and the modern beach sediments observed during fieldwork, supporting a local nearshore provenance following ice retreat. We hope this additional context adequately addresses the reviewer's concern.

Q13: can you please provide the size of the clasts for context here?

We thank the reviewer for this suggestion. We have added the clast dimensions for context. In the revised manuscript, we have included the P95 clast three-axis dimensions directly alongside the corresponding wave height estimates (e.g., Ridge A:125.0×90.0×40.0 cm; Ridge E:130.0×90.0×50.0 cm; Ridge H:180.0×60.0×40.0 cm; Ridge N:90.0×60.0×45.0 cm), allowing the reader to directly relate wave energy thresholds to clast size.

Q14: I agree that 26 m seems unrealistic. That said the recorded wave height cannot be used as an argumet/ proxy for the maximum wave height in the past especially when the paper discusses increased storminess.

We thank the reviewer for this comment. We would like to clarify that the >26 m figure is used solely to exclude saltation/lifting as a viable transport mechanism, not as a proxy for past wave heights. Our inference of enhanced Holocene storminess is based entirely on the sliding and rolling/overturning thresholds.

Q15: generally accepted insteead of widely cited?

We thank the reviewer for this suggestion and have replaced "widely cited" with "generally accepted" in the revised manuscript.

Q16: can you be more precise here?

We thank the reviewer for this suggestion. We have added specific chronological constraints to clarify this statement. Ridges above ~18 m asl (Ridges A–J) yield Bayesian fusion formation ages of 6.3–8.0 ka, which are approximately 0.5–1.0 ka earlier than the ages indicated by the reference curve at equivalent elevations. The revised sentence now reads as above.

Q17: I believe that the meltwater pulse 1A was demonstrated to mainly originate from the northern hemisphere ice sheets - for example: 10.1038/s41467-021-21990-y. What is notable here exactly- can you please elaborate?

We thank the reviewer for this comment. We agree that the independence from MWP-1A is well-established in the literature and does not represent a novel finding of this study. We have removed this sentence to avoid overstating the significance of our results and to keep the discussion focused on the early Holocene deglaciation signal recorded at Terra Nova Bay.

Q18: If I understand this correctly the follwing was estimated in this paper for the raised beach ridges:

1) Wave heinght between 3-6 m were required to move the largest boulders

2) smaller boulders required waves~2m to be moved

Present day observations, from a very time limited campaing when compared the time distance between 8-5 ka BP, documented wave heights that are comparable or even larger (6.3 and >7m) to the past conditions.

From the statements above I struggle to understand how an increased storminess, when compared to the present day conditions, can be postulated. Perhaps I have missed something but , if I did, it is not clear and needs to be outlined in the text better.

We thank the reviewer for this detailed and constructive critique. We believe the concern stems from a geographical distinction that was insufficiently emphasised in the original manuscript. The reviewer correctly notes that open-ocean SWH values of 6.3–7 m have been recorded in the Ross Sea. However, these measurements were obtained in the open Ross Sea (PIPERS campaign), not within Terra Nova Bay. Within the Terra Nova Bay Polynya (TNBP), the relevant comparison environment, observed SWH during intense katabatic events reaches only ~2 m (SWIFT drifter data, April 2017), and numerical simulations under ice-free conditions yield typical values of ~3 m, with occasional maxima approaching 5 m. Our reconstructed formative wave heights — 4–9 m for the well-developed ridges under sliding or rolling transport — exceed what is routinely observed within the TNBP today, and overlap only with the uppermost tail of modeled extreme scenarios. The open-ocean values serve exclusively as hydrodynamic boundary inputs for nearshore wave transformation, not as direct analogues for bay-interior conditions. This distinction is now made explicit in the revised Methods and Discussion sections.

<End of Summary of Changes>

Summary of Changes

The authors would like to thank the reviewers for their constructive comments and kind support. We provide below a detailed account on the changes that we have made in response to the comments that the reviewers have raised.

Response to Reviewer #4:

Regarding my comment on the choice of the second to largest clast, the updated analysis that represents the full distribution of clasts is much improved. I am very happy with how the authors have approached that issue, as well as their acknowledgement of the beaches being formed by multiple storm events rather than single events. I have no further comments or suggestions to offer.

Authors' reply:

We thank Reviewer for their positive feedback and are glad that the updated clast analysis and our discussion of multi-storm beach formation have been well received.

Response to Reviewer #5:

I commend the authors for their comprehensive response to my own comments and those of other reviewers, too. The manuscript is undoubtedly improved and, from my own perspective, it seems ready for publication.

Authors' reply:

We are grateful to Reviewer for their encouraging comments and generous assessment of the manuscript.

I'm not a MATLAB expert, but I can confirm that the code runs and generates one of the figures used in the manuscript, so it appears to function as intended.

Authors' reply:

We thank Reviewer for taking the time to test the code. We are pleased to hear that the deposited MATLAB scripts appear to run as intended.

Response to Reviewer #6:

I'm pleased to say that, following the last round of reviews most of my major concerns were comprehensively addressed and the manuscript itself is improved. It is now clearly demonstrated that the modern-day wave conditions are substantially smaller than ones in the past.

The figures now link much better to the results and it is possible to easily follow the reasoning.

I have one last comment regarding the following statement:'Sample MCB8-1 from ridge H was excluded from this estimate, as it yields an anomalously young apparent age (3.61 ± 0.75 ka) relative to both the local 14C chronology and the other 10Be samples, likely reflecting post-depositional surface disturbance'

Whereas it is likely that the sample was disturbed it is important, in my view, to highlight that the disturbance mechanism here is unknown and might have been here, among other processes, a storm activity washing over lower beach ridges (H-O) and disturbing this particular one. This in consequence yielded a younger age. other disturbance mechanism are also possible.

Authors' reply:

We thank Reviewer for this thoughtful suggestion. We agree that the disturbance mechanism for Sample NYD-MCB8-1 remains uncertain, and that the original phrasing may have implied more certainty than is warranted. Following the reviewer's suggestion, we have revised the relevant sentence to better reflect this uncertainty and to acknowledge that multiple disturbance mechanisms are possible. The revised text now reads:

"Sample NYD-MCB8-1 from ridge H was excluded from this estimate, as it yields an anomalously young apparent age (3.61 ± 0.75 ka) relative to both the local ^{14}C chronology and the other ^{10}Be samples. This anomaly is consistent with post-depositional surface disturbance, though the precise mechanism remains uncertain. Possible processes include storm waves overtopping and reworking the lower beach ridges (H–O), among other disturbance mechanisms."

<End of Summary of Changes>
